# Carboxylate‐Driven Metal Pre‑Fixation COF@MOF Synthesis: Enabling Tailored Structural, Dimensional, and Defect Engineering

**DOI:** 10.1002/advs.76204

**Published:** 2026-06-18

**Authors:** Ying Zhao, Dongmei Chen, Huilin Zheng, Min Chen, Dingtang Li, Shuyu Xie

**Affiliations:** ^1^ State Key Laboratory of Agricultural Microbiology Core Facility Huazhong Agricultural University Wuhan Hubei China; ^2^ National Reference Laboratory of Veterinary Drug Residues (HZAU) Huazhong Agricultural University Wuhan Hubei China; ^3^ Hubei Hongshan Laboratory Wuhan Hubei China; ^4^ Key Laboratory of Prevention & Control For African Swine Fever and Other Major Pig Diseases Ministry of Agriculture and Rural Affairs Wuhan Hubei China; ^5^ Frontier Science Center For Animal Breeding and Healthy Husbandry Ministry of Education Wuhan Hubei China

**Keywords:** hierarchical COF@MOF, metal pre‐fixation, H_2_O_2_ activation, CH_4_/C_2_H_6_/C_3_H_8_ separation

## Abstract

Hierarchical composites composed of covalent‐organic frameworks (COFs) and metal‐organic frameworks (MOFs) (COF@MOF), exhibiting superior tunability in terms of pore structure and electronic distribution, have gained increasing attention in various fields. However, the development of a universal strategy for controllable assembly of COF@MOF composites, enabling precise tuning of composition and structure, remains a significant challenge. Here, a flexible and adjustable COF@MOF synthesis strategy (metal pre‑fixation, MPF) is proposed, which facilitates the extensive growth of MOFs with varying morphologies and particle sizes on the surface of PY‐COF‐COOH. By employing a hypothesis‐deduction approach alongside experimental and density functional theory (DFT) calculations, systematically elucidated that the MPF strategy enables controllable modulation of morphology, size, and coordination structure through ion distribution‐guided synthesis (IDGS), a proposed working hypothesis of crystal‐facet shielding (CFS), and aperture synergistic regulation (ASR) effects. Furthermore, the MPF‐derived PY‐COF‐COOH@MOFs exhibit significantly enhanced CH_4_/C_2_H_6_/C_3_H_8_ separation performance and sustained H_2_O_2_ activation efficiency. These results demonstrate significant potential in designing high‐performance COF@MOF composites and offer a comprehensive application guide for MPF‐based design.

## Graphical abstract

A universal metal pre‐fixation (MPF) strategy directs the precise crystallization of diverse metal‐organic frameworks (Co‐MOF, ZIF‐8, Fe‐MOF, UIO‐66) on covalent organic framework (PY‐COF) scaffolds. The resulting hierarchical hybrids exhibit tunable porosity, structural defective and dimensional, as well as enhanced performance in CH_4_/C_2_H_6_/C_3_H_8_ separation and Fenton catalysis.

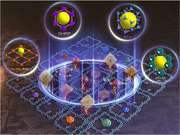

## 1 Introduction

Hierarchical porous materials designed via reticular chemistry combine precise control of microstructures (coordination motifs, pore geometry) and macrostructures (morphology) to achieve synergistic physicochemical properties [1, 2, 3]. Metal‐organic frameworks (MOFs) excel in catalysis/gas adsorption through open metal sites and tunable micropores [4, 5, 6, 7], while covalent organic frameworks (COFs) offer enhanced stability and light‐harvesting capabilities via π‐conjugated networks [8, 9]. This complementarity drives emerging strategies for MOF@COF hybridization, particularly COF‐on‐MOF heterostructures that synergistically integrate pore engineering, photonic properties, and charge transfer dynamics [10, 11, 12].

The connection method of COF@MOF hybrid materials usually depends on the chemical groups in the structure of COF or MOF. Since their debut in 2016, ‐NH_2_/‐CHO condensation reactions have dominated COF‐on‐MOF heterostructure fabrication, with alternative strategies (amide bonds, π–π stacking, C–C coupling) subsequently emerging [11, 13, 14, 15]. Two critical limitations persist: (i) Structural instability of acid‐sensitive MOFs (e.g., ZIF‐8) restricts substrate selection to acid‐resistant frameworks (e.g., UIO‐66) [12, 16, 17], narrowing MOF diversity for mild‐condition applications (e.g., gas adsorption/separation); (ii) Diffusion constraints of COF precursors within MOF pore networks compromise COF crystallinity and structural continuity [18, 19], ultimately degrading functional integrity in COF@MOF composites. More recently, coordination‐induced nucleation using pre‐installed carboxyl, sulfonate, or N‐donor groups on COFs, or through polymer‐mediated functionalization (e.g., PVP) to introduce carboxyl groups, has been explored, offering a viable route to circumvent the acid sensitivity issue and enable MOF growth under mild conditions [20, 21, 22, 23]. However, the direct incorporation of such strongly polar groups often compromises COF crystallinity, while polymer‐mediated functionalization introduces ill‐defined interfaces [22, 23]. More importantly, the absence of systematic research on COF@MOF synthesis strategies, particularly in terms of an in‐depth understanding of the morphology, pore structure, and coordination environment, has led to inadequate guidance for their application in novel fields, hindering the ability to achieve tailored designs and severely limiting the development of high‐performance COF@MOF materials.

Recently, phenolic hydroxyl groups have been strategically incorporated into COF backbones to exploit their polarity and coordination/hydrogen‐bonding capabilities. Notably, compared to strongly coordinating groups such as carboxyl or sulfonate, the phenolic ─OH group is a relatively weak coordinating moiety, which typically imposes a milder impact on COF crystallinity during the condensation process, a feature that has enabled well‐crystallized phenolic–OH‐bearing COFs to be reliably reported in the literature [24, 25, 26, 27, 28, 29]. Beyond enhancing COF properties such as hydrophilicity and metal‐ion uptake, phenolic hydroxyl units serve as versatile handles for post‐synthetic modification. We hypothesize that converting these phenolic –OH groups into carboxyl groups via a polymer‐free, site‐specific substitution reaction can create a well‐defined and highly compatible coordination environment for MOF nucleation, thereby addressing the key challenges in current COF@MOF hybridization. This carboxylate‐driven strategy offers three advantages: (i) It preserves the crystallinity of the COF substrate by avoiding direct incorporation of strongly coordinating groups into the monomer design; (ii) It provides a carboxyl‐rich interface with stable and favorable coordination affinity toward diverse MOF metal clusters; (iii) It eliminates the need for acidic catalysts, thus preserving the structural integrity of acid‐sensitive MOFs. This approach is therefore anticipated to provide a generalized route for growing diverse MOF types and morphologies on COF surfaces, substantially expanding the design space of hierarchical porous hybrid materials.

As a proof of concept, we herein propose a novel carboxyl‐group engineering strategy, termed Metal Pre‐Fixation (MPF) method, which enables precise engineering and modulation of the linker environment at the COF@MOF interface. Molecular carboxyl functionalization of PY‐COF surfaces created universal nucleation sites and well‐defined interfaces, enabling the controlled growth of four representative MOFs—ZIF‐8 (an imidazolate‐based MOF distinct from the carboxylate‐based systems, chosen to validate the universality of the MPF across different coordination chemistries), Fe‐MOF, UIO‐66, and Co‐MOF—with precise tunability over their morphology and particle size. Furthermore, using a series of synthesized COF@MOFs, we systematically investigated the features of the MPF strategy. Our results emphasize that the MPF strategy enables the regulation of the morphology, pore structure and coordination defects of COF@MOF hybrid materials through ion distribution‐guided synthesis (IDGS), a proposed working hypothesis of crystal facet‐shielding (CFS) effects, and aperture synergistic regulation (ASR) effects. We note that these three terms are introduced as a descriptive and interpretive framework grounded in our experimental observations, rather than as claims of fundamentally new physical principles. Additionally, the significant improvement in CH_4_/C_2_H_6_/C_3_H_8_ adsorption separation (PY‐COF‐COOH@ZIF‐X) and catalytic degradation (PY‐COF‐COOH@Co‐MOF‐X) performance proved that the MPF strategy has exciting application prospects. This study provides comprehensive guidelines for designing COF@MOF hierarchical materials via MPF strategy and opens up more possibilities for the design of multifunctional COF@MOF materials.

## 2 Results and Discussion

### 2.1 Fabrication and Characterization of PY‑COF@ZIF‑X Composites

**SCHEME 1:**
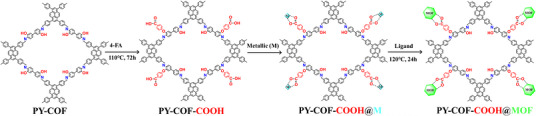
Synthesis process of PY‐COF‐COOH@MOF.

**FIGURE 1:**
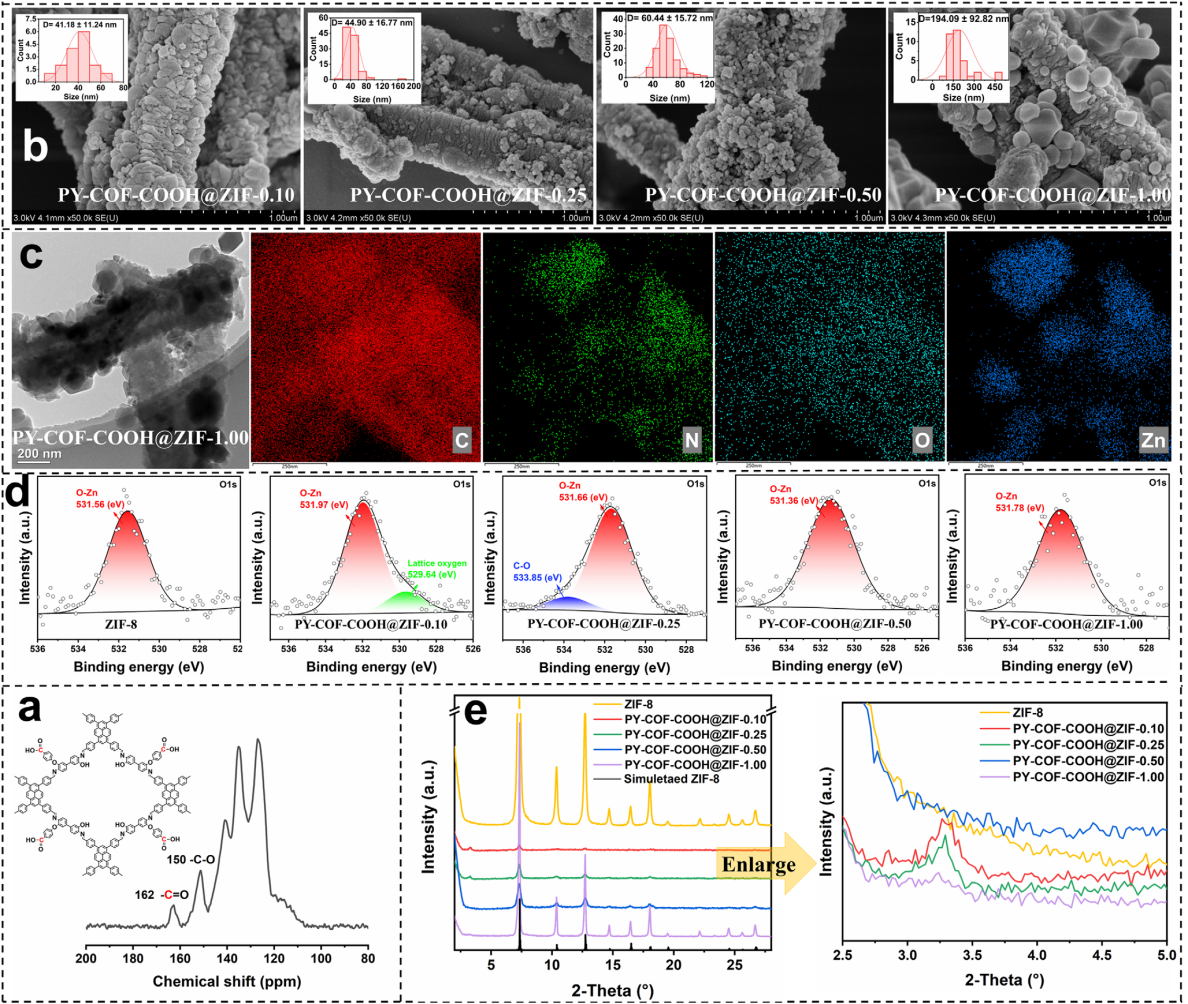
(a) SS‐NMR results of PY‐COF‐COOH. (b) SEM images of PY‐COF‐COOH@ZIF‐X (X=0.10, 0.25, 0.50, and 1.00). (c) HR‐TEM images of PY‐COF‐COOH@ZIF‐1.00. (d) O1s spectrum of PY‐COF‐COOH@ZIF‐X (X=0.10, 0.25, 0.50, and 1.00). (e) PXRD patterns of PY‐COF‐COOH@ZIF‐X (X = 0.10, 0.25, 0.50, and 1.00).

To assess the feasibility of the MPF strategy, we employed a post‐synthetic modification approach to construct an acid‐sensitive ZIF‐8 coating on the surface of a highly conjugated, rod‐like PY‐COF, designating the resulting composite as PY‐COF‐COOH@ZIF. Scheme 1 depicts the synthetic workflow. First, the PY‑COF was prepared following the procedure reported by Li et al. [30], affording a material with a BET surface area of 486.46 m^2^ g^−1^ and a pore diameter of 3.58 nm. SEM and HR‐TEM (Figure S1), powder X‐ray diffraction (PXRD) (Figure S2), and N_2_ adsorption‒desorption (Figure S3) confirmed the successful synthesis of PY‐COF. Then, PY‐COF was modified with 4‐fluorobenzoic acid (4‐FA) to introduce carboxyl groups as metal fixation sites (PY‐COF‐COOH). SS‐NMR, FTIR, and XPS confirmed successful modification (Figures S4 and S5), with SS‐NMR (Figure 1a) showing characteristic peaks for CO (160 ppm) and O‐AR (152 ppm). Batch‐to‐batch reproducibility was ensured by an excess reagent strategy (1.0 mmol 4‐FA per 100 mg PY‐COF), with XPS C 1s gradient analysis confirming saturation of the carboxyl modification (Figure S5). Subsequently, Zn^2+^ and 2‐methylimidazole were sequentially added to yield PY‐COF‐COOH@ZIF.

To probe the structural evolution under varied synthetic conditions, we established a concentration gradient of Zn^2+^ precursors (X = 0.10, 0.25, 0.50, 1.00 mmol) during ZIF‐8 growth. And the key growth behaviors of ZIF‑8 on the PY‑COF surface were subsequently elucidated through multimodal characterization. SEM images (Figure 1b) show ZIF‐8 nanoparticles uniformly anchored on the PY‐COF‐COOH surface, exhibiting markedly improved dispersibility compared to aggregated pristine ZIF‐8 (Figure S6), attributable to the homogeneous distribution of carboxyl sites on the PY‑COF‐COOH surface that serve as metal pre‑fixation anchors. HR‐TEM and Elemental mapping (Figure 1c and Figure S7) further confirm the homogeneous distribution of Zn (from ZIF‐8) and O (from PY‐COF‐COOH), while revealing that ZIF‐8 surface coverage increases with X and approaches saturation at X = 0.50. Quantitative ICP‐OES analysis (Figure S8) reveals a monotonic increase in ZIF‐8 mass fraction from 8.45 to 95.17 wt.% across the X range, and TGA (Figure S9) corroborates the low loading at X = 0.10 but indicates non‐additive thermal behavior at higher loadings, likely due to the nearly saturated ZIF‐8 shell retarding PY‐COF‐COOH decomposition. This trend is supported by O 1s X‐ray photoelectron spectroscopy (XPS, Figure 1d): the characteristic peak at 529.4 eV, assigned to non‐coordinating lattice oxygen in PY‐COF‐COOH, emerges at X = 0.10 and progressively attenuates with increasing ZIF‐8 loading (X value). Notably, ZIF‐8 particles in PY‐COF‐COOH@ZIF‐1.00 exhibited anomalous growth (194.09 nm vs. 44.18–60.44 nm for X = 0.10–0.50, labeled as Phenomenon 1). Since synthesis variables were strictly limited to precursor concentration, and all reactions were conducted under identical temperature and duration conditions, the observed size anomaly cannot be attributed to these kinetic factors. Control experiments revealed that pristine ZIF‐8 synthesized under the same conditions maintained sizes below 50 nm, comparable to those in PY‐COF‐COOH @ZIF‐X (X = 0.10‐0.50). This stark contrast suggests that the anomalous growth in PY‑COF‐COOH @ZIF‑1.00 arises from structural confinement effects mediated by uniformly distributed ‐COOH sites on the PY‑COF‐COOH surface, which become dominant at high precursor concentrations and likely modulate crystallization pathways or coordination dynamics in the MPF strategy.

To directly verify the essential role of carboxyl functionalization in this anomalous growth, we conducted parallel control experiments using unmodified PY‐COF (PY‑COF@ZIF‐X). SEM, HR‐TEM, and elemental mapping (Figures S10 and S11) reveal sparse and non‑uniform ZIF‑8 attachment on unmodified PY‑COF, with particle sizes consistently 30.13–34.94 nm (Figure S12) across X = 0.10–1.00, suggesting that carboxyl modification may be a key factor in inducing the anomalous growth. To further probe the nucleation pathway, we performed metal‑ion incubation experiments: both PY‑COF‑COOH and unmodified PY‑COF were exposed to Zn^2+^, Co^2+^, Fe^3+,^ and Zr^4+^ for 30 min under synthesis‑relevant conditions, followed by three washing cycles and elemental mapping (Figures S13 and S14). PY‑COF‑COOH retained significantly more Zn^2+^ (0.8 %), Co^2+^ (0.7%), and Fe^3+^ (0.3%) than the unmodified counterpart (0.0%, 0.1%, and 0.2%, respectively), directly confirming that carboxyl functionalization creates local metal‑ion enrichment at the PY‐COF surface. Crucially, Quasi‑time‑resolved HR‐TEM of PY‑COF‐COOH@ZIF‑1.00 (withdrawn at 1, 2, 4, 8, 12, 24 h, Figures s15–s17) revealed a clear time‑dependent increase in ZIF‑8 particle size with progressive narrowing of the size distribution, indicative of a surface‑confined, coordinated growth process. Collectively, these results establish that carboxyl groups serve as primary metal‑anchoring sites, inducing local Zn^2+^ enrichment that governs nucleation density and subsequent crystallization dynamics.

Guided by prior studies indicating that ZIF‑8 crystallization proceeds via metastable intermediate clusters (MICs) governed by Zn^2+^/ligand pre‑equilibrium [31], we propose that Phenomenon 1 originates from an “ion distribution‑guided synthesis” (IDGS) effect within the MPF strategy, with comprehensive mechanistic analysis and predictive modeling of these interfacial dynamics detailed in subsequent sections. In this scenario, the carboxyl functionalization significantly enhances PY‑COF‑COOH's metal‑binding affinity, concentrating Zn^2+^ on the framework surface to promote MIC nucleation. At X = 0.50, MICs saturation initiates epitaxial growth, which at X = 1.00 leads to markedly larger ZIF‑8 crystallites. This anomalous crystallization behavior diverges from conventional ZIF‑8‑based COF@MOF systems [18, 32], underscoring the distinctiveness of the MPF strategy.

To correlate this growth mechanism with the structural response of the hybrids, we examined the crystal evolution of PY‐COF‐COOH@ZIF‐X (X = 0.10–1.00) using powder X‑ray diffraction (PXRD). As shown in Figure 1e, the characteristic ZIF‐8 peaks at 2θ = 7.43° (110), 10.42° (200), and 12.77° (211) intensified linearly with increasing X values, directly correlating with the ZIF‐8 density on PY‐COF‐COOH surfaces. Notably, the PY‐COF characteristic peak (2θ = 3.28°) showed X‐dependent behavior, it remained detectable at X = 0.10/0.25 but vanished at X≥0.50 (labeled as Phenomenon 2). The peak retention at low X values confirmed PY‐COF‐COOH's stability under synthesis conditions, supported by control experiments (Figure S18).

This PXRD anomaly prompts comparison with established COF@MOF architectures. As reported, typical MOF‐on‐COF composites exhibit dual‐phase diffraction patterns [12, 33, 34]. In contrast, the complete extinction of PY‑COF signals in PY‑COF‐COOH@ZIF‑X (X = 0.50/1.00) is distinct from conventional MOF‑in‑COF systems, where ZIF‑8 peak disappearance is typically attributed to pore‑confinement effects [18]. Instead, the observed pattern aligns with characteristics of core–shell architectures [35, 36]: when the highly crystalline ZIF‐8 shell achieves complete surface coverage (X ≥ 0.50), it creates an X‐ray‐opaque layer exceeding the penetration depth, thereby obscuring the PY‐COF signal. Combined with morphological evidence, we preliminarily attribute the PY‐COF peak disappearance to ZIF‐8's saturated surface coverage (labeled as physical shielding effect) rather than structural degradation (Although subsequent studies reveal inconsistencies in the physical shielding interpretation).

Together, these results not only validate the MPF strategy as a mild platform for constructing metastable COF@MOF composites, but also highlight the presence of non‐classical growth behaviors. Unravelling the mechanistic origins of these anomalous phenomena will require extending the MPF approach to a broader library of MOF structures.

### 2.2 Verification and Generality of the Ion Distribution‑Guided Synthesis (IDGS) Effect

To further validate the dose–size growth mechanism observed for ZIF‑8 on PY‑COF‐COOH (Phenomenon 1), we designed a mechanistic study by growing Fe‐MOF (a MOF with morphology switching between octahedral MIL‐101 and pencil‐shaped MIL‐88B) on PY‐COF‐COOH, forming PY‐COF‐COOH@Fe‐MOF‐X (X = 0.10–1.00). Based on the established MIL‐88B formation criterion (metal‐to‐ligand ratio >1:1 and temperature ≥120°C) [37], we hypothesized that the IDGS mechanism would induce localized Fe^3+^ enrichment on PY‐COF‐COOH surfaces at nominally equimolar precursor ratios (1:1), creating Fe‐rich microdomains to drive MIL‐88B formation specifically at high X values. Control experiments synthesizing Fe‐MOF under standard conditions yielded only MIL‐101 (Figure S19), confirming the necessity of microenvironmental modulation.

**FIGURE 2:**
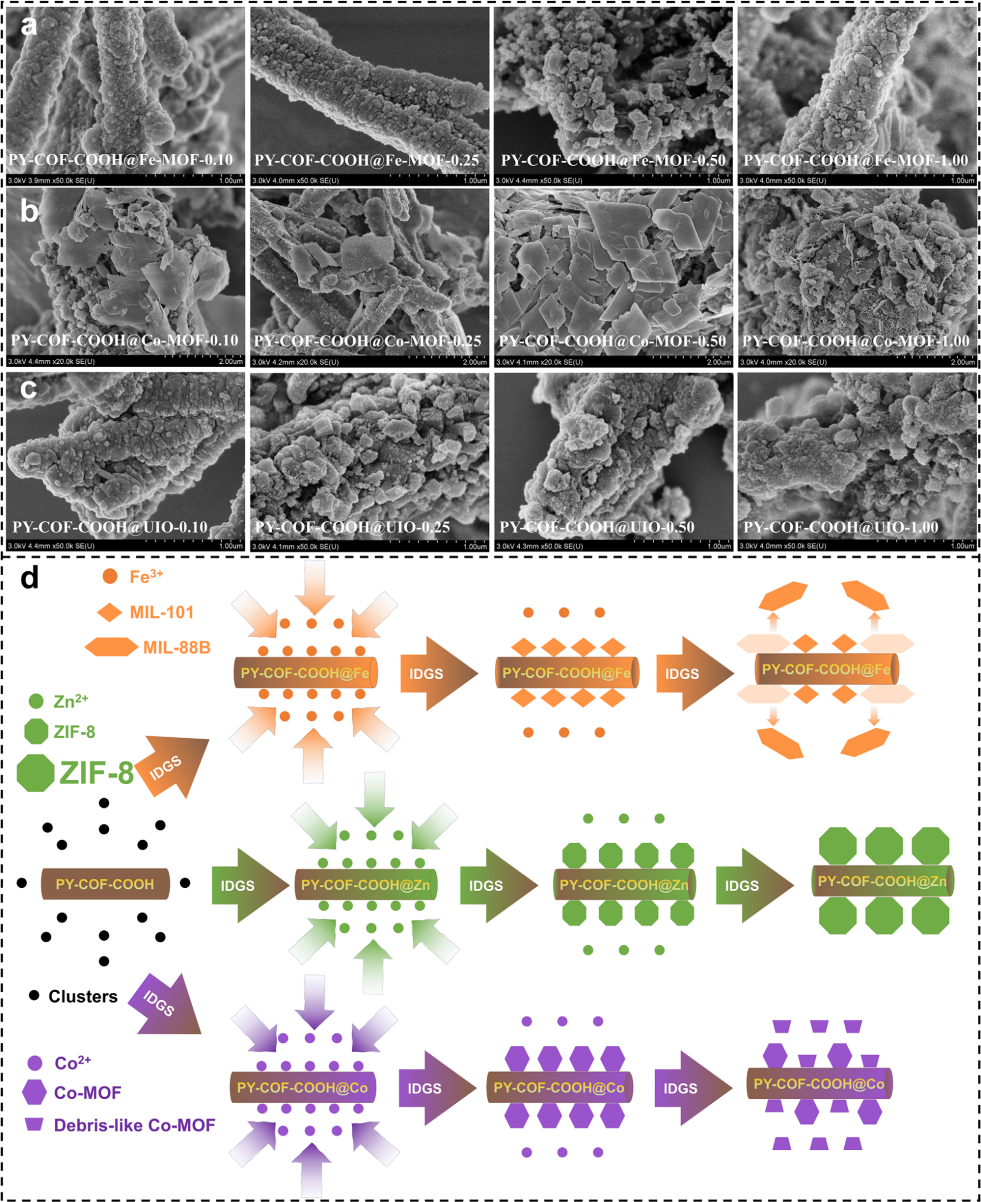
(a) SEM images of PY‐COF‐COOH@Fe‐MOF‐X (X = 0.10, 0.25, 0.50, and 1.00). (b) SEM images of PY‐COF‐COOH@Co‐MOF‐X (X = 0.10, 0.25, 0.50, and 1.00). (c) SEM images of PY‐COF‐COOH@UIO‐X (X = 0.10, 0.25, 0.50, and 1.00). (d) Schematic diagram of the different manifestations of IDGS effect in PY‐COF‐COOH@Fe‐MOF‐X, PY‐COF‐COOH@ZIF‐X, and PY‐COF‐COOH@Co‐MOF‐X.

SEM, TEM and PXRD analyses revealed three critical trends: (1) Fe‐MOF density on PY‐COF‐COOH increased with X, approaching saturation at X = 0.50 (Figure 2a, Figures S20 and S21). (2) Phase evolution followed X‐dependent hierarchy: X = 0.10/0.25: amorphous Fe‐MOF particles dominated; X = 0.50: co‐existence of MIL‐101, nascent MIL‐88B (∼2 µm), and residual PY‐COF‐COOH (Figure S22); X = 1.00: surface‐anchored Fe‐MOF particles surrounded by abundant MIL‐88B (Figure S23). (3) Crystallinity transition validated IDGS: low X samples (X = 0.10/0.25) lacked sufficient secondary building units (SBUs) for MIL‐101 crystallization, forcing amorphous growth, while high X values (≥0.50) enabled IDGS‐driven Fe^3+^ accumulation, establishing metal‐rich zones (Fe/ligand >1:1) that nucleated MIL‐88B (Figure S24). This systematic progression conclusively demonstrates that the MPF strategy achieves controlled surface growth through IDGS‐mediated microenvironment engineering, resolving both phase selection and morphological evolution in hybrid systems.

Subsequently, to investigate the universality of the IDGS effect, we strategically selected two distinct MOF archetypes: lamellar Co‐MOF (flexible framework model) and UIO‐66 particles (rigid framework model) for growth on PY‐COF‐COOH surfaces. As the precursor ratio (X) increased from 0.10 to 1.00, the growth density of Co‐MOF on PY‐COF‐COOH rose markedly, approaching saturation at X = 0.50 (Figure 2b and Figures S25,S26). Notably, excessive precursor loading (X = 1.00) induced the fragmentation of the Co‐MOF structure, generating abundant surface debris—a characteristic manifestation of the IDGS effect under supersaturation conditions. Contrasting behavior was observed in the PY‐COF‐COOH@UIO‐X series (X = 0.10–1.00), where the robust Zr_6_O_4_(OH)_4_ clusters in UIO‐66 effectively resisted deformation (Figure 2c and Figures S27–S29) [38]. This resilience persisted even upon further increasing the precursor loading (X = 2.0/4.0), maintaining uniform particle morphology across approximately four orders of magnitude in precursor concentration (Figures S30,S31). This mechanical resilience‐maintained framework integrity across four orders of magnitude in precursor concentration, demonstrating the critical role of cluster strength in modulating IDGS‐driven morphological evolution.

Collectively, these findings establish that cluster mechanical strength is the primary determinant of IDGS—mediated morphological evolution. Flexible frameworks are prone to supersaturation—driven deformation, while rigid clusters can suppress such deformation even under destabilizing conditions. The IDGS effect leads to system‐specific outcomes, as observed in PY‐COF‐COOH@ZIF‐X, PY‐COF‐COOH@Fe‐MOF‐X, PY‐COF‐COOH@Co‐MOF‐X, and PY‐COF‐COOH@UIO‐X. This indicates that COF@MOF hybrids can follow distinct evolutionary pathways, which not only explains the contrasting behaviors in archetypal systems but also suggests potential regimes for defect control and morphology engineering in COF@MOF materials.

### 2.3 Defect Engineering and Crystal Facet‐Shielding Effect

Following mechanistic confirmation of the IDGS effect, we interrogate whether morphological deformations propagate structural defects in MOF architectures and, critically, whether such defect generation can be systematically regulated. To explore this question, we conducted a comparative PXRD analysis across PY‐COF‐COOH@Fe‐MOF‐X, PY‐COF‐COOH@UIO‐X, and PY‐COF‐COOH@Co‐MOF‐X systems. The results suggest that defect states are both generated and, in several regimes, rendered controllable by precursor loading and phase evolution.

In PY‐COF‐COOH@Fe‐MOF‐X, MIL‐101‐to‐MIL‐88B phase transitions (X = 0.50/1.00) induce reversible 3.28° PY‐COF peak attenuation (Figure S24), demonstrating programmable defect density through dimensional confinement. In PY‐COF‐COOH@UIO‐X (Figure S32), the emergence of broadened reflections at 7.40° at X = 0.50/1.00 evidences defect‐rich UIO‐66, whereas their absence at X = 0.10/0.25 underscores a threshold behavior that enables defect dosing via MIC availability. In PY‐COF‐COOH@Co‐MOF‐X (X = 0.50/1.00), attenuation of Co‐MOF reflections alongside persistent PY‐COF signals is consistent with defect‐mediated X‐ray permeation or incomplete ordering within the Co‐MOF shell (Figure S34), further supporting controllable defect manifestation under growth‐limited conditions. Such precision in hierarchical defect engineering unlocks routes to dynamically organize catalytic centers and binding pockets within hybrid frameworks [39, 40, 41].

However, closer comparative analysis reveals a deeper inconsistency: the divergent evolution of the 2θ = 3.28° PY‐COF reflection across PY‐COF‐COOH@M‐MOF‐X (M = Zn/Fe/Zr/Co) systems exposes the limitations of the previously invoked physical shielding model. Specifically, the near‐complete suppression of this signal in PY‐COF‐COOH@UIO‐X (X ≥ 0.50) stands in sharp contrast to its persistent presence in PY‐COF‐COOH@Co‐MOF‐X, indicating the need for an additional regulatory mechanism [42, 43]. To reconcile these observations, we propose the crystal facet–shielding (CFS) hypothesis: preferential orientation and localized coverage of specific PY‐COF facets by MOF intermediates selectively modulate the COF diffraction intensity, in a manner decoupled from the bulk crystallinity or overall defect density.

To experimentally test this CFS hypothesis, the PY‐COF‐COOH@Co‐MOF composite emerges as a uniquely informative model system. The anomalous persistence of the PY‐COF diffraction peak at 2θ = 3.28°, alongside the concurrent disappearance of the Co‐MOF diffraction signals, provides a distinct opportunity to decouple CFS effects from structural defects. To elucidate the underlying CFS hypothesis, we therefore conducted multi‐dimensional characterization of the PY‐COF‐COOH@Co‐MOF‐X system, aiming to independently verify whether the composites simultaneously possess structural defects and high crystallinity.

**FIGURE 3:**
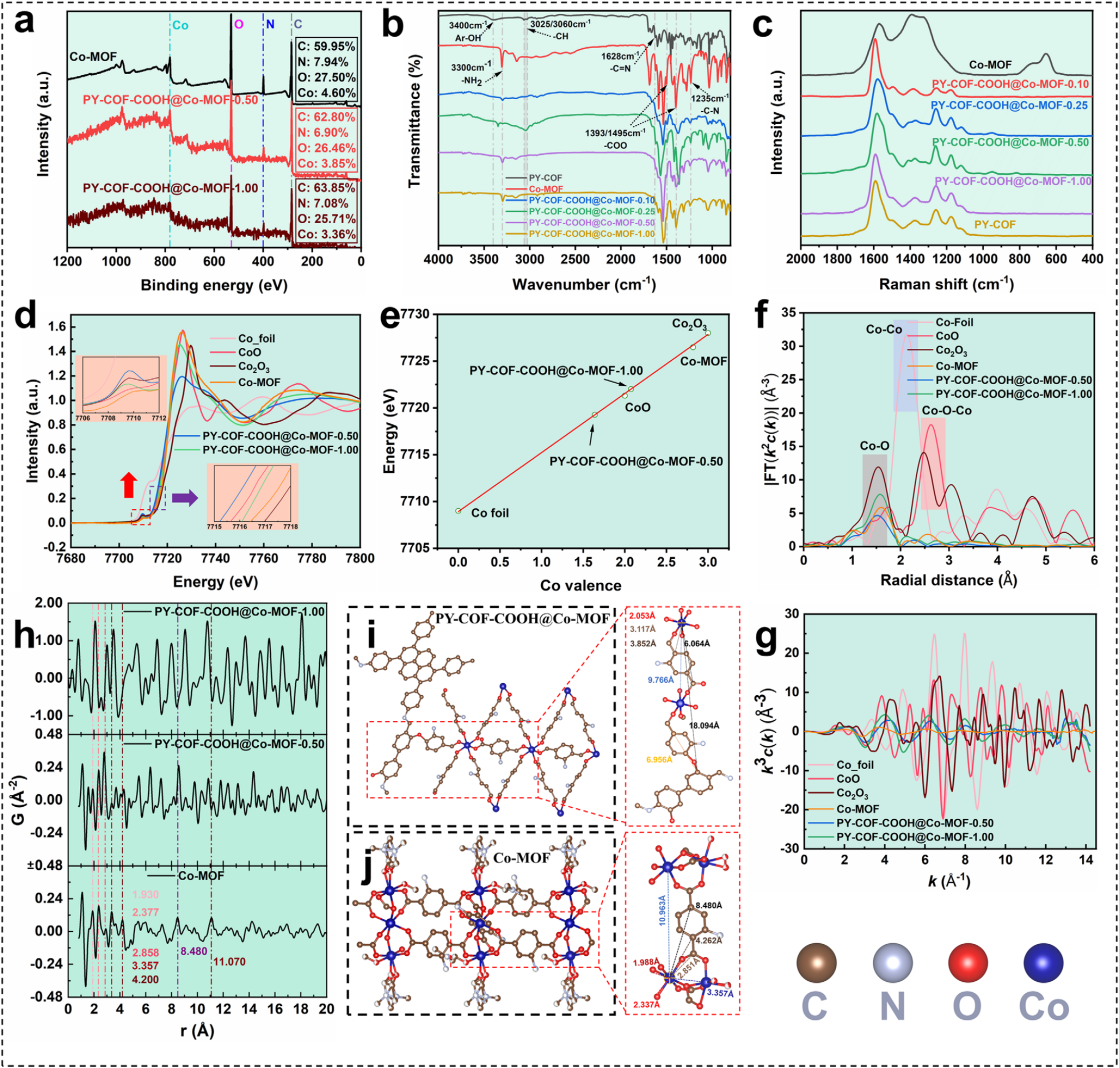
(a) XPS survey spectrum of Co‐MOF, PY‐COF‐COOH@Co‐MOF‐0.50, and PY‐COF‐COOH@Co‐MOF‐1.00. (b, c) FT‐IR/Raman spectrum of Co‐MOF, PY‐COF, and PY‐COF‐COOH@Co‐MOF‐X (X = 0.10, 0.25, 0.50, and 1.00). (d) Normalized Co K‐edge XANES spectra of Co‐MOF, PY‐COF‐COOH@Co‐MOF‐0.50, and PY‐COF@Co‐MOF‐1.00. (e) Valence of various Co species obtained from Co K‐edge XANES. (f) Fourier‐transformed Co K‐edge EXAFS spectra and corresponding fitting curves for Co‐MOF, PY‐COF‐COOH@Co‐MOF‐0.50, and PY‐COF‐COOH@Co‐MOF‐1.00. (g) Co K‐edge XAFS k^3^(χ(k)) oscillation curves of the Co‐MOF, PY‐COF‐COOH@Co‐MOF‐0.50, and PY‐COF‐COOH@Co‐MOF‐1.00. h, Experimental neutron structure factor F(Q) data of Co‐MOF, PY‐COF‐COOH@Co‐MOF‐0.50, and PY‐COF‐COOH@Co‐MOF‐1.00. (i, j) Local coordination structure diagrams of Co‐MOF (CCDC number: 1016535) and PY‐COF‐COOH@Co‐MOF.

XPS measurements revealed a systematically increasing O/Co ratio in PY‐COF‐COOH@Co‐MOF‐X with rising precursor loading (X) (Figure 3a and Figure S35), suggesting progressive introduction of structural defects [44, 45]. FT‐IR and Raman spectra provided further evidence: characteristic vibrational modes of both Co‐MOF and PY‐COF components appeared in the composite with slight blue shifts (Figure 3b), indicative of shortened bond lengths potentially associated with coordinatively unsaturated Co sites. Notably, the disappearance of the Co─O Raman vibration at 657 cm^−1^ (Figure 3c) implies disrupted metal‐carboxylate coordination, consistent with defect formation.

Direct evidence for oxygen vacancies emerged from electron spin resonance spectroscopy. The ESR signal at g = 2.003 (Figure S36), characteristic of unpaired electrons in oxygen vacancies, intensified significantly at higher X values, with PY‐COF‐COOH@Co‐MOF‐1.00 showing the strongest response. This trend was corroborated by XPS analysis (Figure S37), where the proportion of surface‐active oxygen species at 531.70 eV substantially increased in the composites compared to pristine Co‐MOF [46].

X‐ray absorption spectroscopy (XAS) at the Co K‐edge provided atomic‐level evidence of defect‐induced electronic restructuring. The attenuated white‐line intensity (7725 eV) and enhanced pre‐edge features (7710 eV) in PY‐COF‐COOH@Co‐MOF‐X (X = 0.50/1.00) relative to pristine Co‐MOF (Figure 3d,e) confirm coordination unsaturation and reduced site symmetry, which is consistent with FT‐IR/Raman signatures of bond contraction. Valence state analysis revealed controlled reduction of Co oxidation from +2.82 (pristine Co‐MOF) to +1.58 (PY‐COF‐COOH@Co‐MOF‐X, X = 0.50), directly modulated by precursor stoichiometry (Figure 3e and Table S1). Crucially, FT‐EXAFS analysis (Figure 3f,g) demonstrated preserved Co─O coordination geometry despite extensive defect incorporation, establishing that defect generation occurs through ligand vacancy formation rather than coordination environment collapse.

Collectively, these results identify oxygen vacancies and ligand vacancies as the primary defect species in the PY‐COF‐COOH@Co‐MOF‐X composites. The ESR signal at g = 2.003 and the increase in surface‐active oxygen (XPS 531.70 eV) directly confirm oxygen vacancies, while the systematic O/Co ratio increase, bond‐length contraction (Raman, EXAFS), and preservation of Co─O coordination geometry (EXAFS) collectively point to carboxylate ligand deficiency rather than global structural collapse. Although a minor degree of local structural distortion cannot be excluded, the data are most consistently explained by a combination of oxygen vacancies and missing‐linker defects, whose density is tunable via precursor stoichiometry.

Subsequently, pair distribution function (PDF) analysis resolved the paradoxical coexistence of long‐range order and defect proliferation. While Bragg scattering suggested amorphization through missing Co‐MOF reflections (Figure S34), persistent high‐intensity oscillations in PDF profiles (Figure 3h,i) demonstrated structural coherence up to 20 Å. Bond‐length contraction (Co─O: 2.337→2.148 Å, CCDC number: 1016535) directly correlated with vibrational spectroscopy shifts, confirming that vacancy‐mediated lattice contraction occurs without coordination environment collapse, a finding fully consistent with XAS coordination geometry preservation. Crucially, the maintenance of sharp oscillations at 12–20 Å interfacial distances (Figure 3j) indicates that the MPF‐induced defect engineering preserves long‐range structural coherence while accommodating localized bond distortion. Together with the EXAFS and ESR results, the PDF data support a scenario where defect formation occurs predominantly through oxygen and ligand vacancies rather than full amorphization, consistent with the proposed mechanism of controlled defect generation via carboxyl‐mediated coordination modulation.

Collectively, the apparent amorphization suggested by missing Bragg reflections in PXRD contrasts sharply with the preserved long‐range order revealed by PDF analysis [47]. This discrepancy challenges the physical shielding effect and underscores the limitation of relying solely on Bragg diffraction for structural assessment. The CFS mechanism proposed herein—where MOF intermediates undergo facet‐selective nucleation, growth, and eventual detachment due to steric constraints—provides a unified explanation for the system‐dependent visibility of PY‐COF‐COOH diffraction. Unlike particulate ZIF‐8 or UIO‐66, which fully cover PY‐COF‐COOH surfaces and attenuate its diffraction signals, lamellar Co‐MOF and pencil‐shaped MIL‐88B detach upon reaching critical size, thereby re‐exposing the underlying PY‐COF‐COOH facets. This process preserves the crystallinity of both components while enabling defect engineering, establishing a new paradigm for designing hierarchically ordered hybrid frameworks.

**FIGURE 4:**
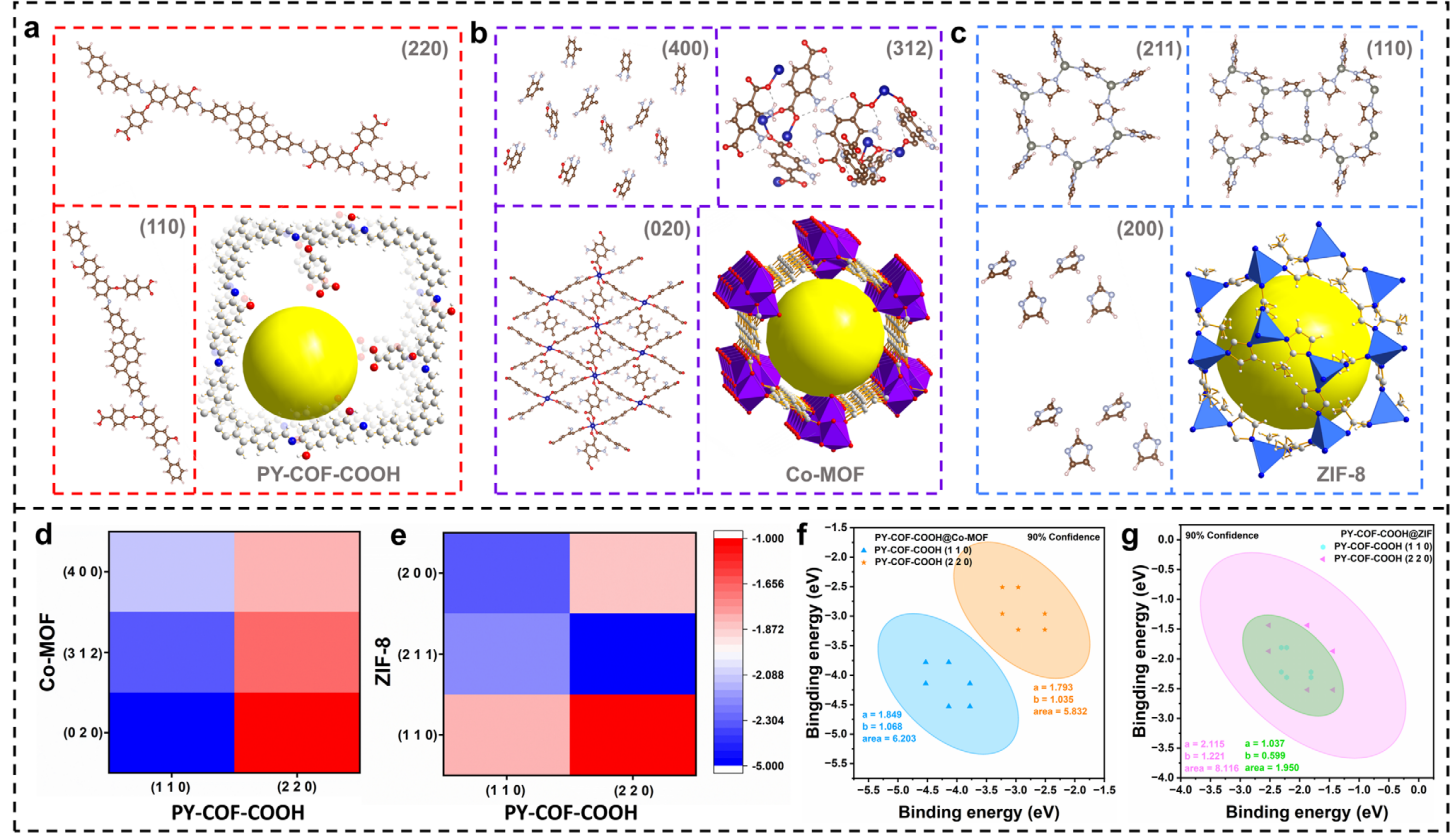
(a–c) Crystal face model of PY‐COF‐COOH ((110) and (220)), Co‐MOF ((400), (312) and (020)) and ZIF ((211), (110) and (200)). (d) Results of the crystal plane binding energy of PY‐COF‐COOH and Co‐MOF. (e) Results of the crystal plane binding energy of PY‐COF‐COOH and ZIF‐8. (f,g) Analysis results of the difference in crystal plane binding energy between PY‐COF‐COOH/Co‐MOF and PY‐COF‐COOH/ZIF‐8, respectively.

Computational binding energy analyses across crystal facets provide atomistic validation of the CFS mechanism. Exothermic binding (all ΔE < 0) between PY‐COF‐COOH and Co‐MOF/ZIF‐8 (Figure 4a–c and Table S2) corroborates interfacial interactions observed by electron microscopy. Crucially, facet‐selective thermodynamics dictate the CFS hierarchy: PY‐COF‐COOH (110) exhibits 2.8 times stronger affinity for Co‐MOF ((020)/ (312)/ (400)) facets compared to PY‐COF‐COOH (220) (Figure 4d,e), as quantified by the 90% confidence interval separation in binding energy distributions (Figure 4f). This contrasts sharply with the undifferentiated binding profile in PY‐COF‐COOH /ZIF‐8 systems (Figure 4g), where facet‐agnostic interactions enable complete surface encapsulation. The thermodynamic preference for PY‐COF‐COOH (110)/Co‐MOF interfaces rationalizes the preserved diffraction visibility through steric detachment, while ZIF‐8's non‐selective binding perpetuates signal suppression. These findings indicate that the CFS hypothesis could serve as a potential strategy for designing hierarchical frameworks with a programmed defect–crystallinity balance.

### 2.4 Aperture Synergistic Regulation Effect and Application Evaluation of the MPF Strategy

The presence of hierarchical porosity is one of the pivotal features underpinning the attractiveness of COF@MOF composites. To this end, we selected two distinct composite systems to probe the interplay between structure and function. The high surface area, microporous PY‐COF‐COOH@ZIF‐X series was employed to assess CH_4_/C_2_H_6_/C_3_H_8_ separation, while the long‐range ordered, defect‐rich PY‐COF‐COOH@Co‐MOF‐X architecture served as a catalyst to evaluate its efficacy in heterogeneous advanced oxidation processes. These targeted investigations establish clear structure‐property relationships and demonstrate the broad utility of the MPF strategy.

**FIGURE 5:**
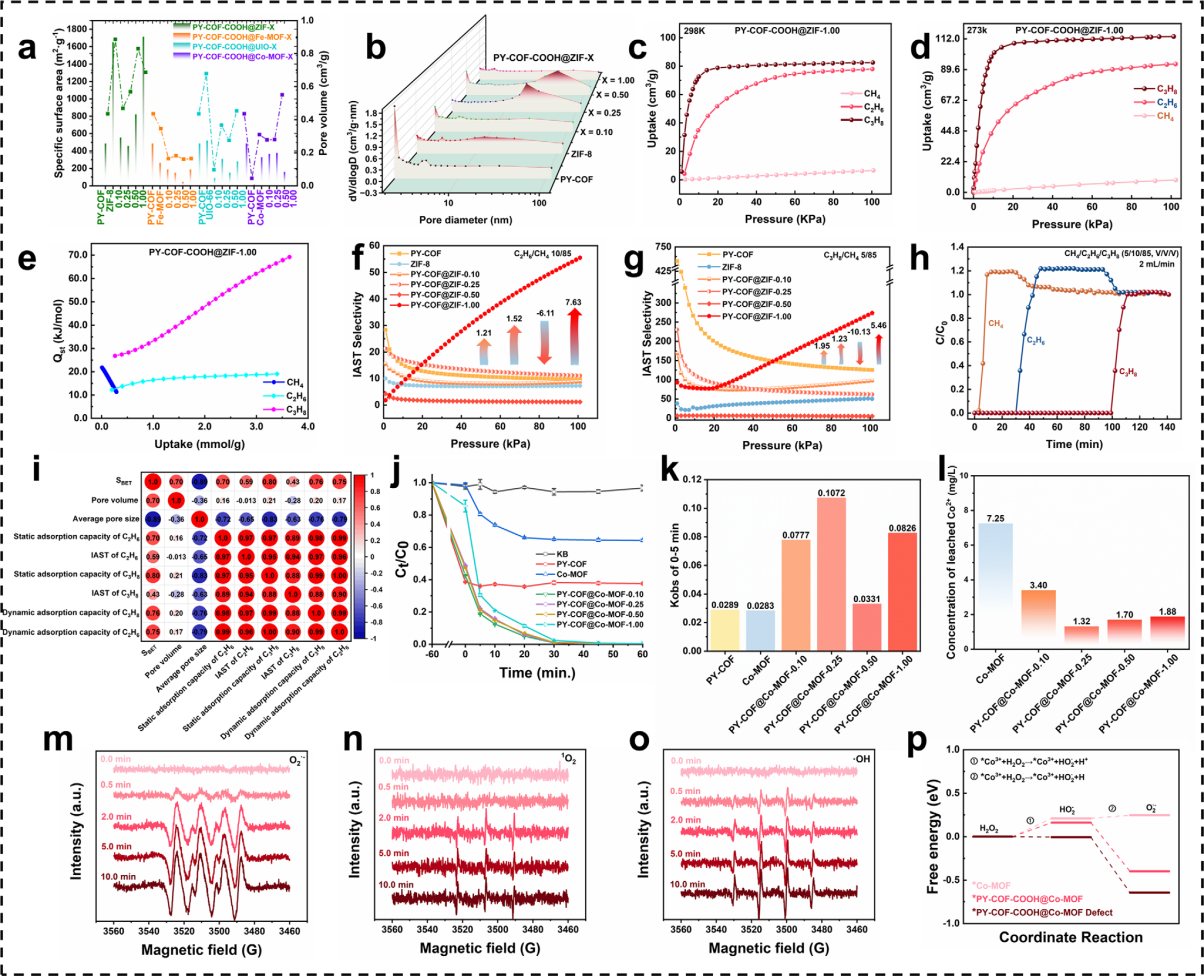
(a) The specific surface area and pore volume of PY‐COF‐COOH@ZIF‐X, PY‐COF‐COOH@Fe‐MOF‐X, PY‐COF‐COOH@UIO‐X and PY‐COF‐COOH@Co‐MOF‐X (X = 0.10, 0.25, 0.50, and 1.00). (b) Pore distribution of PY‐COF, ZIF‐8, and PY‐COF‐COOH@ZIF‐X (X = 0.10, 0.25, 0.50, and 1.00). (c, d) CH_4_/C_2_H_6_/C_3_H_8_ adsorption isotherm of PY‐COF‐COOH@ZIF‐X (X = 0.10–1.00) at 298 and 273K, respectively. e, The Q_st_ of CH_4_/C_2_H_6_/C_3_H_8_ in PY‐COF‐COOH@ZIF‐1.00. (f, g) IAST selectivity of C_2_H_6_/CH_4_ and C_3_H_8_/CH_4_ of PY‐COF‐COOH@ZIF‐X (X=0.10–1.00). (h) The experiment breakthrough curves of CH_4_/C_2_H_6_/C_3_H_8_ (5/10/85) gas mixtures with a flow of 2.0 mL min^−1^ through PY‐COF‐COOH@ZIF‐1.00 packed column at 298K and 100 kPa. (i) The Pearson correlation analysis results of structural parameters and performance parameters. (j) The Fenton oxidation degradation ENR curve of PY‐COF‐COOH@Co‐MOF‐X (X = 0.10–1.00), (Catalyst: 0.20 g L^−1^, Concentration of ENR: 20 mg L^−1^, Concentration of H_2_O_2_: 30 mM). (k) Kobs of 0–5 min of of PY‐COF‐COOH@Co‐MOF‐X (X = 0.10–1.00). (l) The Co ion leakage concentrations of Co‐MOF and PY‐COF‐COOH@Co‐MOF‐X (X = 0.10, 0.25, 0.50, and 1.00) after the Fenton reaction. (m–o) EPR spectra in Fenton/PY‐COF‐COOH@Co‐MOF‐0.50 system. (p) The free energy step diagram of the catalytic production of O_2_^·−^ by Co‐MOF, PY‐COF‐COOH@Co‐MOF, and defective PY‐COF‐COOH@Co‐MOF from H_2_O_2_, respectively.

N_2_ adsorption–desorption isotherms of the four PY‐COF‐COOH@MOF‐X composites reveal hierarchical porosity, evidenced by multimodal pore size distributions (Figures S48–S55). The evolution of their specific surface areas with precursor concentration (X) unveils a complex, non‐additive relationship governed by the relative dimensions and morphology of the constituent COF and MOF (Figure 5a and Tables S3–S6). At low X values (0.10/0.25), insufficient molecular concentrations impede extensive MOF crystallization, resulting in specific surface areas dominated by the PY‐COF framework. As X increases to 0.50/1.00, MOF growth on the COF surface approaches or reaches saturation, and the composite's specific surface area emerges from a dynamic interplay between the individual specific surface areas and spatial constraints imposed by the heterostructure. This mechanistic insight, which we term the aperture synergistic regulation (ASR) effect, rationalizes the seemingly contradictory specific surface area trends reported for COF@MOF hybrids in prior studies [16, 34].

To validate ASR in separation performance, we measured single‑component isotherms of CH_4_, C_2_H_6,_ and C_3_H_8_ (298 K) on PY‑COF‐COOH@ZIF‑X (X = 0.10–1.00) (Figure 5c and Figure S56). PY‑COF and ZIF‑8 exhibit near‑linear uptake for CH_4_/C_2_H_6_, consistent with weak host‐guest interactions. For PY‑COF‐COOH@ZIF‑X at low X (0.10–0.50), sub‑saturation ZIF‑8 growth preserves the mesoporous COF/MOF duality, yielding adsorption profiles similar to the parents. At X = 1.00, saturation coverage enhances affinity for C_2_H_6_/C_3_H_8_, increasing low‑pressure uptakes (e.g., C_2_H_6_ at 10 kPa and C_3_H_8_ at 5 kPa) and leading to markedly higher capacities at saturation. To elucidate the thermodynamic driving force for the observed adsorption behavior, we further measured the CH_4_, C_2_H_6_, and C_3_H_8_ adsorption isotherms at 273 K (Figure 5d and Figure S57) and calculated the isosteric heats of adsorption (Q_st_) using the Virial equation based on the 273 and 298 K isotherms (Figure 5e and Figure S58). As expected, all adsorbents exhibited higher uptake capacities at 273 K than at 298 K, consistent with the exothermic nature of physisorption. The Q_st_ values for C_3_H_8_ were consistently the highest among the three gases across all materials, confirming the strongest host–guest interaction with propane. For PY‐COF, the Q_st_ ​of C_3_H_8_ decreased gradually with increasing loading, indicating energetic heterogeneity of adsorption sites. In contrast, ZIF‐8 displayed relatively flat Q_st_ profiles, suggesting more uniform binding environments. Notably, PY‐COF‐COOH@ZIF‐1.00 exhibited elevated Q_st_ values for C_2_H_6_ and C_3_H_8_ compared to its parents, reflecting enhanced affinity arising from the synergistic interface between the COF shell and ZIF‐8 core. In addition, the Q_st_ values for CH_4_ remained low and nearly constant over the examined loading range, indicating comparatively weak host–guest interactions and a lower affinity of CH_4_ relative to C_2_H_6_ and C_3_H_8_. To more precisely evaluate the separation performance of the PY‐COF‐COOH@ZIF‐X under mixed‐gas conditions, we calculated IAST selectivity of C_3_H_8_/CH_4_ (5:85, v/v) and C_2_H_6_/CH_4_ (10:85, v/v) at room temperature (298 K). As shown in Figure 5f,g and Table S7, IAST analysis at 100 kPa shows selectivity of 55.49 for C_2_H_6_/CH_4_ (10/85) and 273.86 for C_3_H_8_/CH_4_ (5/85) on PY‑COF‐COOH@ZIF‑1.00, representing 7.63 times and 5.46 times improvements over ZIF‑8.

To evaluate the dynamic separation performance, breakthrough experiments using a CH_4_/C_2_H_6_/C_3_H_8_ ternary mixture (5/10/85, V/V/V) were conducted at 298 K and 1 bar on PY‐COF, ZIF‐8, and PY‑COF‐COOH@ZIF‑X (X = 0.10–1.00) composites. In all cases CH_4_ elutes first and quickly reaches C/C_0_ = 1.0 (Figure 5h and Figure S59), indicating negligible retention, which is consistent with the 298 K static single‑component isotherms. The delayed breakthroughs of C_2_H_6_ and C_3_H_8_ reflect stronger interactions with the frameworks, and the retention order is C_3_H_8_> C_2_H_6_> CH_4_. Among the samples, PY‑COF‑COOH@ZIF‑1.00 shows the longest retention for C_3_H_8_ (breakthrough time 102 min), corresponding to a dynamic uptake of 79.4 cm^3^·g^−1^, in good agreement with the 298 K static adsorption results. In contrast, PY‑COF‑COOH@ZIF‑0.50 exhibits the fastest overall elution: the three components are sequentially washed out within 20 min, indicating rapid separation dynamics. Pristine PY‑COF and ZIF‑8 show intermediate retention behavior; C_3_H_8_ breakthrough times are 28 and 40 min, corresponding to dynamic uptakes of 21.8 and 31.1 cm^3^·g^−1^, respectively. Based on the above results, we systematically compared the PY‑COF‑COOH@ZIF‑X fabricated via the MPF strategy with recently reported ternary‑alkane separation materials (Table S8) [48, 49, 50, 51, 52, 53, 54, 55, 56, 57, 58, 59]. The results demonstrate that this material outperforms most known counterparts in both IAST selectivity and adsorption capacity, highlighting the advantage of the MPF strategy in constructing hierarchical‑pore COF@MOF composites.

To further quantify structure–performance relationships, we performed Pearson correlations among S_BET_, pore volume, average pore size, and key metrics (static/dynamic uptake and IAST selectivity; Figure 5i). The analysis shows that S_BET_ correlates positively with hydrocarbon uptake (e.g., *r* = 0.80 vs. static C_3_H_8_; *r* = 0.75 vs. dynamic C_2_H_6_), average pore size correlates negatively with uptake (e.g., *r* = −0.83 vs. static C_3_H_8_; *r* = −0.79 vs. dynamic C_2_H_6_), and pore volume exhibits only weak correlations (e.g., *r* = 0.21 vs. static C_3_H_8_). These quantitative results indicate that ASR controls separation performance mainly through the synergistic tuning of surface area (total uptake) and pore size (selectivity) rather than via single‐parameter changes. Mechanistically, we propose that PY‑COF acts as a structural modulator and nucleation scaffold for ZIF‑8 growth, yielding an effective pore environment, underpinned by ASR and IDGS effects, which favors alkane retention and discrimination. Because performance depends on this balance, optimal loading (X) is required to maximize pore tuning while preserving accessible adsorption sites.

We next systematically evaluated the enrofloxacin (ENR) degradation performance of PY‐COF‐COOH@Co‐MOF‐X (X = 0.10, 0.25, 0.50, 1.00) composites through Fenton‐like reactions (Figure 5j and Figure S60). Remarkably, all PY‐COF‐COOH@Co‐MOF‐X (0.20 g L^−1^) composites achieved near‐complete ENR elimination (97.55%–99.05% removal rate) within 30 min, exhibiting 2.78–2.82‐fold enhancement in catalytic efficiency compared to pristine Co‐MOF (35.12% removal rate). While PY‐COF alone exhibited no H_2_O_2_ activation capability, it demonstrated superior ENR adsorption capacity (61.83 mg g^−1^). The synergistic enhancement arises from defect‐rich Co‐MOF providing abundant active sites coupled with PY‐COF's preconcentration capability, which optimizes H_2_O_2_ utilization efficiency. Notably, the catalytic performance showed minimal dependence on composite ratios (X = 0.10–1.00), indicating robust structural integration and efficient interfacial electron transfer within the hybrid system.

Kinetic analysis revealed that the catalytic rate constants (K_obs_) of PY‑COF‐COOH@Co‑MOF‑X (0.20 g L^−1^) during the Fenton‐like reaction (Figure 5k and Table S9) ranged from 0.1581 to 0.2062 min^−1^ in the 0–5 min interval, exceeding that of Co‑MOF (0.0405 min^−1^) due to defect sites that enhance ENR adsorption, achieving a maximum improvement of 5.09 times. Remarkably, despite negligible ENR adsorption, PY‑COF@Co‑MOF‑1.00 exhibited a K_obs_ of 0.2062 min^−1^, indicating that defect‑type Co‑MOF generated via the IDGS effect displays superior H_2_O_2_ selectivity relative to pristine Co‑MOF. In addition, in contrast to 0–5 min, both PY‐COF‐COOH@Co‐MOF‐0.10 and PY‐COF‐COOH@Co‐MOF‐1.00 showed a significant decrease in K_obs_ from 5–10 min. This reduction is attributed mainly to the lower content of long‐range ordered defect Co‐MOFs in PY‐COF‐COOH@Co‐MOF‐0.10 and the insufficient adsorption performance of PY‐COF‐COOH@Co‐MOF‐1.00 for ENR, leading to decreased efficiency in H_2_O_2_ utilization. Additionally, PY‐COF‐COOH@Co‐MOF‐0.25 and PY‐COF‐COOH@Co‐MOF‐0.50 maintained stable K_obs_ values at both 0–5 min and 5–10 min, which may be related to the stability of the catalysts. The structural stability of PY‐COF‐COOH@Co‐MOF‐X (X = 0.10–1.00) was verified through the metal ion leakage test conducted after the end of the Fenton‐like reaction, where Co leaching rates for PY‐COF‐COOH@Co‐MOF‐X (X = 0.50/1.00) were>70% lower than that of pure Co‐MOF (Figure 5l).

Subsequently, the cyclic stability of PY‐COF‐COOH@Co‐MOF‐X (X = 0.10–1.00) composites was evaluated through seven consecutive Fenton‐like reaction cycles for ENR degradation, with Co ion leakage monitored after each cycle. As shown in Figure S61, the initial removal rates of PY‑COF‑COOH@Co‑MOF‑X were 90.60%–100%, but distinct stability trends emerged with increasing cycle numbers. Specifically, PY‐COF‐COOH@Co‐MOF‐0.10 and PY‐COF‐COOH@Co‐MOF‐0.25 gradually declined from 90.60% and 90.76% to 60% and 43%, respectively, with Co leaching fluctuating at 3.2%–4.0%. In contrast, the X = 0.50 sample exhibited the best stability, maintaining near‐complete removal for six cycles and 60.05% at the seventh, while Co leakage remained low (only 3.85% at cycle 7). Conversely, the X = 1.00 sample showed low initial leaching (3.12%) but a sharp increase to ∼7.43% after cycle 5, coinciding with the loss of removal efficiency. Based on the above results, a statistical comparison of recent reports on ENR degradation via AOPs over the past three years was conducted (Table S10). The results demonstrate that the PY‐COF‐COOH@Co‐MOF‐X synthesized via MPF strategy exhibits superior specific activity (*r* = 0.3437 mg·g^−1^·s^−1^) and cyclic stability compared to most reported catalysts [60, 61, 62, 63, 64, 65, 66]. These results indicate that the synergistic effect between the adsorption module (PY‐COF) and the catalytic module (Co‐MOF) at an appropriate ratio is a prerequisite for efficient water purification and simultaneously enhances the catalyst's structural stability.

Finally, the enhanced catalytic mechanism exhibited by PY‐COF@Co‐MOF‐X was investigated. With Radical quenching (Figure S62) and EPR studies (Figure 5m–o) identified •OH and O_2_^•−^ as the dominant reactive species, while ^1^O_2_ was detected as a secondary product, formed via radical coupling [67]. Furthermore, the continuously enhanced signal strength of radical detected over a period of 30 s to 10 min indicates that PY‐COF‐COOH@Co‐MOF‐X possesses rapid and sustained H_2_O_2_ activation properties, which can prolong the supply time of free radicals and thereby enhance the efficiency of ENR removal. To further clarify the origin of the enhanced catalysis, we performed DFT calculations on three models (isolated Co‑MOF, PY‑COF‐COOH@Co‑MOF, and defective PY‑COF‐COOH@Co‑MOF) (Figure S63) and computed the free‑energy step diagram for H_2_O_2_ activation toward O_2_^•‑^ formation (Figure 5p). The calculations indicate that, relative to the isolated Co‑MOF (0.21 and 0.25 eV), the PY‑COF‐COOH@Co‑MOF interface lowers the free‑energy barriers for HO_2_^‑^ (0.16 eV) formation and the subsequent step to O_2_^•‑^ (−0.40 eV), and the defective PY‑COF‐COOH@Co‑MOF further reduces these barriers (−0.01 and −0.64 eV). These energetic trends demonstrate that the interfacial configuration and defect sites facilitate H_2_O_2_ activation and promote O_2_^•‑^ generation. The DFT results are consistent with our EPR and radical‑quenching observations of sustained and enhanced radical signals, and together they substantiate the mechanism that PY‑COF coupled with (defective) Co‑MOF creates a highly active microenvironment for continuous ROS production and efficient contaminant degradation.

Collectively, integrating PY‑COF‐COOH with structurally and compositionally tailored MOFs via the MPF strategy yields COF@MOF heterostructures with programmable hierarchical porosity, interface chemistry, and defect structure. For PY‑COF‐COOH@ZIF‑X, ASR effect reconciles the non‑additive evolution of surface area with MOF loading and directly translates into sharpened size‑ and affinity‑based discrimination among light hydrocarbons, delivering C_2_H_6_/CH_4_ and C_3_H_8_/CH_4_ selectivity that outperform benchmark sorbents for natural‑gas upgrading. In parallel, PY‑COF‐COOH@Co‑MOF‑X exploits defect‑engineered Co‑MOF domains and COF‑mediated preconcentration to decouple adsorption and activation functions, affording fast, nearly quantitative ENR degradation over a broad composition window, with suppressed metal leaching and sustained radical generation. These results delineate clear structure–property–performance correlations and highlight MPF‑derived hierarchical COF@MOF interfaces as a general platform for precision molecular separations and robust heterogeneous advanced oxidation.

## 3 Conclusion

In summary, we have established an MPF strategy, enabled by carboxylate engineering, for the precision synthesis of COF@MOF hybrids. This approach leverages universal metal‐anchoring sites on the PY‐COF‐COOH surface to direct MOF crystallization through IDGS or/and ASR effect, granting programmable control over morphology, porosity, and coordination defects. Among these, we acknowledge that the CFS effect, while supported by systematic PXRD analysis and thermodynamic calculations, still requires further experimental validation to be fully elucidated and to contribute to a more comprehensive design framework. The validity of this integrative framework is further substantiated by the exceptional performance of the resulting materials: the hierarchically porous PY‐COF‐COOH@ZIF‐1.00 in CH_4_/C_2_H_6_/C_3_H_8_ separation and the defect‐engineered PY‐COF‐COOH@Co‐MOF‐X in H_2_O_2_ activation. Beyond demonstrating a versatile pathway to overcome synthesis incompatibilities, this work provides a foundational design framework for the next generation of multifunctional hierarchical materials.

## Author Contributions

**Ying Zhao**: conceptualization, investigation, writing – original draft, visualization, methodology, validation, writing – review and editing, software, formal analysis, data curation. **Dongmei Chen**: conceptualization, funding acquisition, writing – review and editing, project administration, resources, supervision, formal analysis. **Dingtang Li**: visualization. **Huilin Zheng**: methodology, investigation. **Shuyu Xie**: conceptualization, funding acquisition, writing – review and editing, project administration, formal analysis, resources, supervision. **Min Chen**: formal analysis, writing – review and editing.

## Conflicts of Interest

The authors declare no conflicts of interest.

## Supporting information




**Supporting File**: advs76204‐sup‐0001‐SuppMat.pdf.

## Data Availability

The data that support the findings of this study are available in the supplementary material of this article.
