# Supplementary material for: Carboxylate‐Driven Metal Pre‑Fixation COF@MOF Synthesis: Enabling Tailored Structural, Dimensional, and Defect Engineering
Source: Adv Sci (Weinh). 2026 Jun 18:e76204. Online ahead of print. doi: 10.1002/advs.76204 (PMC13336427; doi:10.1002/advs.76204)
Supplement: Supplementary file 1 — Supporting File: advs76204‐sup‐0001‐SuppMat.pdf. [file ADVS-9999-e76204-s001.pdf]

Supporting Information for

Carboxylate-Driven Metal Pre-Fixation COF@MOF Synthesis: Enabling Tailored Structural, Dimensional, and Defect Engineering

Ying Zhao[1,2†], Dongmei Chen[2†], Huilin Zheng[2], Min Chen[2], Dingtang Li[2], Shuyu Xie[1,2,3,4,5*]

[a]State Key Laboratory of Agricultural Microbiology Core Facility, Huazhong Agricultural University, Wuhan, Hubei 430070, China.

[b]National Reference Laboratory of Veterinary Drug Residues (HZAU), Huazhong Agricultural University, Wuhan, Hubei 430070, China.

[c]Hubei Hongshan Laboratory, Wuhan, Hubei 430070, China.

[d]Key Laboratory of Prevention & Control for African Swine Fever and Other Major Pig Diseases, Ministry of Agriculture and Rural Affairs, Wuhan, Hubei 430070, China.

[e]Frontier Science Center for Animal Breeding and Healthy Husbandry, Ministry of Education, Wuhan, Hubei 430070, China.

Supporting information for this article is given via a link at the end of the document.

Note: [†]These authors contributed equally to this work.

*Corresponding author: Shuyu Xie.
Email: Xieshuyu@mail.hzau.edu.cn

This PDF file includes:

Supporting text
Figures S1 to S63
Tables S1 to S10

S1. Materials

4-fluorobenzoic acid (4-FA, AR), Dihydrate and zinc acetate ($Zn(CH_3COO)_2·2H_2O$, AR), 2-Methylimidazole (2-MI, AR), Ferric chloride hexahydrate ($FeCl_3·6H_2O$, AR), Cobalt hexahydrate acetate (Co $(CH_3COO)_2·6H_2O$, AR), Zirconium dichloride octahydrate ($ZrOCl_2·8H_2O$, AR), 2-amino-terephthalic acid (NH_2-BDC, AR) were purchased from Sinopharm Group of China. 1,4-dihydroxybenzidine (AR), 1,3,6,8-tetra (4-formylphenyl) pyrene (AR) were purchased from Aladdin.

S2. Methods

S2.1. *Characterization*

SEM images were obtained using a HITACHI S-4800 microscope. HR-TEM and elemental mapping images were acquired with a FEI Tecnai G2 F20 transmission electron microscope. Powder-XRD (PXRD) patterns were recorded using Bruke D8 ADVANCE X-ray powder diffractometer, using Cu Kα radiation with a wavelength of 0.15406 nm. ^{13}C Solid-State NMR was conducted using a JNM-ECZ600R spectrometer, operating at a resonance frequency of 150.913 MHz for ^{13}C. And the rotor diameter was 3.2 mm, the testing method employed was CPMAS (Cross-Polarization Magic Angle Spinning) at a MAS frequency of 12 kHz. A relaxation delay of 2 seconds was implemented, and a total of 2000 scans were performed to ensure accurate and reliable results. Raman spectra were collected on Horiba LabRAM HR Evolution. X-ray absorption fine structure spectroscopy (XAFS) characteristic was conducted at the beamline 1W1B of the Beijing Synchrotron Radiation Facility (BSRF), Institute of High Energy Physics (IHEP), Chinese Academy of Sciences (CAS). For pair-distribution function (PDF) characteristic, Total scattering measurements were performed at beamline ID11 at the European Synchrotron Radiation Facility (ESRF). The sample powders were loaded into cylindrical slots (approx. 1 mm thickness) held between Kapton windows in a high-throughput sample holder. Each sample was measured in transmission geometry with an incident X-ray energy of 90.65keV. Data were collected with the Eiger2X CdTe 4M hybrid photon-counting pixel detector. The sample-to-detector distance was approximately 0.3 m for the total scattering measurements. Background measurements for the empty windows were measured and subtracted. NIST SRM 660b (LaB_6) was used for geometry calibration performed with the software pyFAI followed by image integration including a flat-field, geometry, solid-angle, and polarization corrections.

S2.2. Synthesis

S2.2.1. Synthesis of PY-COF [1]

An n-butyl alcohol (n-BuOH)/o-dichlorobenzene (o-DCB)/6 M AcOH (5/5/1 by vol., 1.1mL) mixture of 1,4-dihydroxybenzidine (0.04 mmol, 8.6 mg) and 1,3,6,8-tetra(4-formylphenyl) pyrene (0.02 mmol, 12.3 mg) in a Pyrex tube (10mL) was degassed by three freeze-pump-thaw cycles. The tube was sealed off and heated at 120°C for 3 days. The precipitate was collected by centrifugation and soaked in DMF for two days, then washed with anhydrous THF 5 times and acetone twice. The powder was dried at 120°C under vacuum overnight.

S2.2.2. Synthesis of PY-COF-COOH [2]

100 mg prepared PY-COF was added to 40 mL DMF solution containing 1.00 mmol (219.00 mg) of 4-FA and 1.5 mmol (207.30 mg) of K_2CO_3. Then, the mixture was stirred (200 rpm.) at room temperature for 30 minutes to ensure complete dissolution. Subsequently, the reactants were transferred to 110 ℃ and stirred under a nitrogen atmosphere for 72 hours at 200rpm. When reaction complete, the unreacted reactants are removed successively with deionized water, DMF and anhydrous ethanol. The obtained solid is vacuum-dried at 80℃ for 24 hours and then stored at 4℃ for future use.

S2.2.3. Synthesis of single ZIF-8, Fe-MOF, UIO-66 and Co-MOF [3,4]

ZIF-8**:** In a typical synthesis, 1.00 mmol $Zn(CH_3COO)_2·2H_2O$ and 8.00 mmol 2-MI were respectively dissolved in 15 mL DMF and ultrasonically treated for 5 minutes to ensure complete dissolution. Subsequently, the two clear solutions were transferred to 50 mL solvent bottles and ultrasonically treated for 5 minutes to mix evenly. Then mixed solution were heated at 120 ℃ for 24 hours. When

reaction completed, naturally cooled to room temperature. The precipitate is collected by centrifugation and washed three times each in sequence with DMF and anhydrous ethanol, and then vacuum-dried at 80℃ for 24 hours.

Fe-MOF, UIO-66 and Co-MOF**:** Except the molar ratio of metal ions and organic ligands was controlled at 1:1, the remaining steps were consistent with ZIF-8.

S2.2.4. Synthesis of PY-COF-COOH@M-MOF-X (M=Zn/Fe/Zr /Co, X=0.10/0.25/0.50/1.00)

PY-COF-COOH@ZIF-X: 100 mg finely grounded PY-COF-COOH was added to a 50 mL flask containing 15 mL of DMF solution and subjected to ultrasonic treatment for 10 minutes to ensure complete dispersion. Subsequently, X (X = 0.10, 0.25, 0.50, or 1.00) mmol of $Zn(CH_3COO)_2{\cdot}2H_2O$ was added to the PY-COF-COOH solution and stirred to achieve a homogeneous mixture. Then, 15 mL DMF containing 8X mmol of 2-MI was added to the flask, and the mixture was heated at 120°C for 24 hours. After the reaction, the product was cooled naturally to room temperature. It was then washed using the same procedure as for ZIF-8 and vacuum-dried at 80°C overnight, followed by storage at 4℃ for future use.

PY-COF-COOH@Fe-MOF-X, PY-COF-COOH@UIO-X and PY-COF-COOH@Co-MOF-X: Except the molar ratio of metal ions and organic ligands was controlled at 1:1, the remaining steps were consistent with PY-COF-COOH@ZIF-X.

S2.3. Gas separation test

Before the adsorption isotherms test, the samples were heated at 353K for 24 h. After that, PY-COF, ZIF-8 and PY-COF-COOH@ZIF-X (X=0.10, 0.25, 0.50 and 1.00) were evacuated at 353 K for 24 h until the pressure was below 10 μm Hg. Gas adsorption measurements were conducted on a BELSORP max II surface area analyzer and a BSD660 instrument. The sorption isotherms were collected at 298 K/273 K on activated samples.

S2.3.1 Fitting of Pure Component Isotherms

The pure component isotherm data of PY-COF-COOH@ZIF-X (X=0.10, 0.25, 0.50 and 1.00) for CH_4, C_2H_6 and C_3H_8 were fitted with the dual-site Langmuir-Freundlich (DSLF) equation.

$$q = q_{A,sat}\,\frac{b_A P^{\nu A}}{1 + b_A P^{\nu A}} + q_{B,sat}\,\frac{b_B P^{\nu B}}{1 + b_B P^{\nu B}}$$

Here, q is the gas uptake per mass of adsorbent (in mmol/g). *P* is the pressure of the bulk gas at equilibrium with the adsorption phase (in kPa). $q_{A,sat}$ and $q_{B,sat}$ are the saturation uptakes for sites 1 and 2 (in mmol/g). b_A and b_B are the affinity coefficients of sites 1 and 2 (in kPa^{-1}). v_A and v_B are the deviations from an ideal homogeneous surface. The parameters obtained from fitting of single-component adsorption isotherms are provided in Table S7.

S2.3.2 IAST calculation of adsorption selectivity [5]

The adsorption selectivity was established from the Ideal Adsorbed Solution Theory (IAST) of PY-COF-COOH@ZIF-X (X=0.10, 0.25, 0.50 and 1.00) for C_2H_6/CH_4 or C_3H_8/CH_4 mixtures. The adsorption selectivity, Sads, is defined by the following equation:

$$S_{ads} = \frac{q_1/q_2}{p_1/p_2}$$

where q_1 and q_2 are the molar loadings in the adsorbed phase in equilibrium with the bulk gas phase, and p_1 and p_2 are partial pressures. The isotherm fitting parameters and the partial pressure of each component were used as input parameters to calculate the IAST selectivity. The solution of the equation was done by the IAST++ software.

S2.3.3 Calculation of the Isosteric Heat of Adsorption (Qst).

Before the calculation of Q_{st}, the isotherms tested at 273 K and 298 K were fitted by the Virial equation comprising of the temperature-independent parameters a_i and b_j. The Virial equation is defined as:

$$\ln(p) = \ln(n) + (\frac{1}{n})\sum_{i=0}^{m} a_i n^i + \sum_{j=0}^{n} b_j n^j$$

Here, p presents pressure, n presents the amount adsorbed, T is temperature, a_i and b_j are temperature-independent empirical parameters, and m, n represent the number of coefficients required to adequately describe the isotherms. The values of the Virial coefficients a_0 to a_m were then used to calculate the Qst using the following expression:

$$Q_{st} = -R\sum_{i=0}^{m} a_i n^i$$

Q_{st} is the coverage-dependent isosteric heat of adsorption and R is the universal gas constant. The heat enthalpy of C_3H_8, C_2H_6, and CH_4 for PY-COF, ZIF-8 and PY-COF-COOH@ZIF-X (X=0.10~1.00) are determined by using the sorption data in the pressure range from 0-100 kPa (at 273 K to 298 K).

S2.3.4 Breakthrough Test.

In a typical breakthrough test (Schem S1), 2.00±0.01 g of PY-COF, ZIF-8 or PY-COF-COOH@ZIF-X was packed as a fixed bed into an adsorption column (Ø 0.50 cm × 50 cm) with silica wool filling the voids; after activation the bed was purged in situ with He at 20 mL·min^{-1} until the GC (GC-9860) baseline was stable and free of impurity peaks. Target feed conditions were then set by a mass flow controller (MFC); once the feed was stabilized the gas stream was switched to the mixed feed (C_3H_8/C_2H_6//CH_4, 85/10/5, 2 mL/min) and the chromatographic system continuously recorded the outlet concentration profiles of each component over time. After saturation, desorption/regeneration was performed by heating the column under a constant N_2 or Ar flow.

The amountof gas adsorbed i (q_i) is calculated from the breakthrough curves using the following:

$$q_i = \frac{V_t P_i \Delta T}{m}$$

Here, V_t is the total flow rate of gas (mL/min), P_i is the partial pressure of gas i (atm), ΔT is the time for initial breakthrough of gas i tooccur (mins) and m is the mass of the sorbent (g).

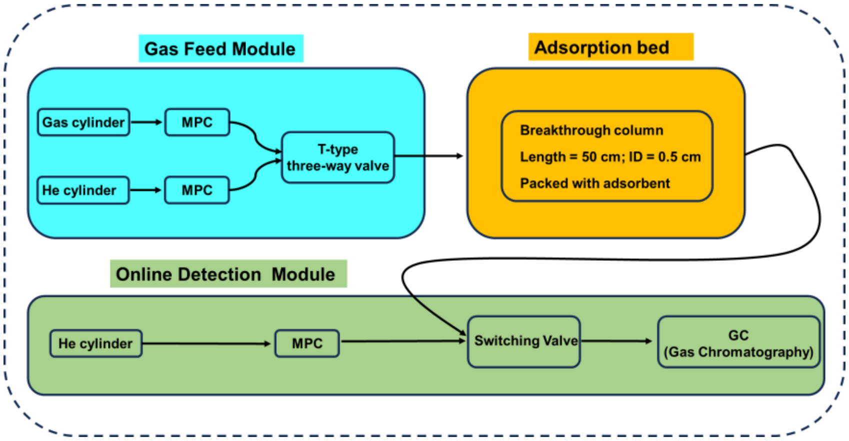

Schem S1. Schematic diagram of the Breakthrough test device

S2.4. Fenton oxidation degradation test

Enrofloxacin (ENR) was used as the target pollutant to evaluate the adsorption and Fenton oxidation performance of PY-COF-COOH@Co-MOF-X (X=0.10, 0.25, 0.50 and 1.00). First, a well-dispersed catalyst with a concentration of 0.1 g/L was placed in 100 mL of an ENR aqueous solution (20 mg/L). The mixture was oscillated at 25 °C and 180 r/min for 1 hour to reach the adsorption-desorption equilibrium. Then, 0.3 mL of 30% H_2O_2 was added to initiate the catalytic reaction. At the time points of 0 and 60 min during the adsorption process, and 5, 10, 20, 30, 45, and 60 min

during the Fenton reaction process, 1.00 mL of the reaction solution was taken into 1.00 mL of methanol to quench the free radicals. Subsequently, the solution was filtered through a 0.22 μm membrane and transferred to a liquid-phase vial.

S2.4.1 Free radical quenching experiment

Tertiary butanol (TBA) was used as the quencher of •OH, ethyl alcohol (EA) was introduced to the solution as a scavenger of •OH and $SO_4^{\bullet-}$, furfuryl alcohol (FFA) and p-benzoquinone (p-BQ) were used to probe the formation of $^1O_2$ and $O_2^{\bullet-}$.

S2.4.2 Kobs calculation

$$\ln\frac{C_t}{C_0} = -K_{obs}t$$

where C_0 and C_t denotes the concentration of ENR at the initial internal and at time t, respectively. Kobs represents the pseudo-first order constant.

S2.5. DFT software, modules and functions

Density Functional Theory (DFT) calculations were performed by using the CP2K package [6]. By using the CP2K package mixed Gaussian and plane-wave scheme [7] and the Quickstep module [8]. The Perdew-Burke-Ernzerhof (PBE) exchange correlation functional [9], Goedecker-Teter-Hutter (GTH) pseudopotential [10], DZVP-MOLOPT-SR-GTH / TZVP-MOLOPT-SR-GTH / TZVP-MOLOPT-GTH basis sets were used to describe the system [8]. A plane-wave energy cut-off and relative cut-off of 400 Ry and 55 Ry have been employed, respectively. The energy convergence criterion was set to 10^{-6} Hartree. The DFT-D3(BJ) level correction for dispersion interactions was applied [11]. Structural optimization was performed using the Limited Memory Broyden-Fletcher-Goldfarb-Shannon (LBFGS) optimizer, until the maximum force is below 0.00045 Ry/Bohr (0.011 eV/Å). The charge differential density was calculated and plotted through the software Multiwfn [12] and Visual Molecular Dynamics (VMD) [13], respectively. The Gibbs free-energy diagrams were estimated under zero potential (U = 0) by the equation [14]:

$$\Delta G_H = \Delta E_H + \Delta ZPE - T\Delta S$$

during which ΔE_H is the energy change between the reactant and product obtained from DFT calculations, ΔZPE is the change of zero-point energy and T and ΔS represents the temperature and change of entropy, respectively. T=298.15 K was employed in this case.

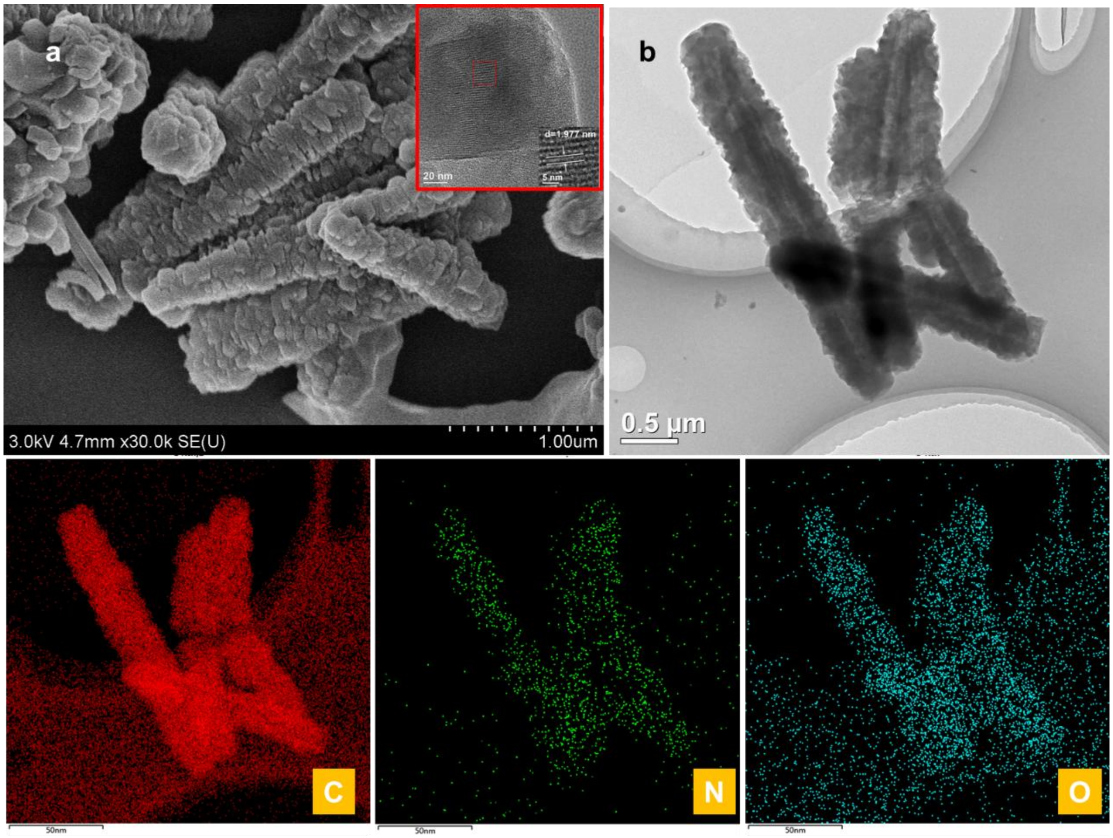

Fig. S1 SEM (a), HR-TEM (b) and element mapping of PY-COF

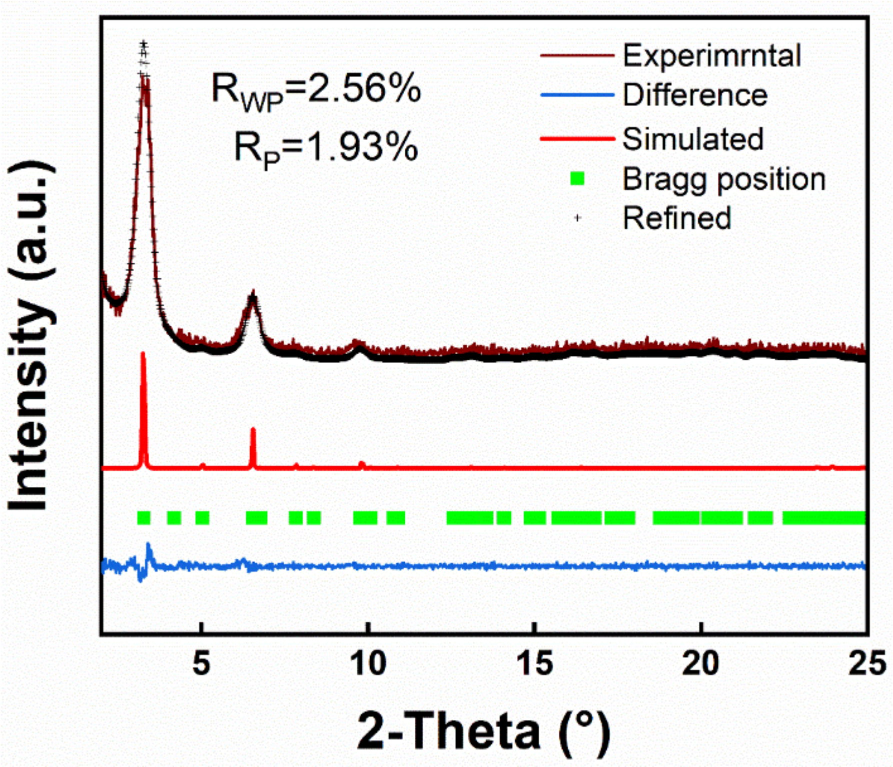

Fig. S2. Experimental and simulated PXRD patterns of PY-COF

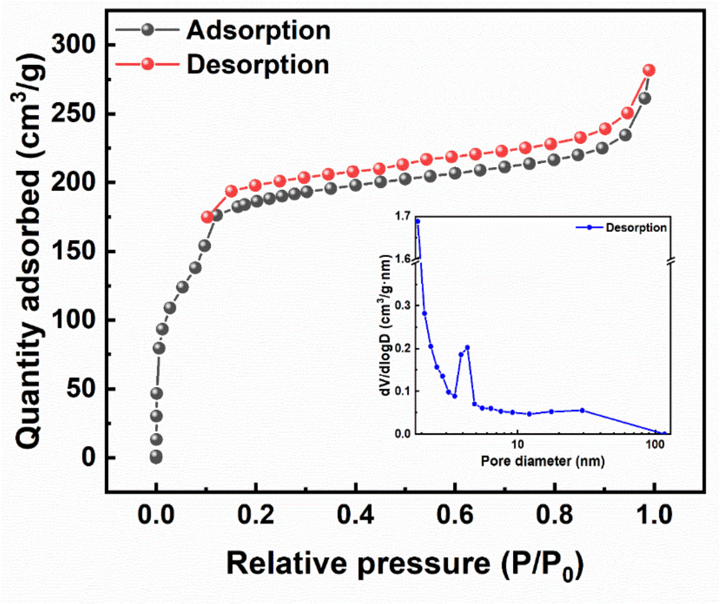

Fig. S3 N_2 adsorption-desorption isotherms of PY-COF

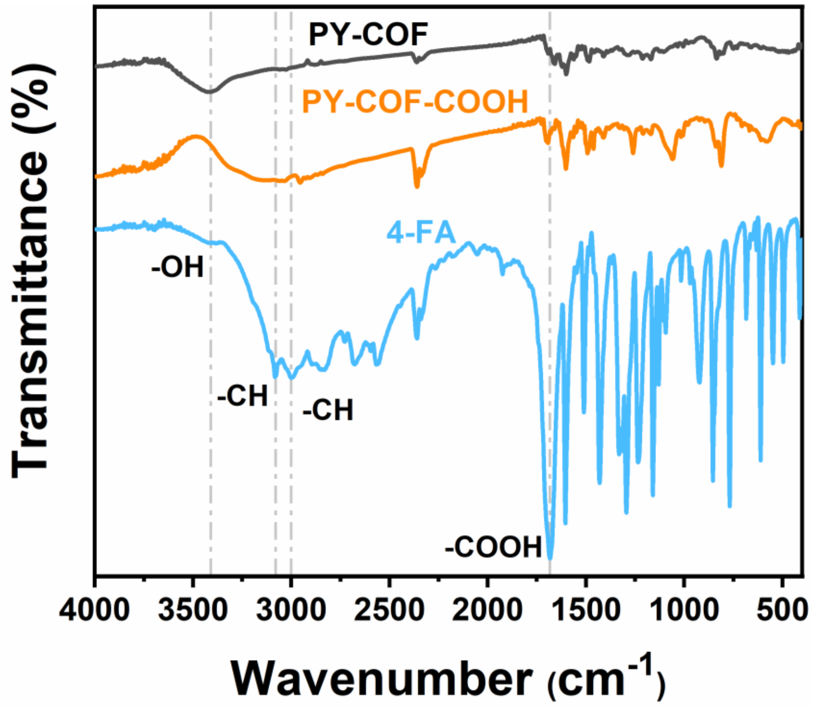

Fig. S4 FT-IR characteristic of PY-COF-COOH

[Note] At 3414 cm^{-1}, the broad peak corresponding to the phenolic hydroxyl group in PY-COF disappears after the substitution reaction, while a vibrational peak for the carboxyl group appears at 1686 cm^{-1}, indicating that the carboxyl group has been successfully modified onto the surface of PY-COF.

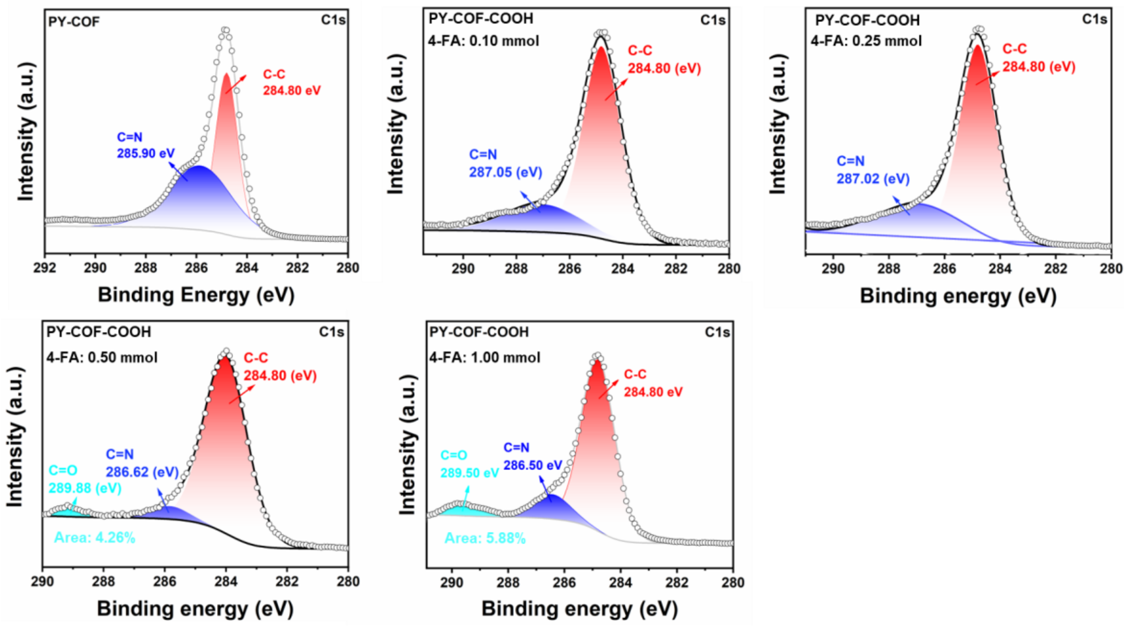

Fig. S5 The C1s detailed spectrum of PY-COF and the PY-COF-COOH synthesized with different 4-FA added at different molar masses

[Note] The peak at 285.90 eV is attributed to the C=N bond in the structure of PY-COF. After carboxyl modification, the C=N peak shifted to a higher binding energy by 0.60 eV, and a characteristic peak for C=O appeared at 289.50 eV, further confirmed the successful modification of the carboxyl group.

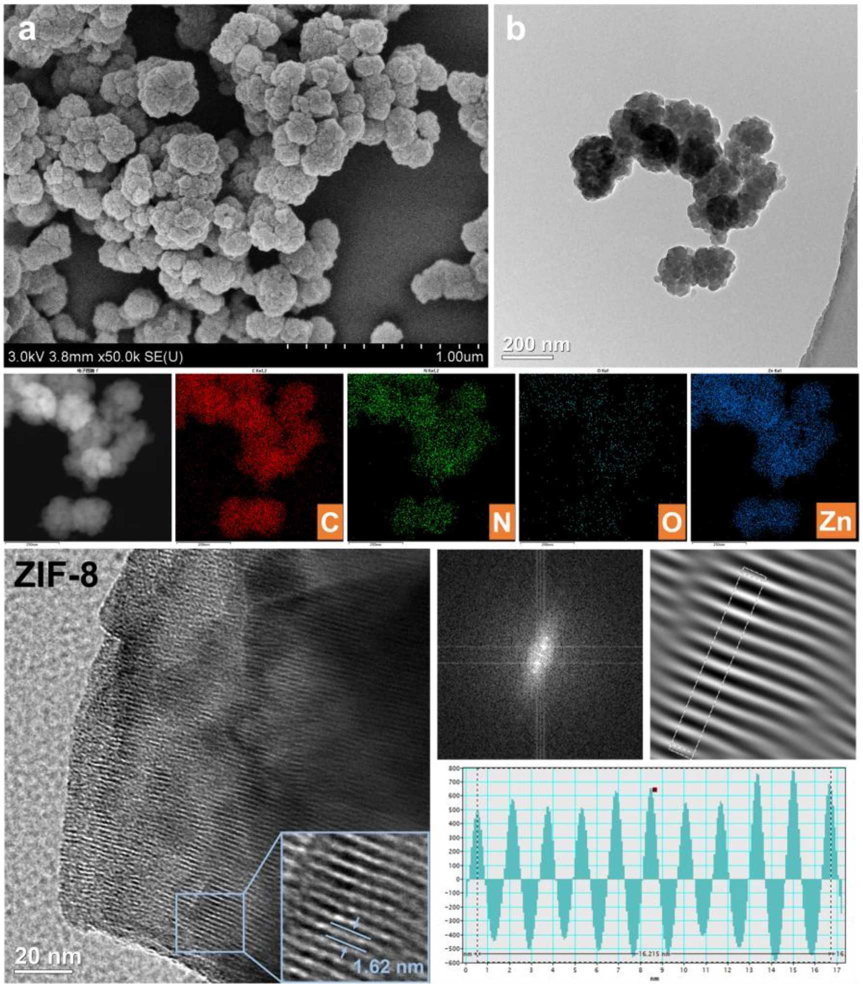

Fig. S6 SEM (a), HR-TEM (b) and element mapping of ZIF-8

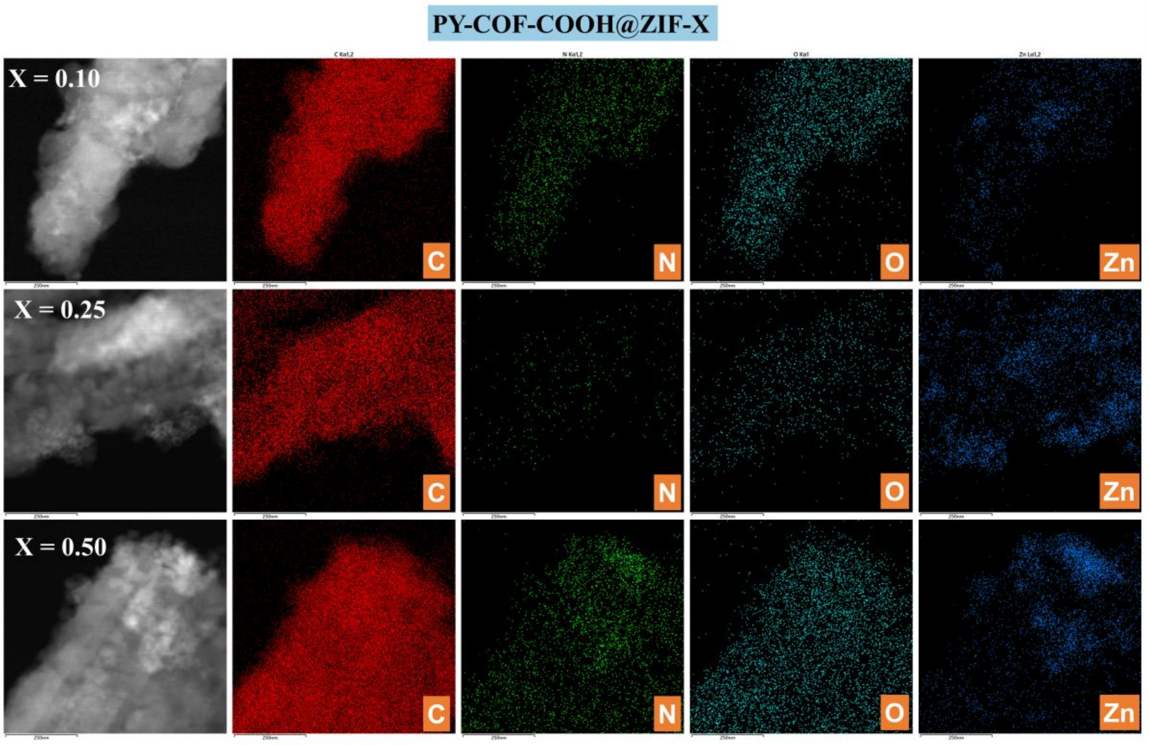

Fig. S7 Element mapping of PY-COF-COOH@ZIF-X, X=0.10, 0.25 and 0.50

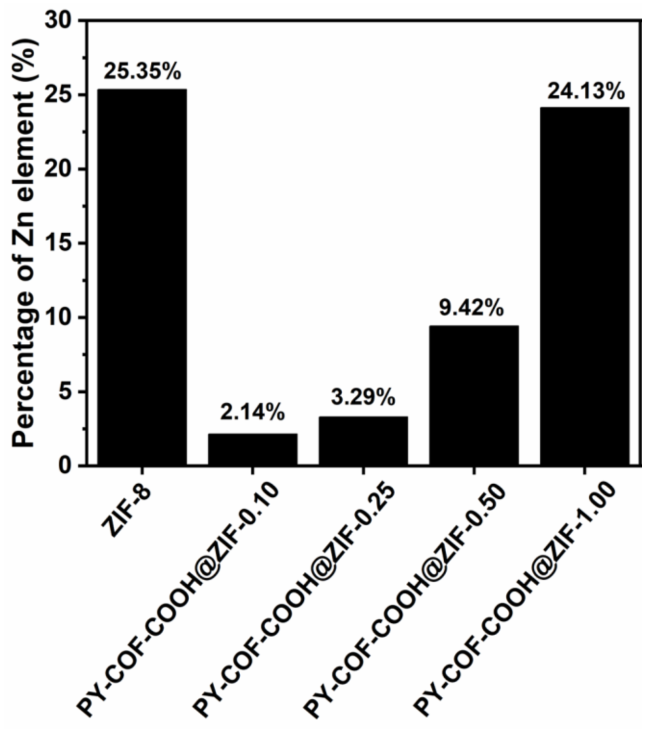

Fig. S8 Element mapping of PY-COF-COOH@ZIF-X, X=0.10, 0.25 and 0.50. [Sample pretreatment conditions]: After complete acid digestion of 10 mg of the material, the Zn content was determined.

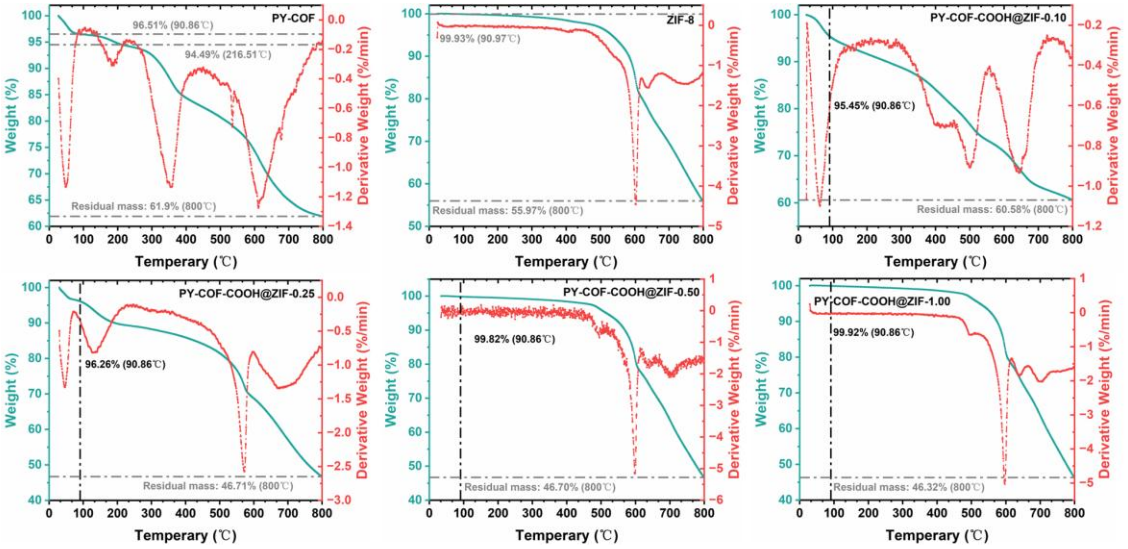

Fig. S9 The TGA curves of PY-COF, ZIF-8 and PY-COF-COOH@ZIF-X (X=0.10~1.00)

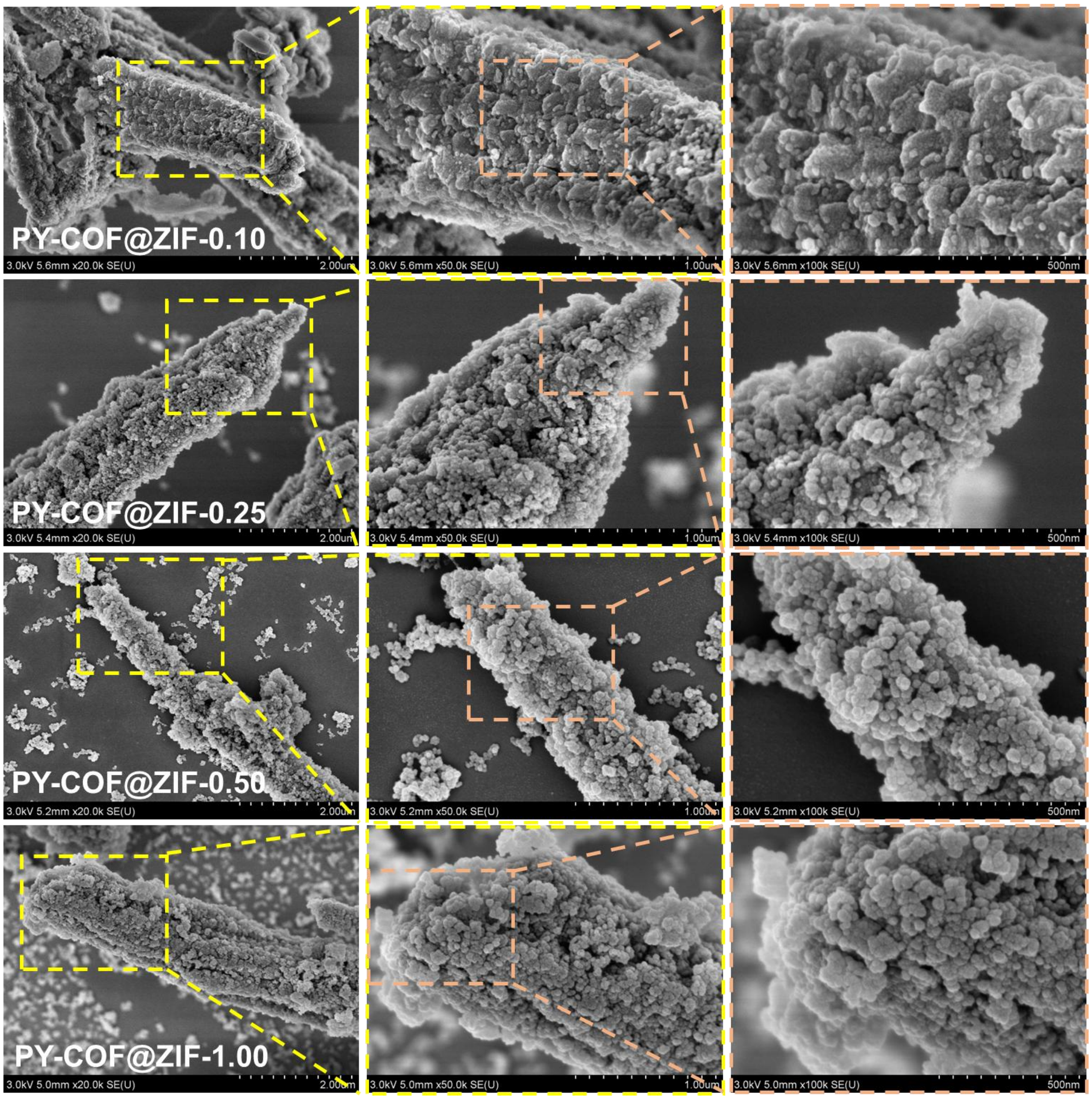

Fig. S10 SEM of PY-COF@ZIF-X, X=0.10~1.00.

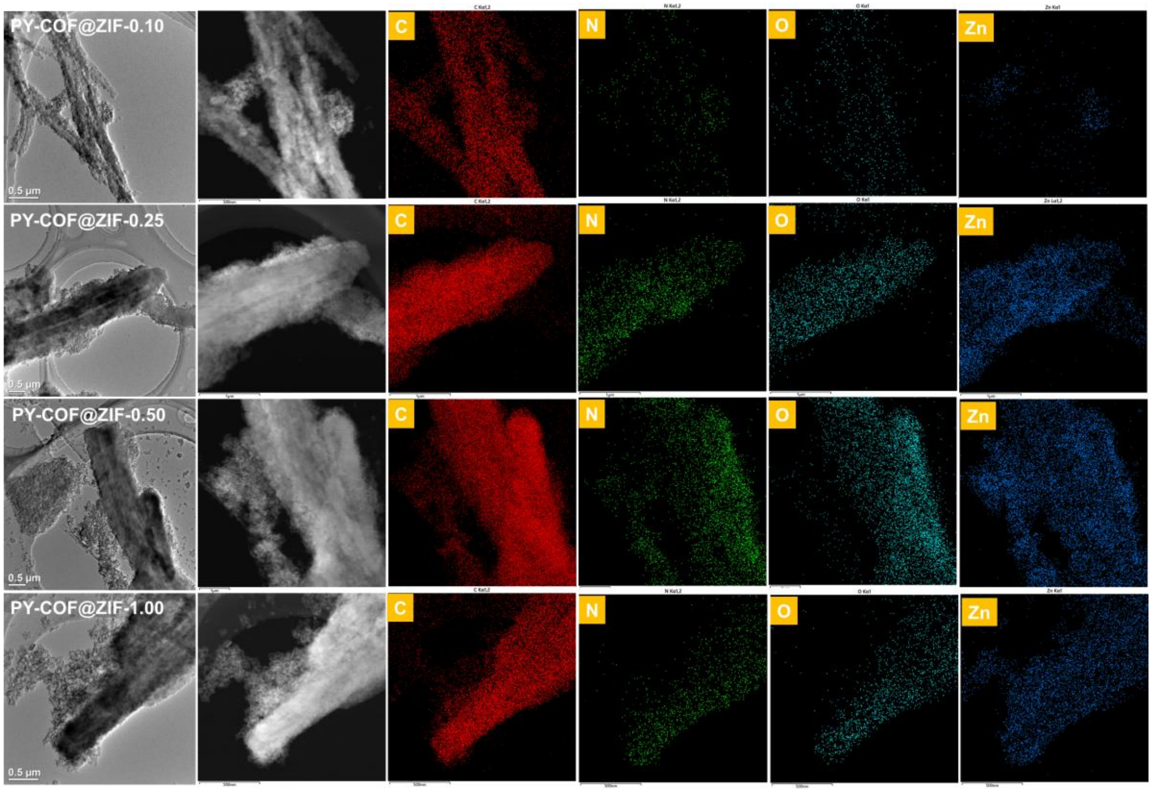

Fig. S11 TEM and Element mapping of PY-COF@ZIF-X, X=0.10~1.00.

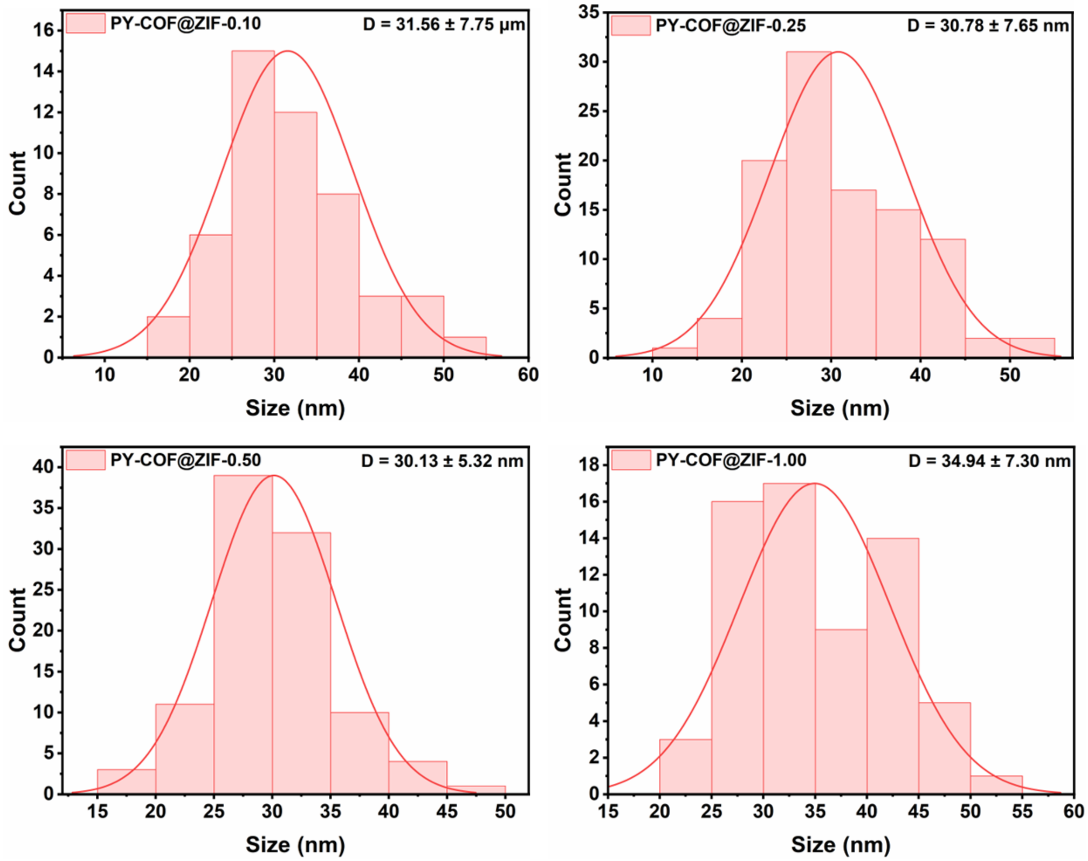

Fig. S12 The granularity statistics results of PY-COF@ZIF-X, X=0.10~1.00.

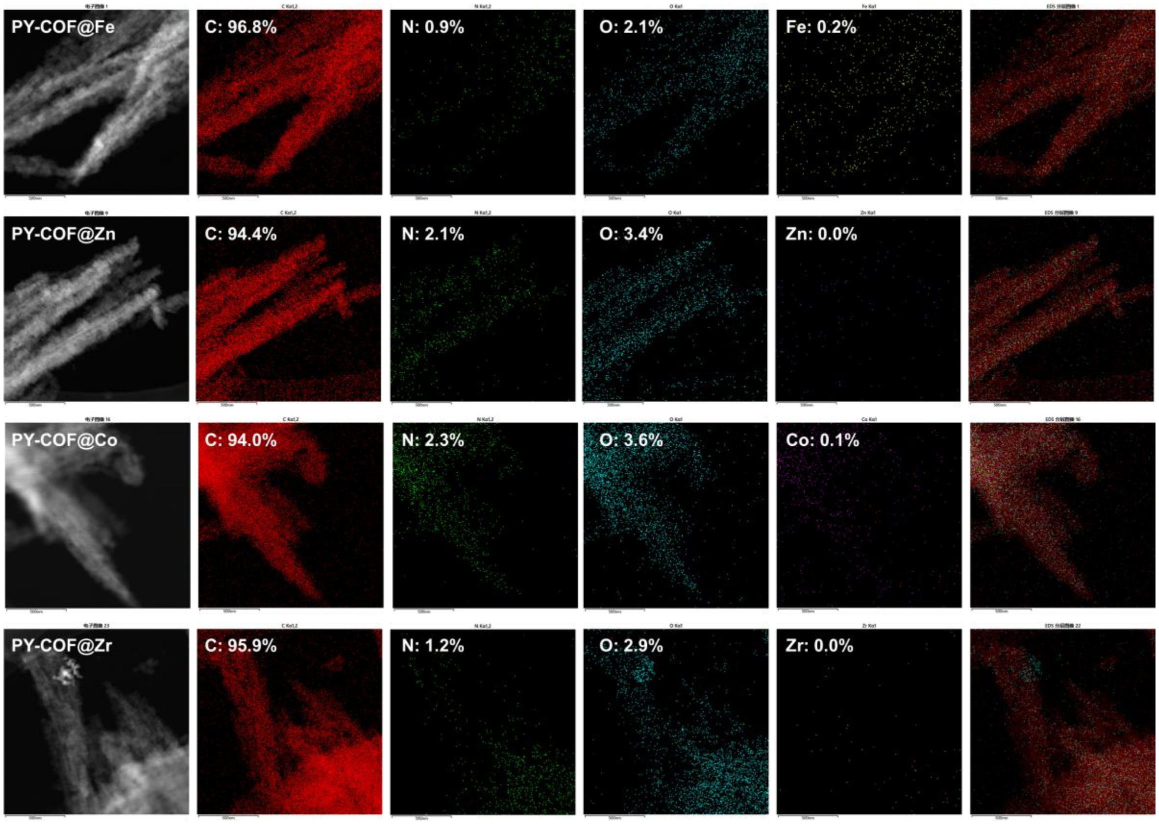

Fig. S13 Element mapping of PY-COF@M (M=Fe/Zn/Co/Zr).

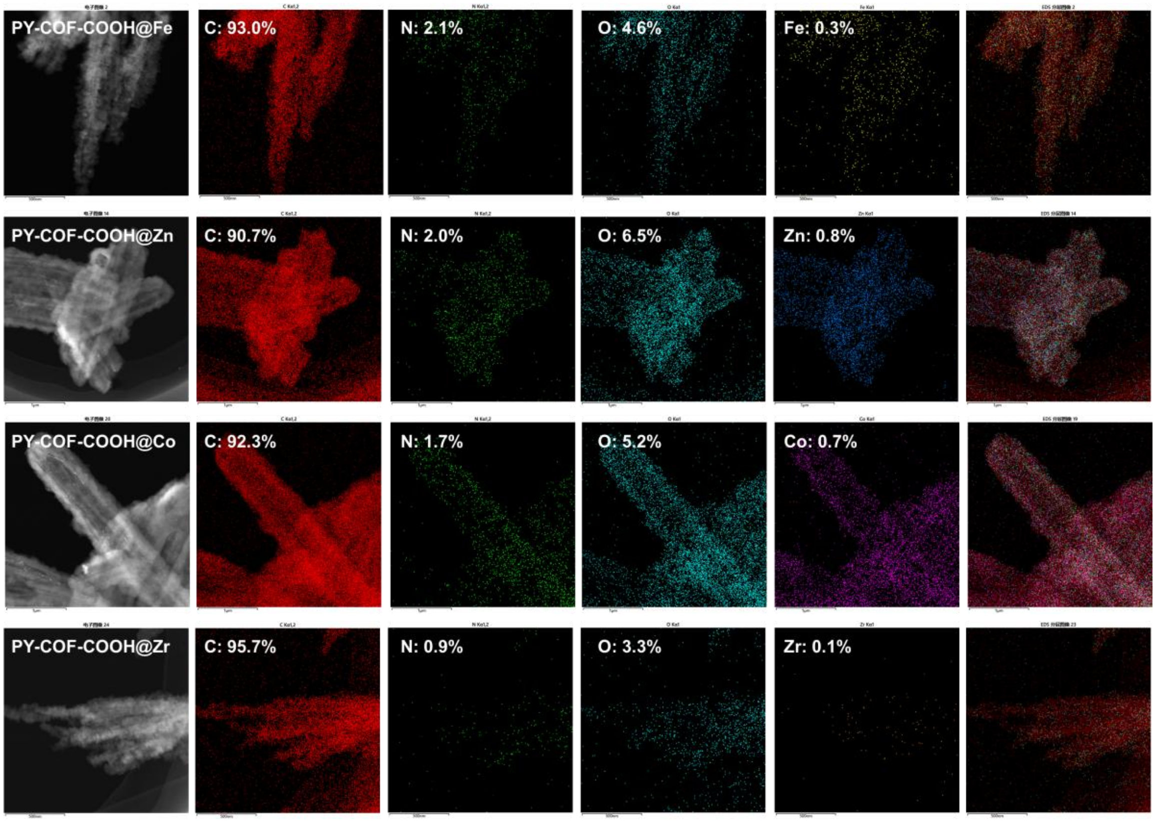

Fig. S14 Element mapping of PY-COF-COOH@M (M=Fe/Zn/Co/Zr).

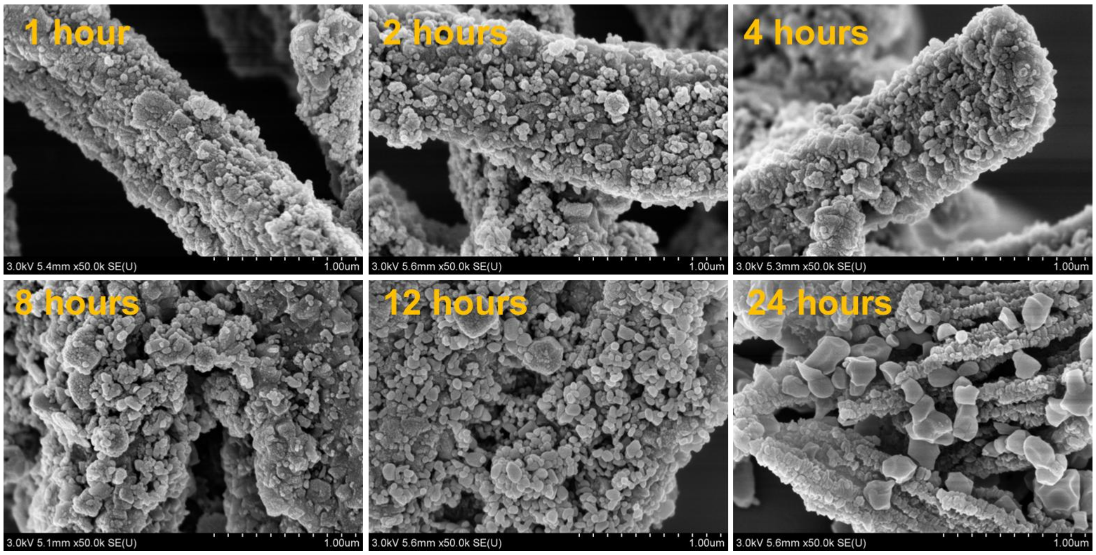

Fig. S15 SEM of PY-COF-COOH@M (M=Fe/Zn/Co/Zr).

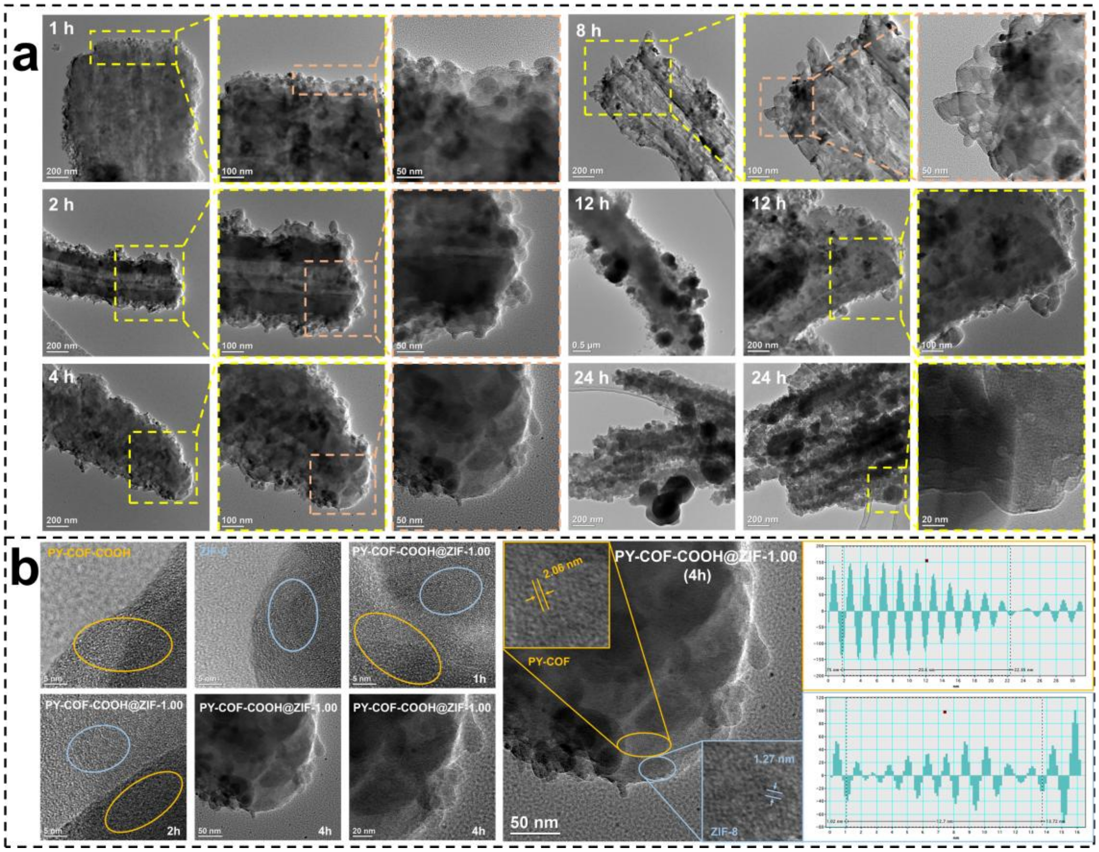

Fig. S16 a, HRTEM of the PY-COF-COOH@ZIF-1.00 at 1, 2, 4, 8, 12, and 24 h. **b**, Observation results of the lattice stripes of PY-COF-COOH@ZIF-1.0.

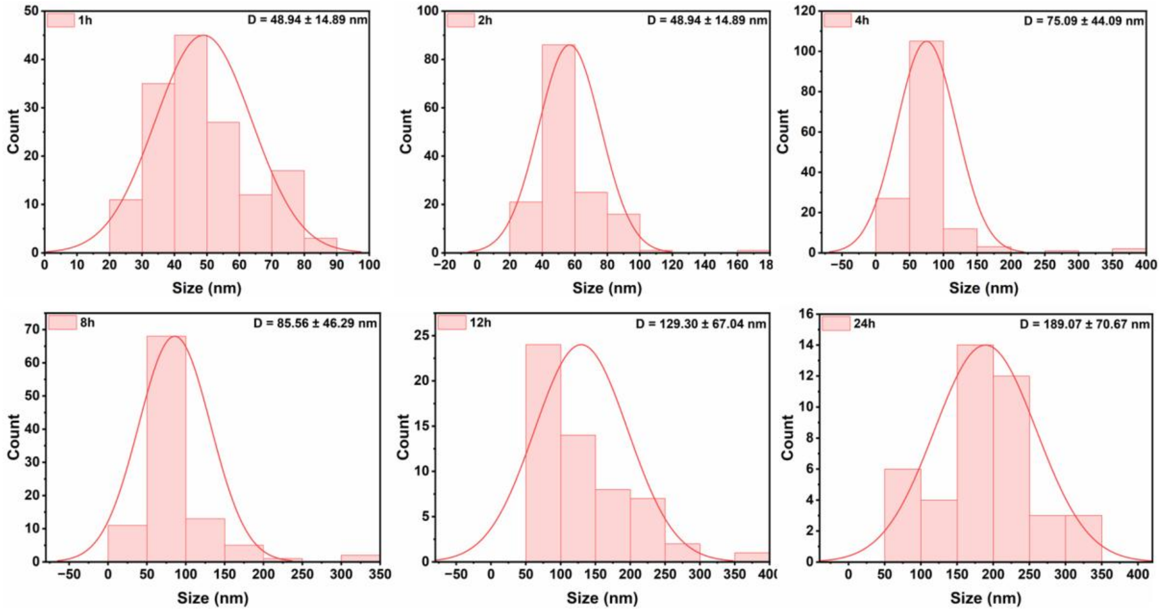

Fig. S17 The granularity statistics results of PY-COF-COOH@ZIF-1.00.

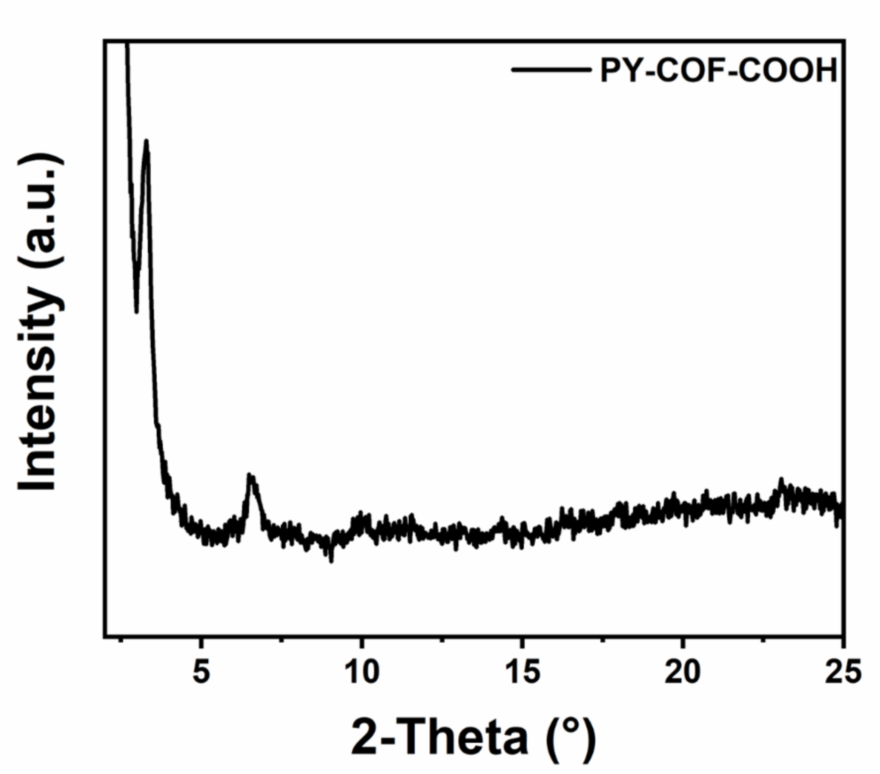

Fig. S18 PXRD spectrogram of PY-COF-COOH after reaction.

[Note] The obtained PY-COF-COOH was added to the system without Zn^{2+} and 2-MI for the synthesis of PY-COF@ZIF-X using S2.2.4. The obtained product was characterized by PXRD following the consistent processing steps: 2-Theta=1.5~30°, 2°/min.

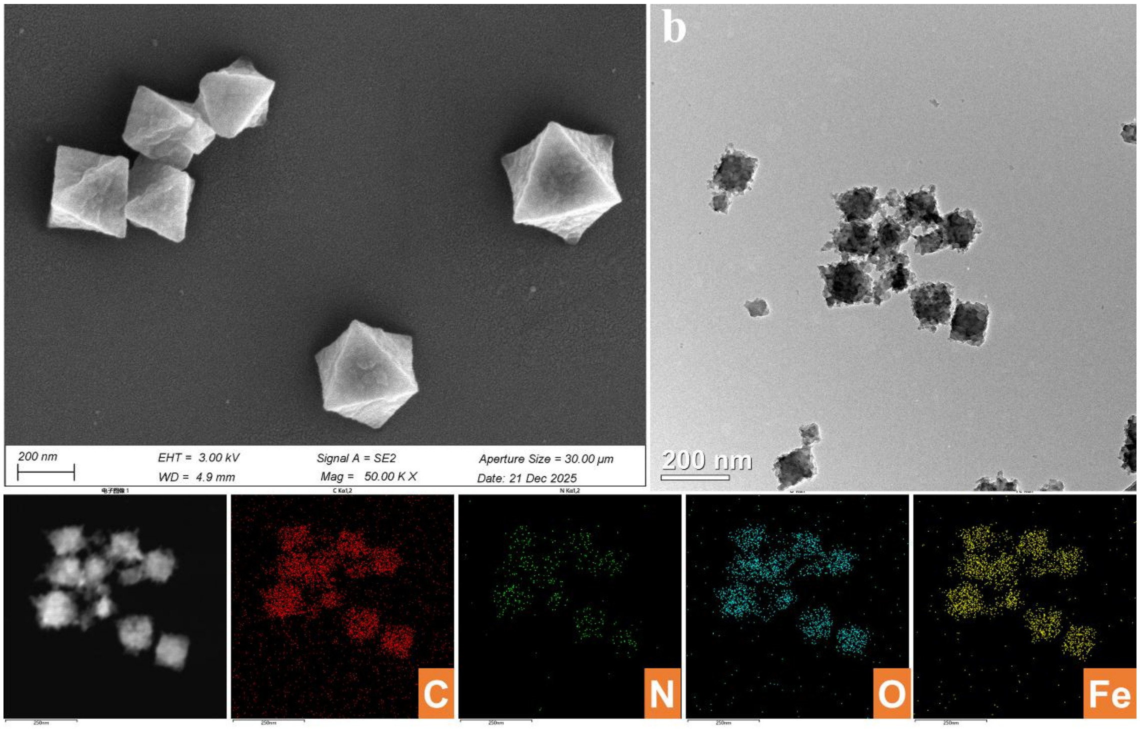

Fig. S19 SEM (a), HR-TEM (b) and element mapping of Fe-MOF.

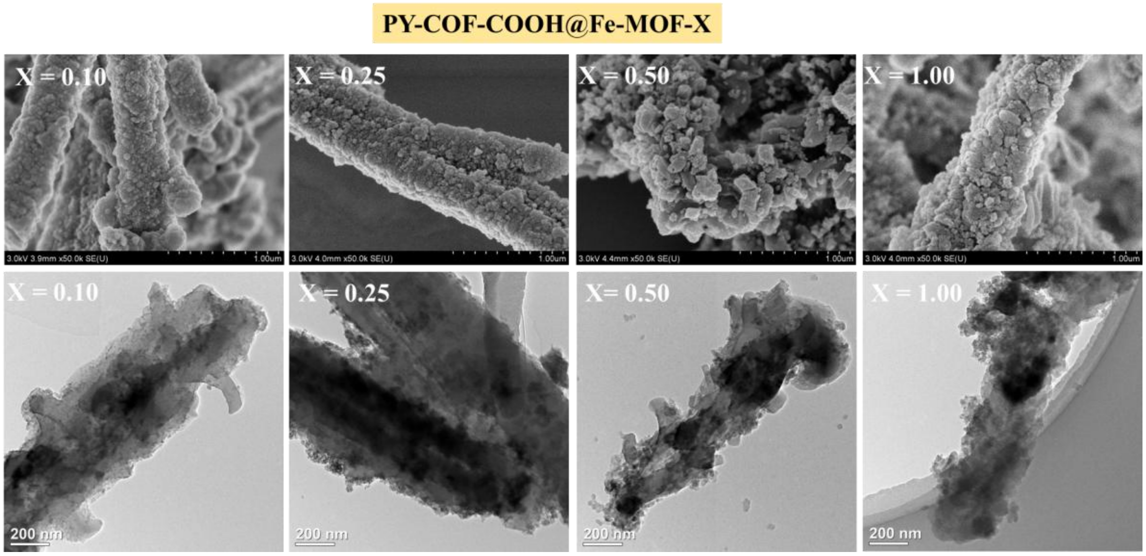

Fig. S20 SEM (Top) and HR-TEM (Bottom) of PY-COF-COOH@Fe-MOF-X (X=0.10~1.00).

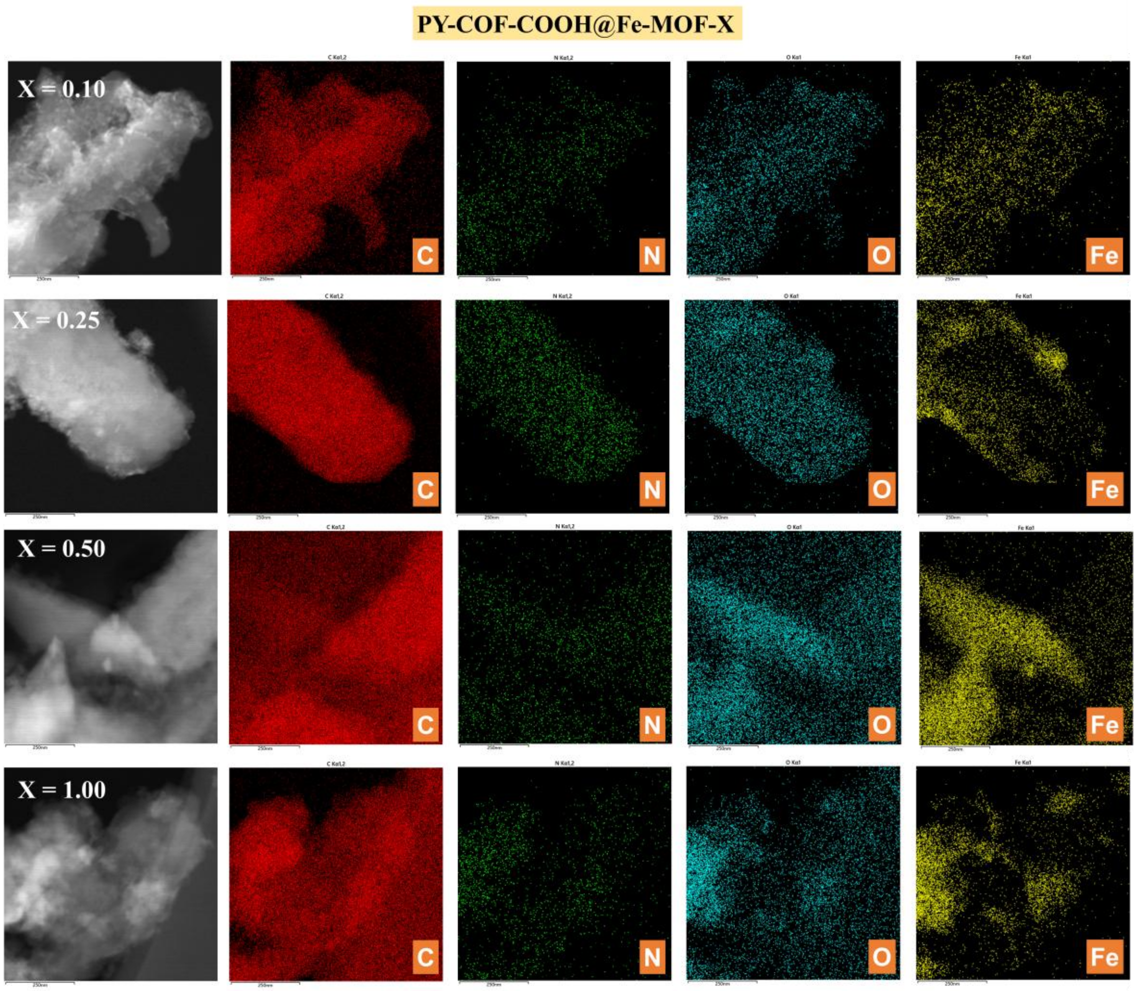

Fig. S21 Element mapping of PY-COF-COOH@Fe-MOF-X (X=0.10~1.00).

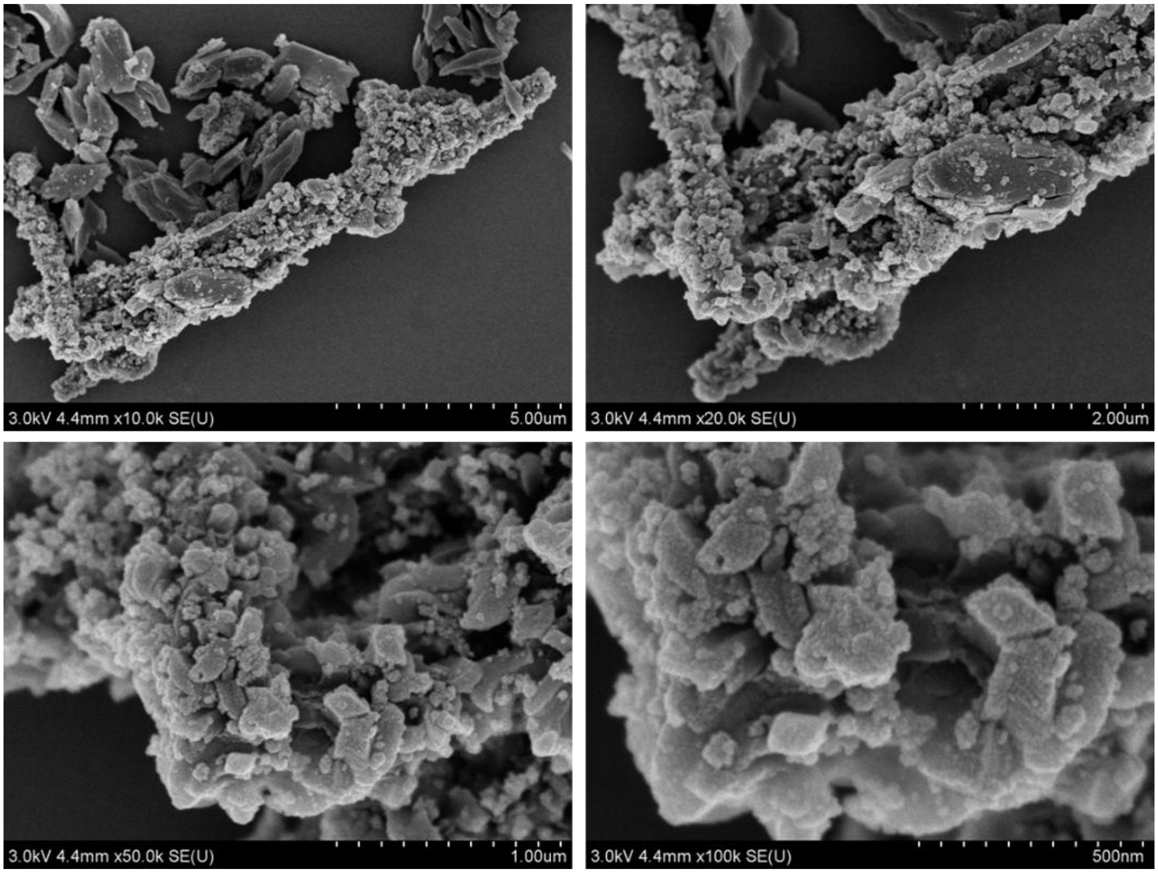

Fig. S22 SEM images at different magnifications of PY-COF-COOH@Fe-MOF-0.50.

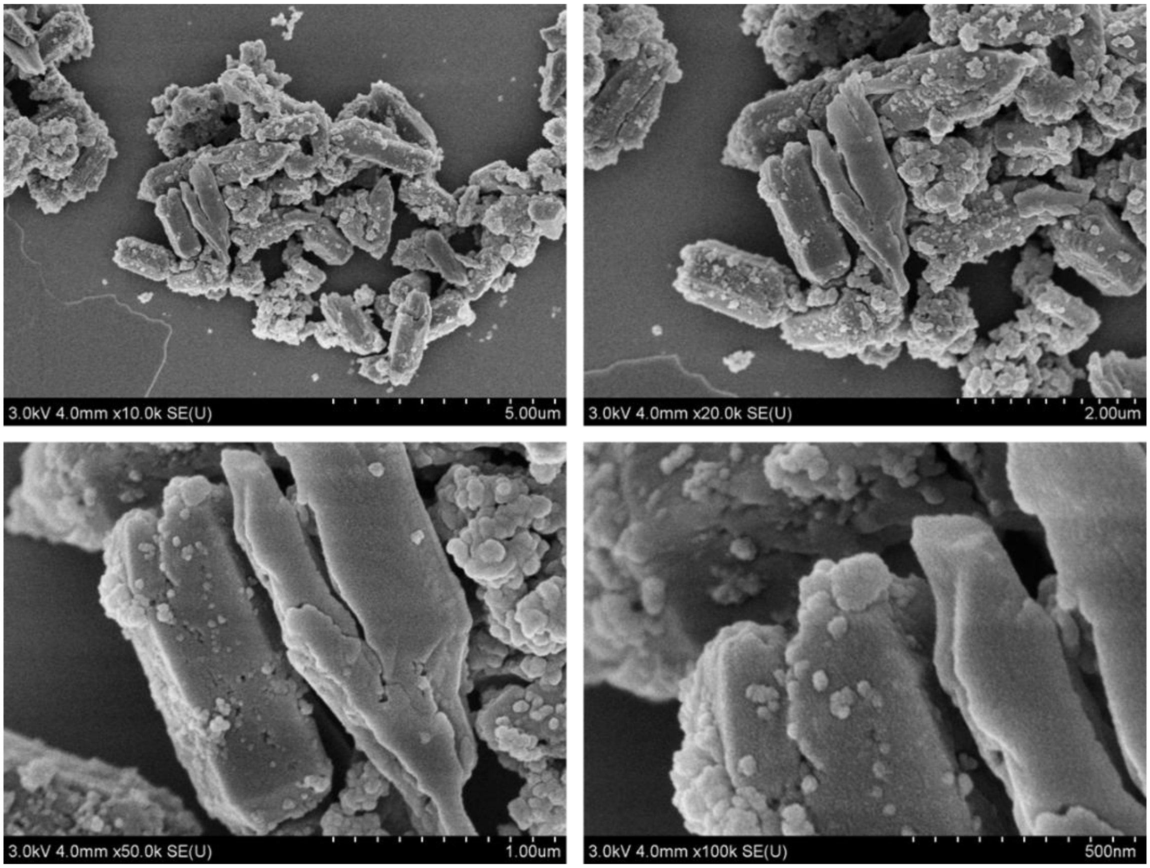

Fig. S23 SEM images at different magnifications of PY-COF-COOH@Fe-MOF-1.00.

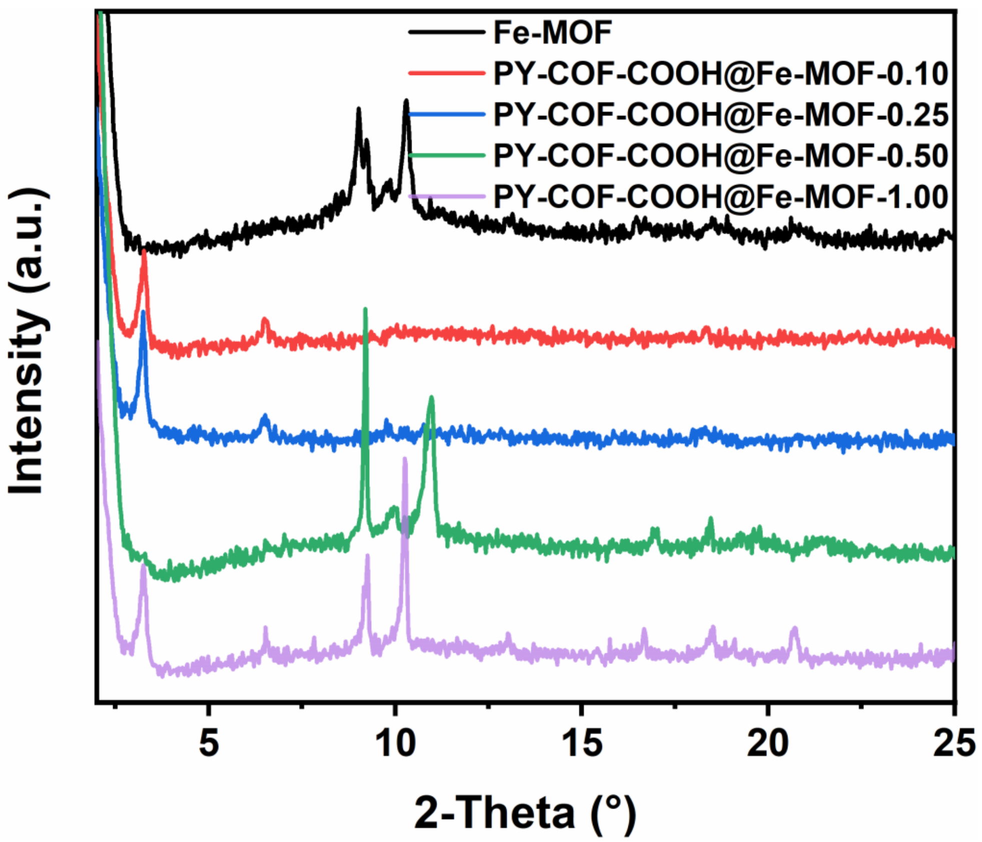

Fig. S24 PXRD of PY-COF-COOH@Fe-MOF-X (X=0.10~1.00).

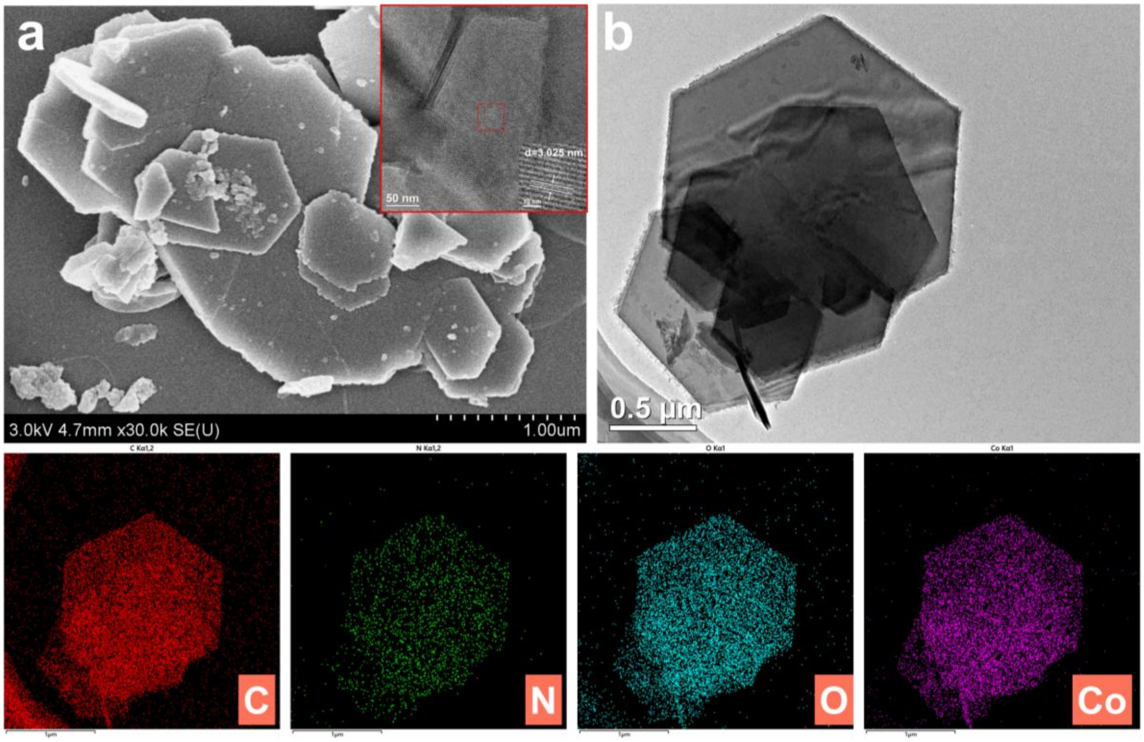

Fig. S25 SEM (a), HR-TEM (b) and element mapping of Co-MOF.

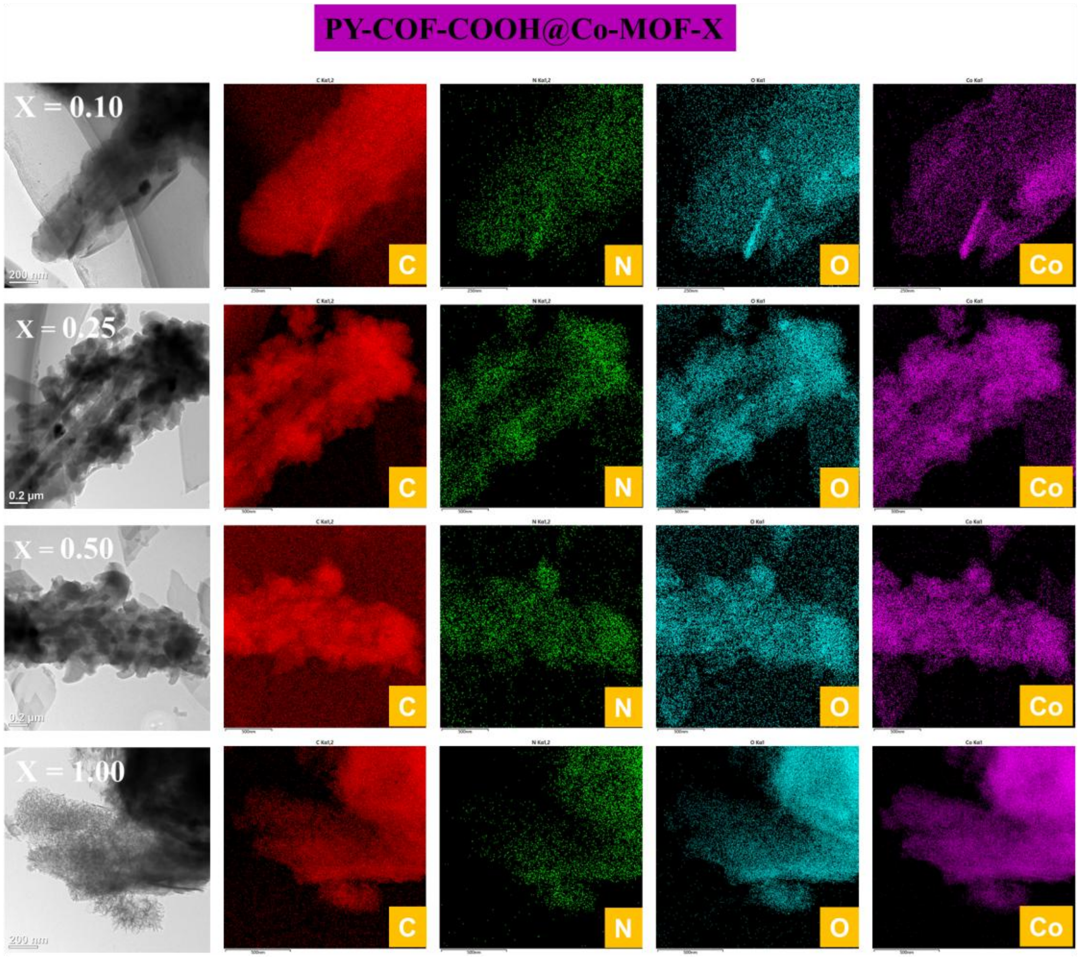

Fig. S26 HR-TEM and Element mapping of PY-COF-COOH@Co-MOF-X(X=0.10~1.00).

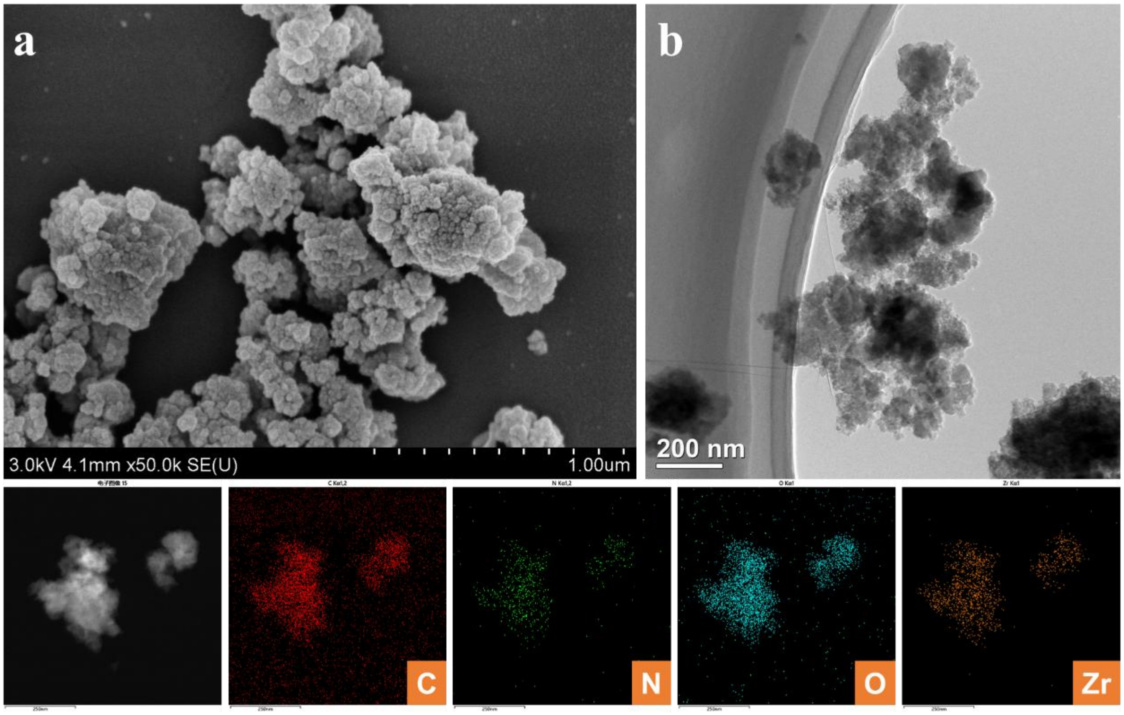

Fig. S27 SEM (a), HR-TEM (b) and element mapping of UIO-66.

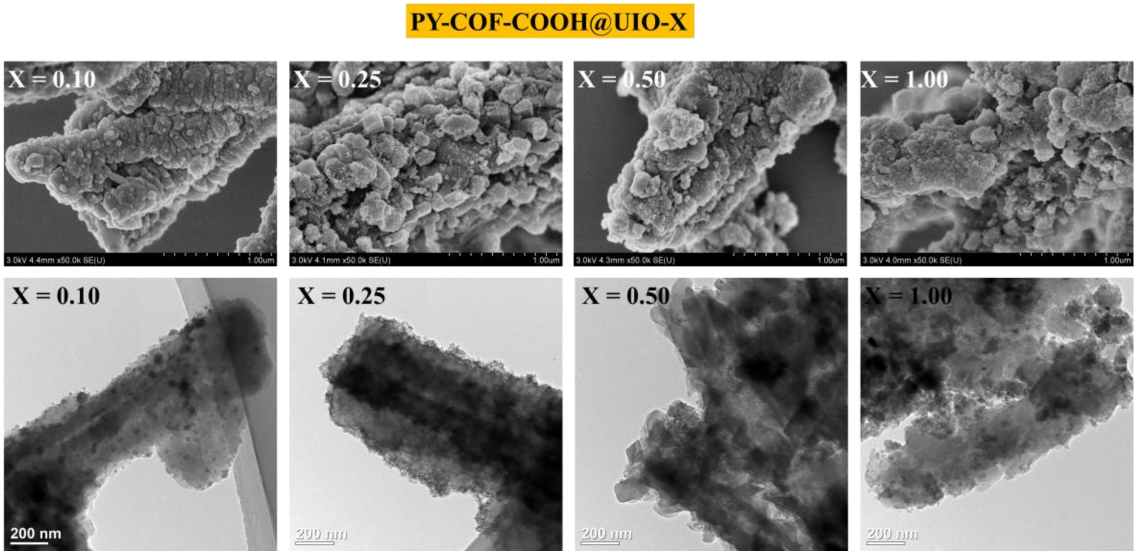

Fig. S28 SEM (Top) and HR-TEM (Bottom) of PY-COF-COOH@UIO-X (X=0.10~1.00).

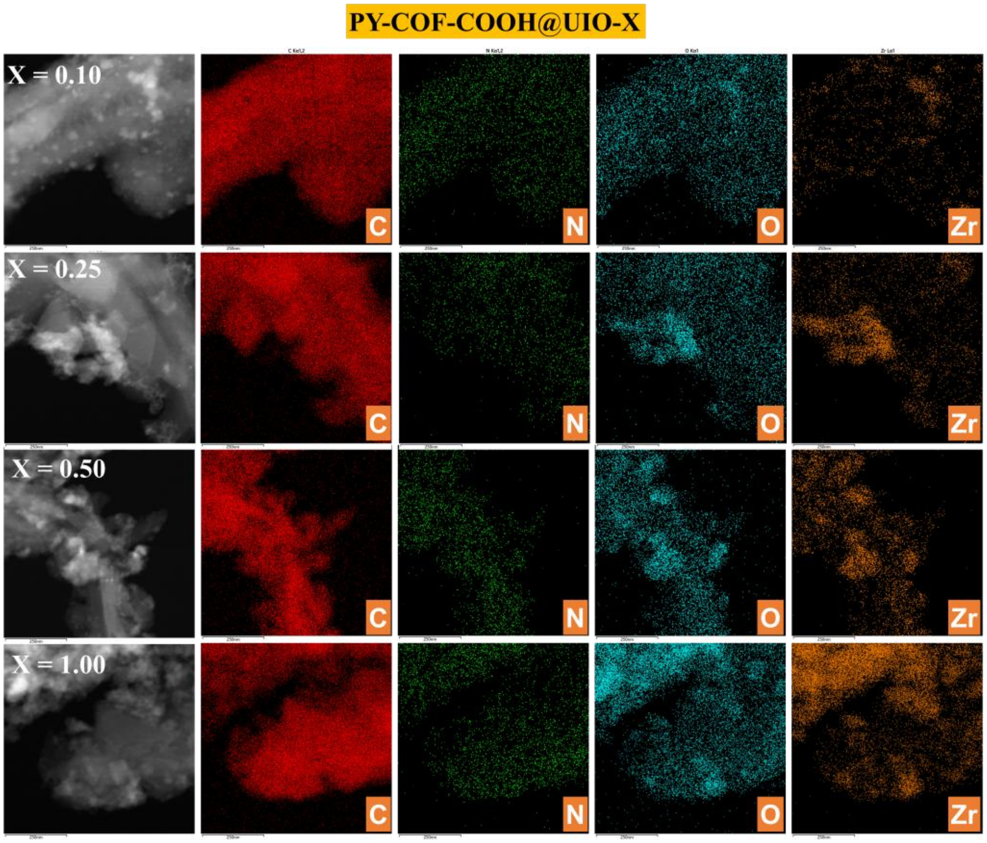

Fig. S29 Element mapping of PY-COF-COOH@UIO-X (X=0.10, 0.25. 0.50 and 1.00).

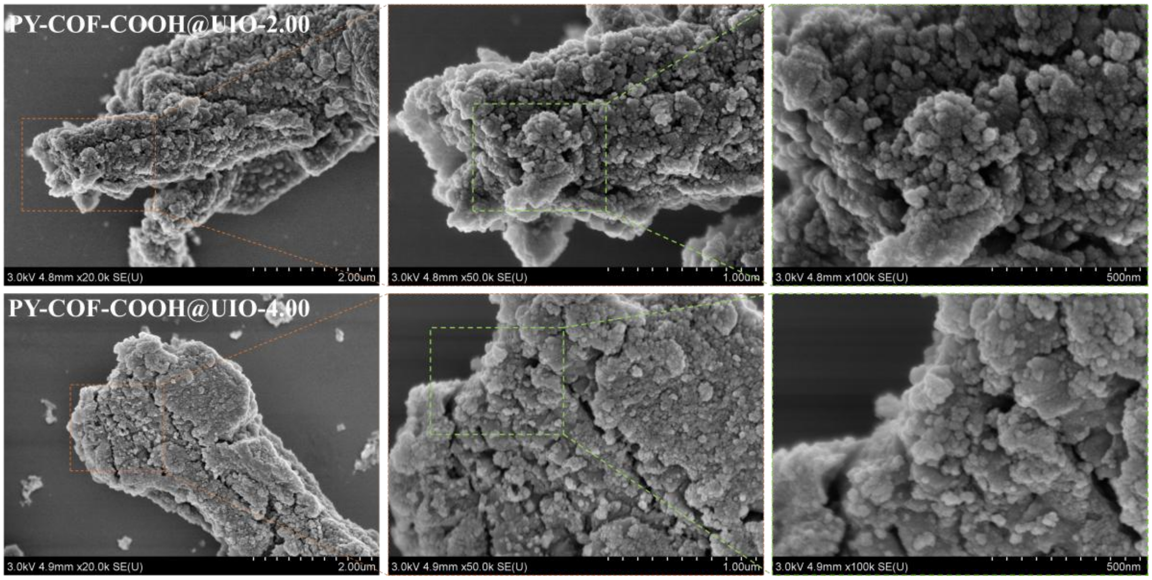

Fig. S30 SEM of PY-COF-COOH@UIO-X (X=2.00 and 4.00).

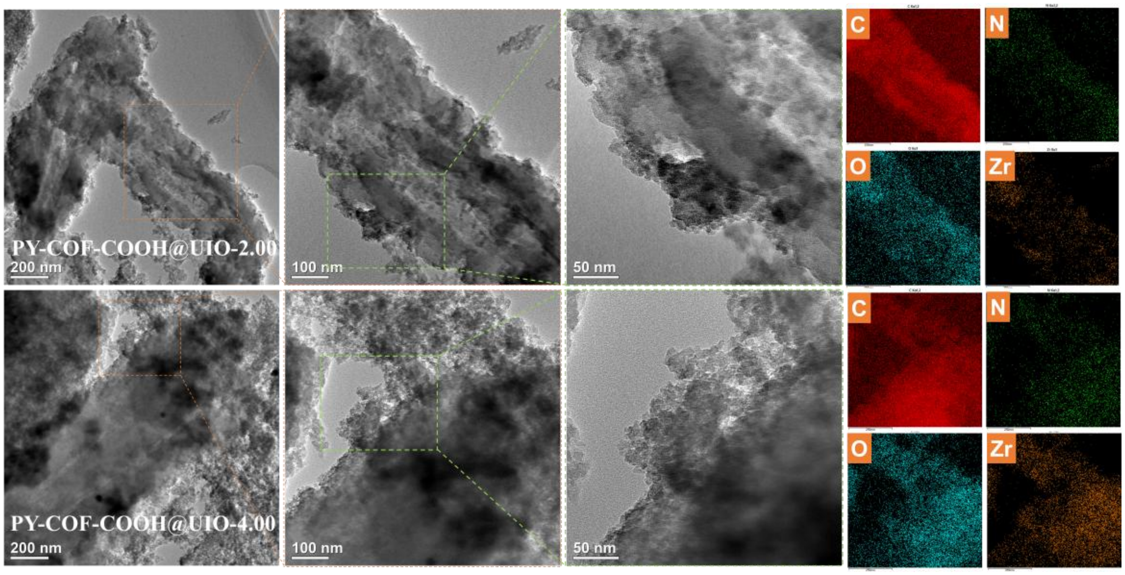

Fig. S31 HR-TEM and element mapping of PY-COF-COOH@UIO-2.00 (Top) and PY-COF-COOH@UIO-4.00 (Botton).

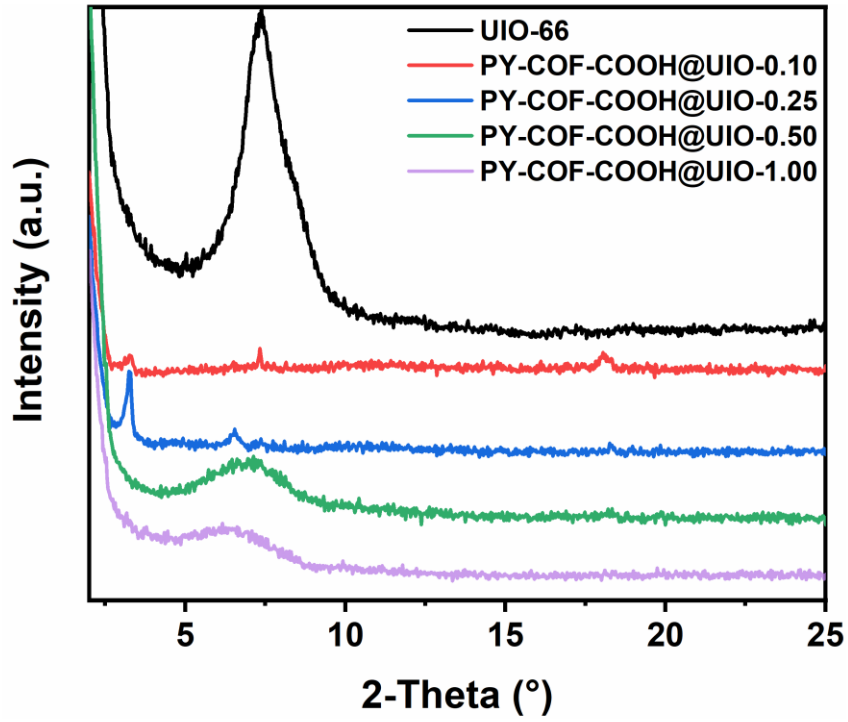

Fig. S32 PXRD of UIO-66 and PY-COF-COOH@UIO-X (X=0.10~1.00).

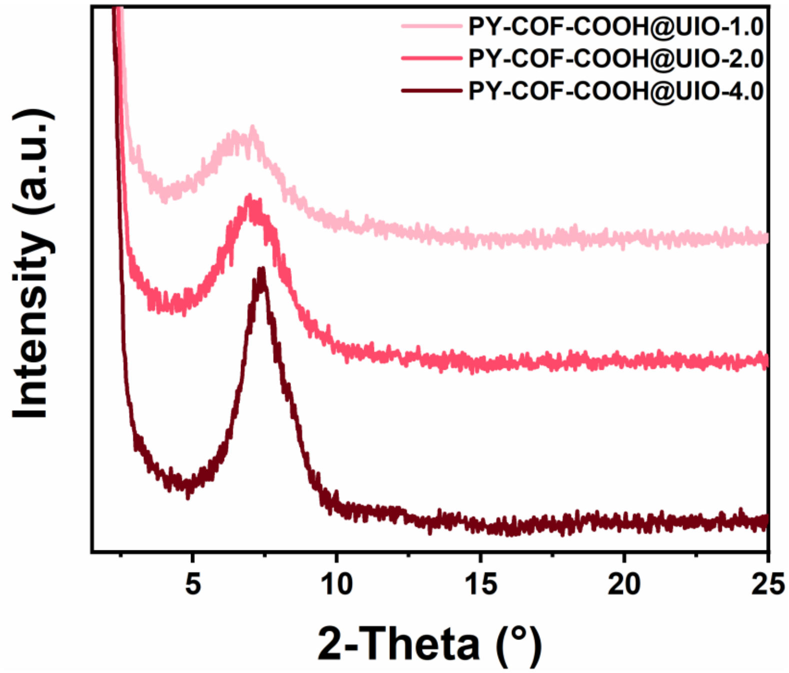

Fig. S33 PXRD of PY-COF-COOH@UIO-X (X=1.00, 2.00 and 4.00).

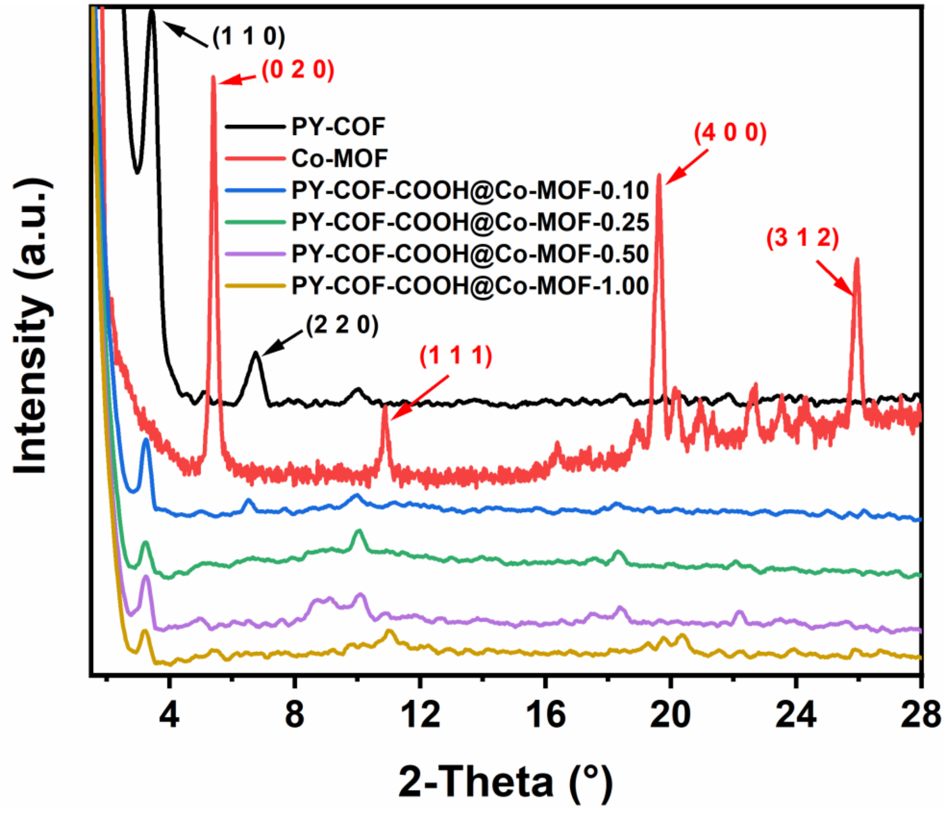

Fig. S34 PXRD of PY-COF-COOH@Co-MOF-X (X=0.10~1.00).

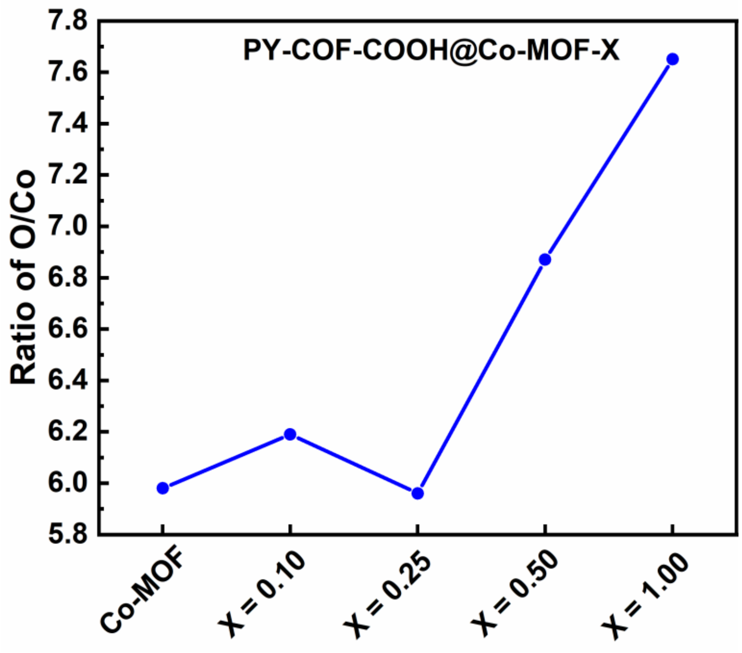

Fig. S35 The ratio of O/Co in PY-COF-COOH@Co-MOF (X=0.10~1.00).

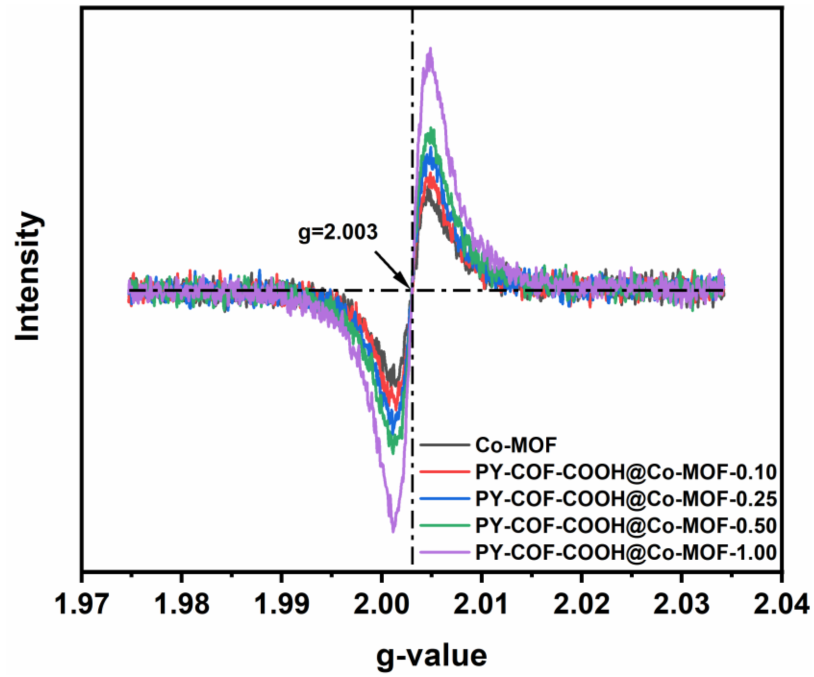

Fig. S36 Oxygen vacancy of PY-COF-COOH@Co-MOF (X=0.10~1.00).

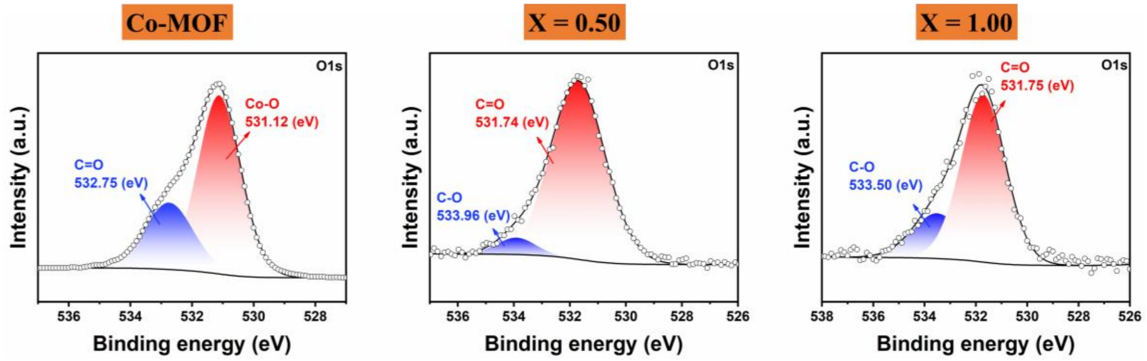

Fig. S37 O1s of Co-MOF and PY-COF-COOH@Co-MOF (X=0.50 and 1.00)

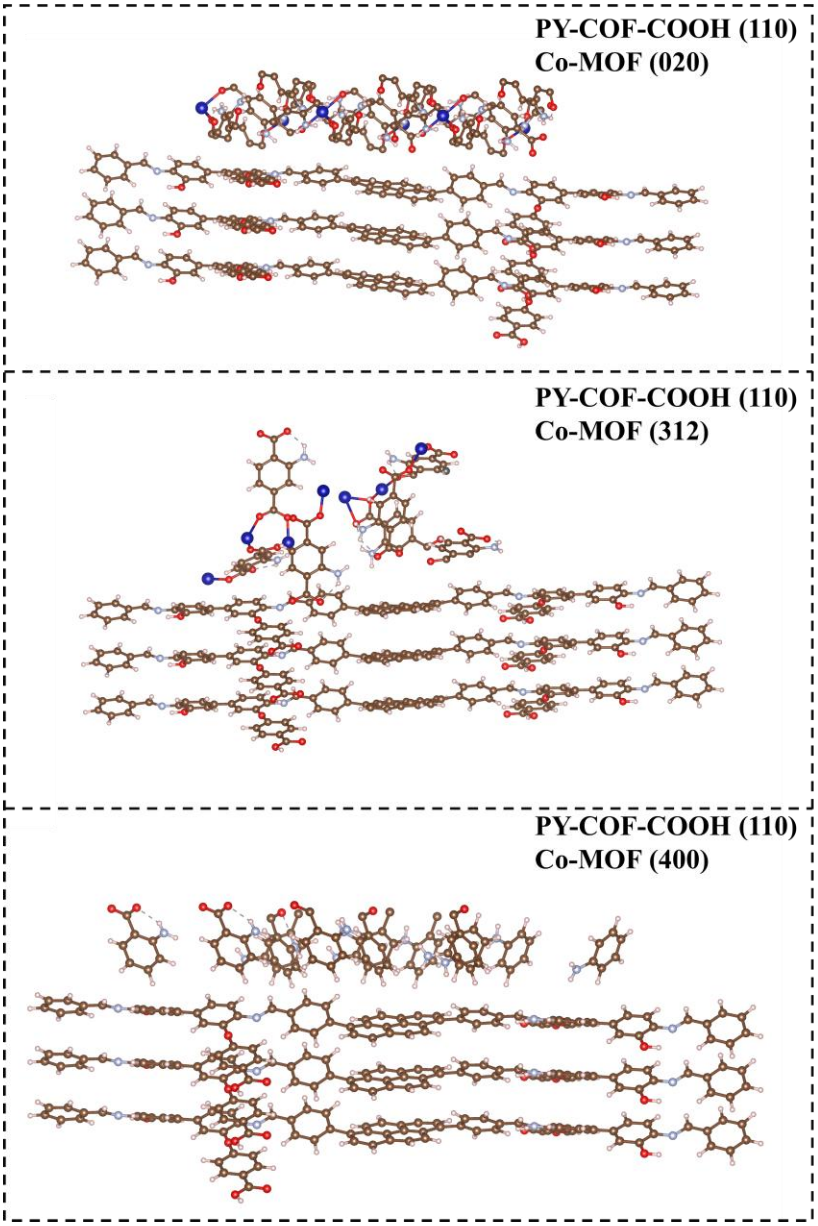

Fig. S38 Schematic diagram of crystal plane bonding between PY-COF-COOH (110) and Co-COF (020), (312) and (400), respectively.

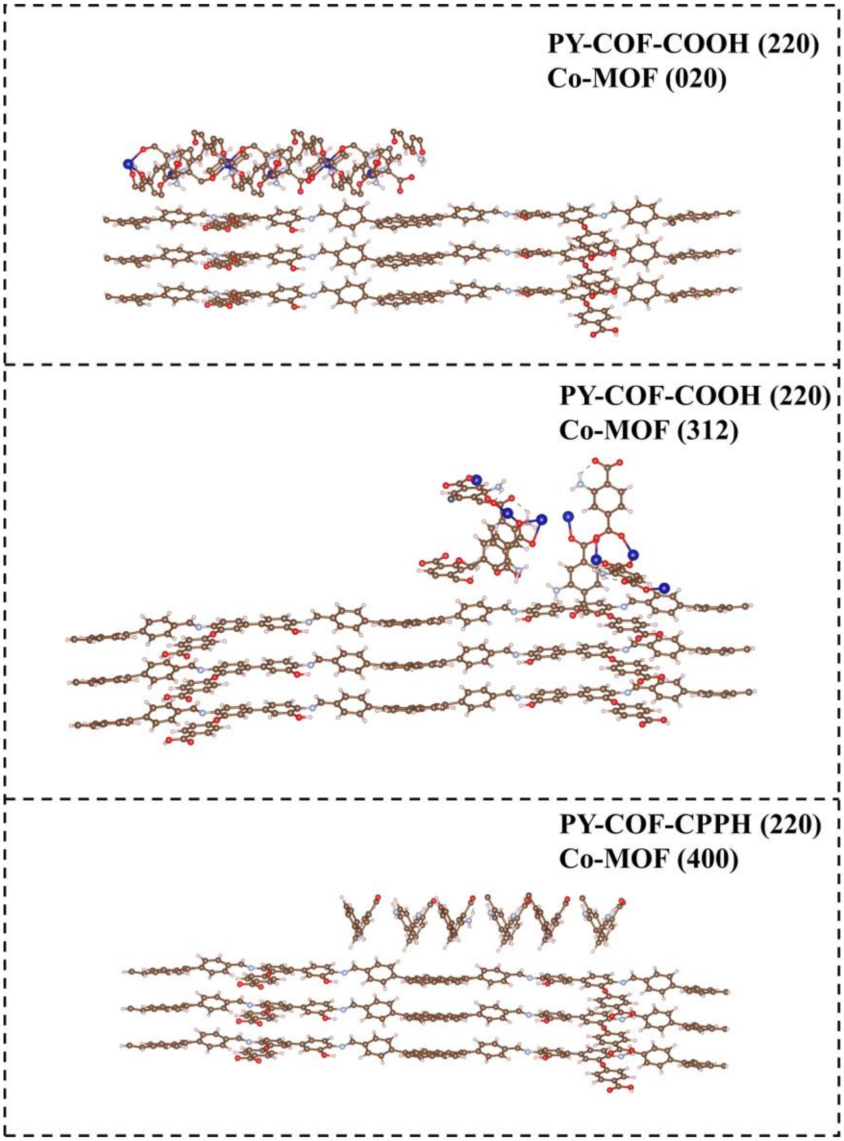

Fig. S39 Schematic diagram of crystal plane bonding between PY-COF-COOH (220) and Co-COF (020), (312) and (400), respectively.

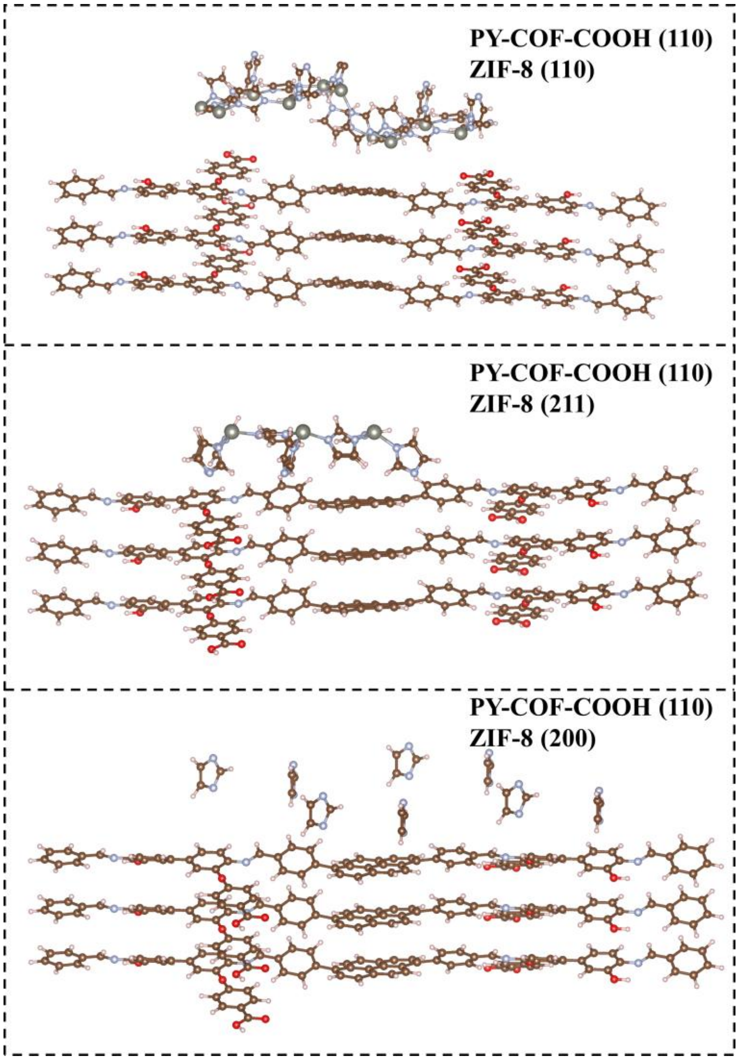

Fig. S40 Schematic diagram of crystal plane bonding between PY-COF-COOH (110) and ZIF-8 (110), (211) and (200), respectively.

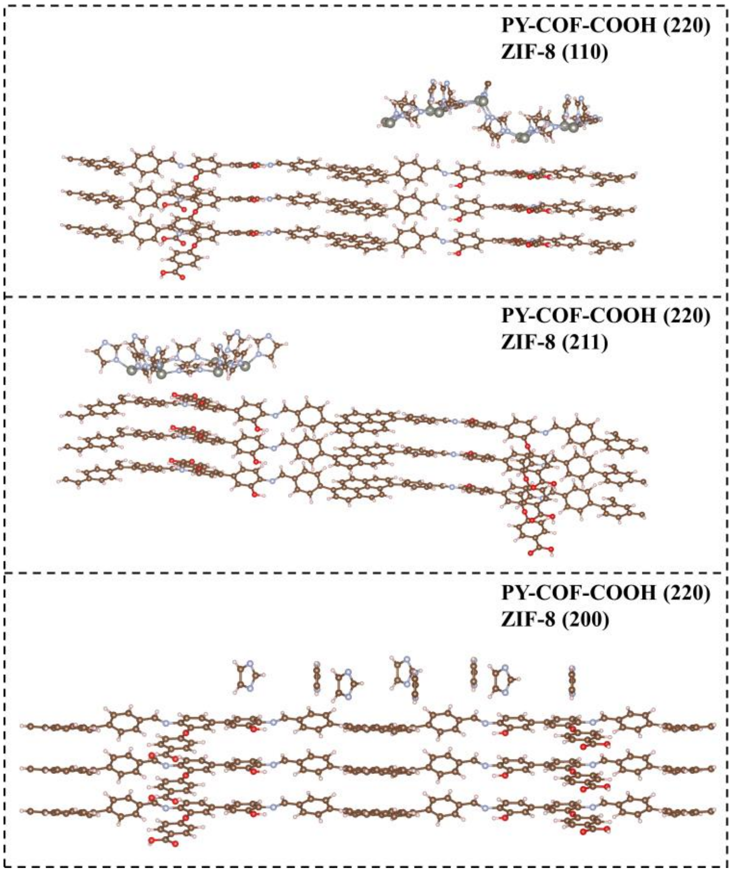

Fig. S41 Schematic diagram of crystal plane bonding between PY-COF-COOH (220) and ZIF-8 (110), (211) and (200), respectively.

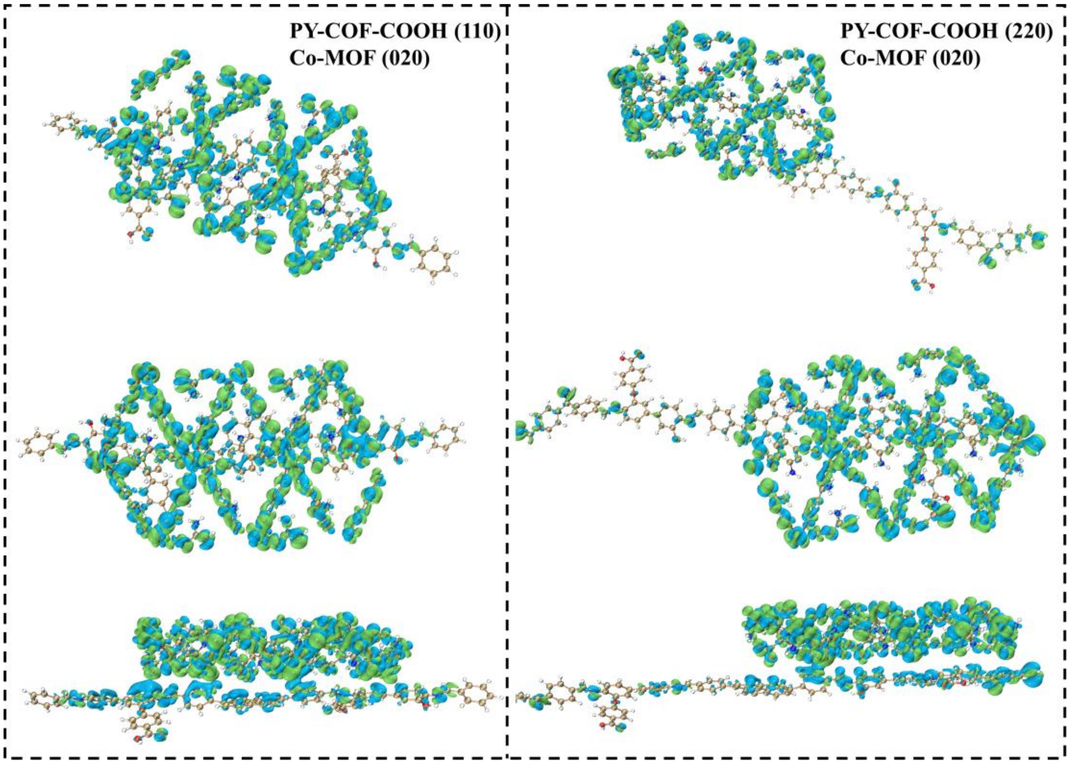

Fig. S42 The charge difference density map between Co-MOF (020) and PY-COF-COOH (110) and (220), respectively.

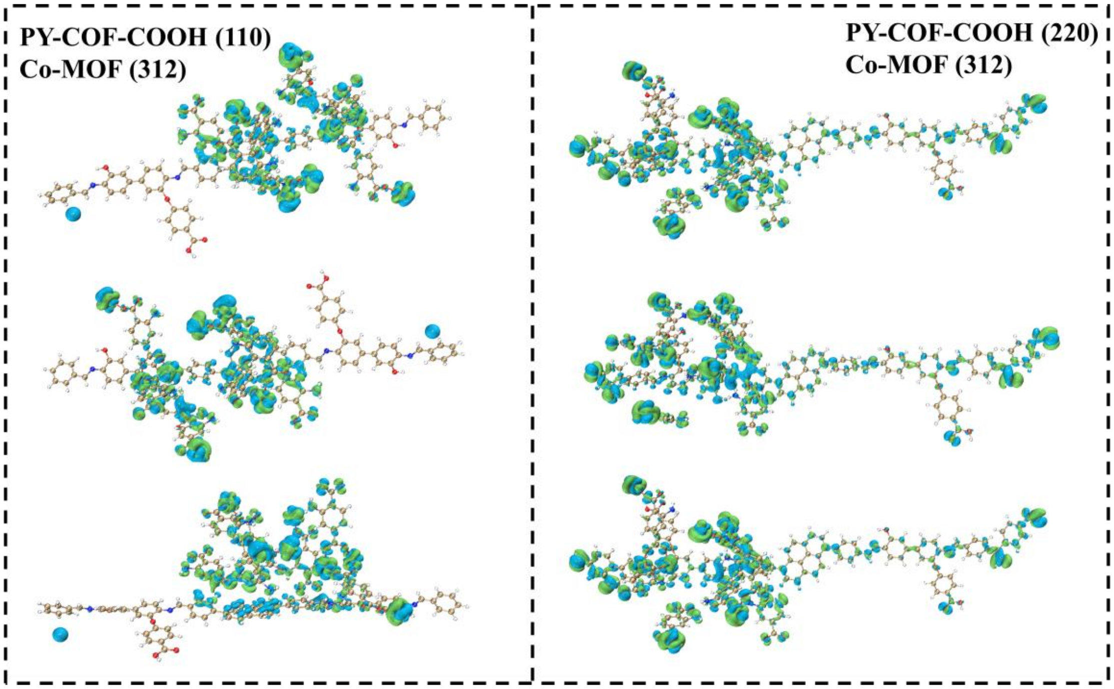

Fig. S43 The charge difference density map between Co-COF (312) and PY-COF-COOH (110) and (220), respectively.

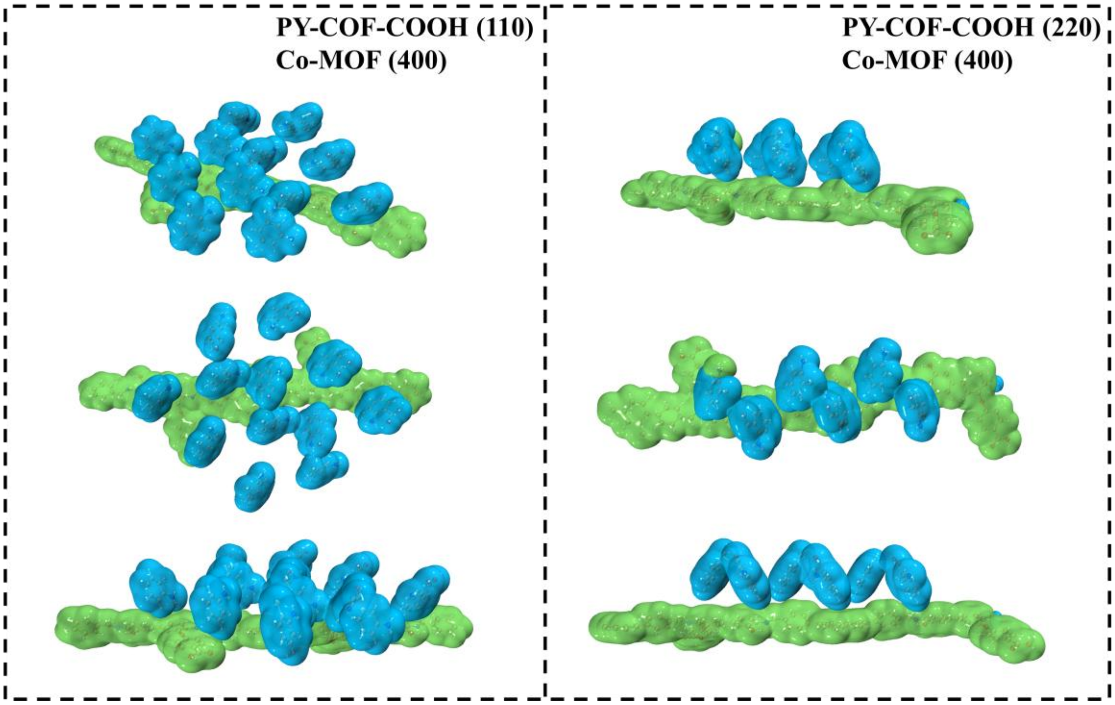

Fig. S44 The charge difference density map between Co-COF (400) and PY-COF-COOH (110) and (220), respectively.

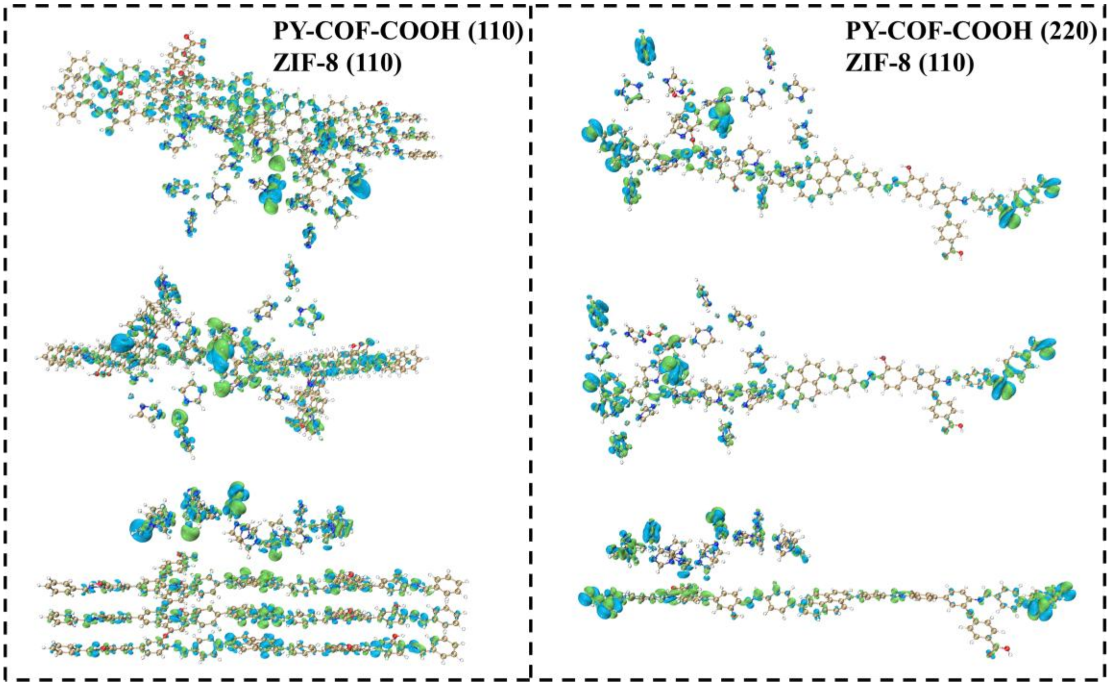

Fig. S45 The charge difference density map between ZIF-8 (110) and PY-COF-COOH (110) and (220), respectively.

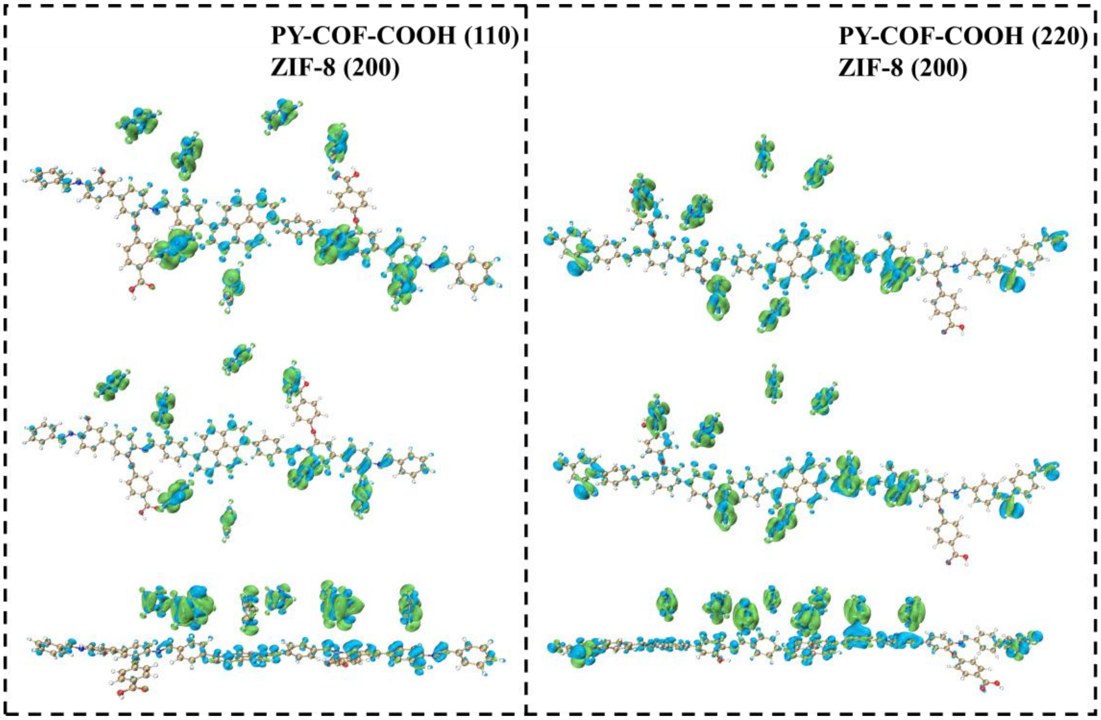

Fig. S46 The charge difference density map between ZIF-8 (200) and PY-COF-COOH (110) and (220), respectively.

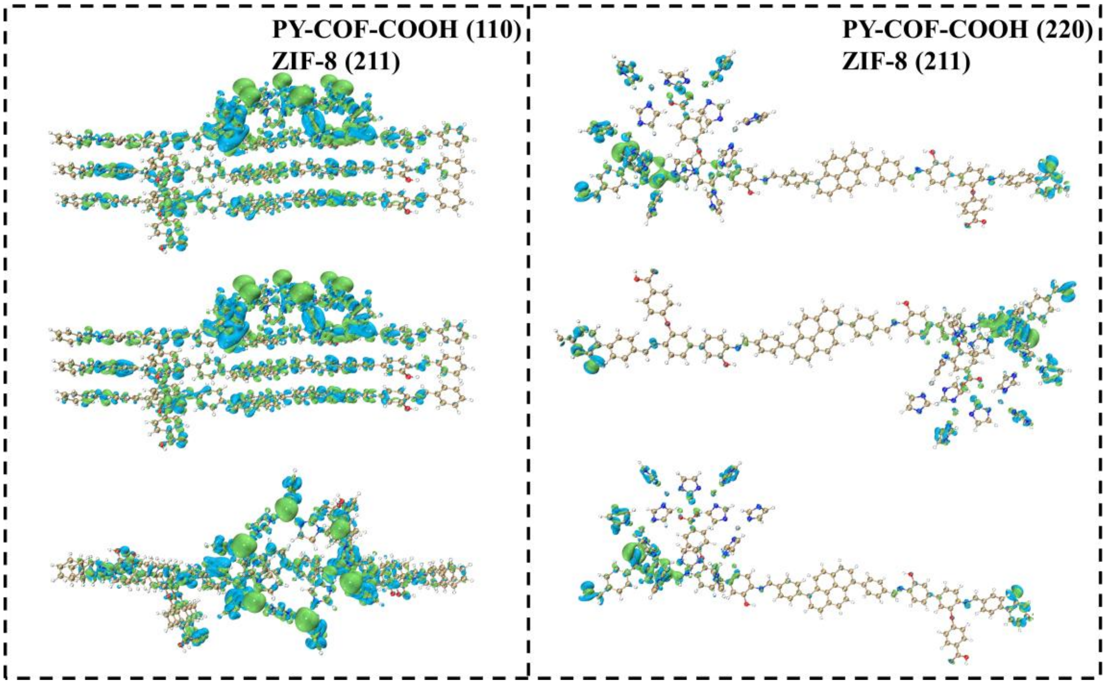

Fig. S47 The charge difference density map between ZIF-8 (200) and PY-COF-COOH (110) and (211), respectively.

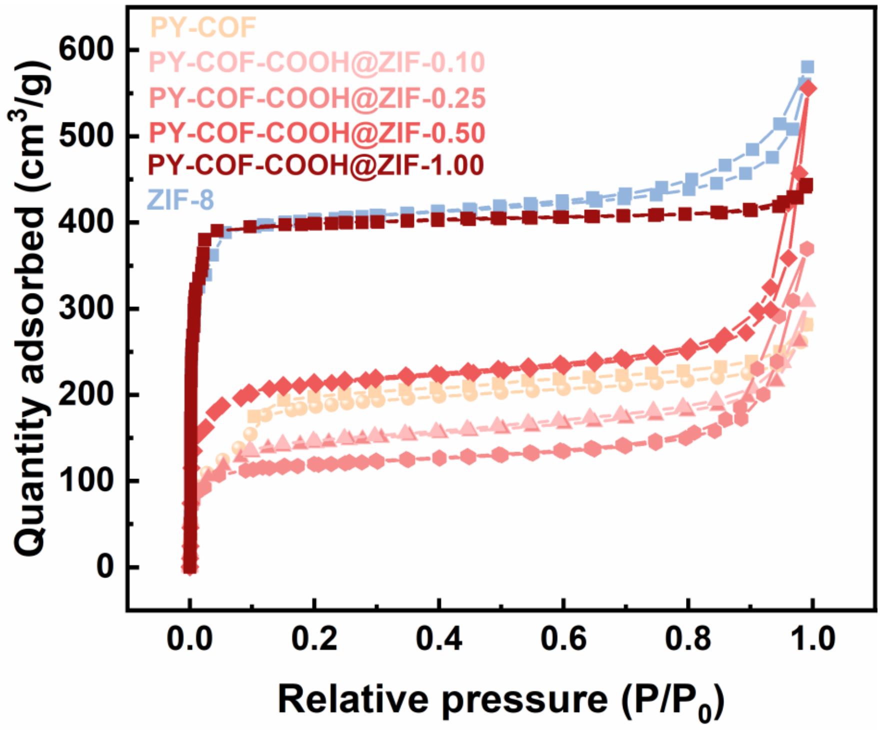

Fig. S48 The N_2 adsorption-desorption isotherm of PY-COF-COOH@ZIF-X (X=0.10~1.00).

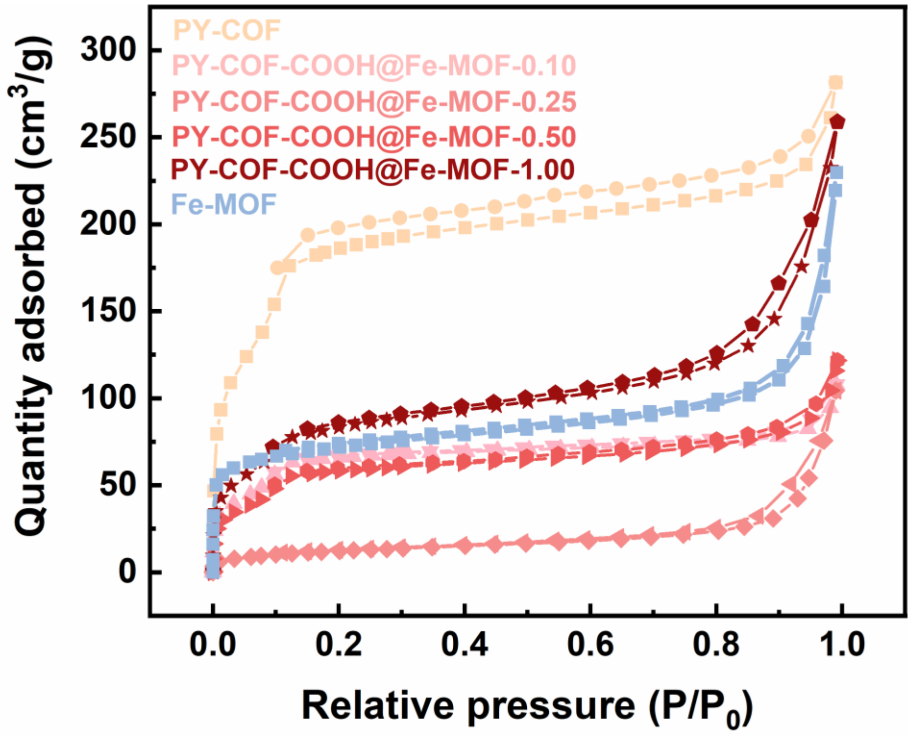

Fig. S49 The N_2 adsorption-desorption isotherm of PY-COF-COOH@Fe-MOF-X (X=0.10, 0.25, 0.50 and 1.00)

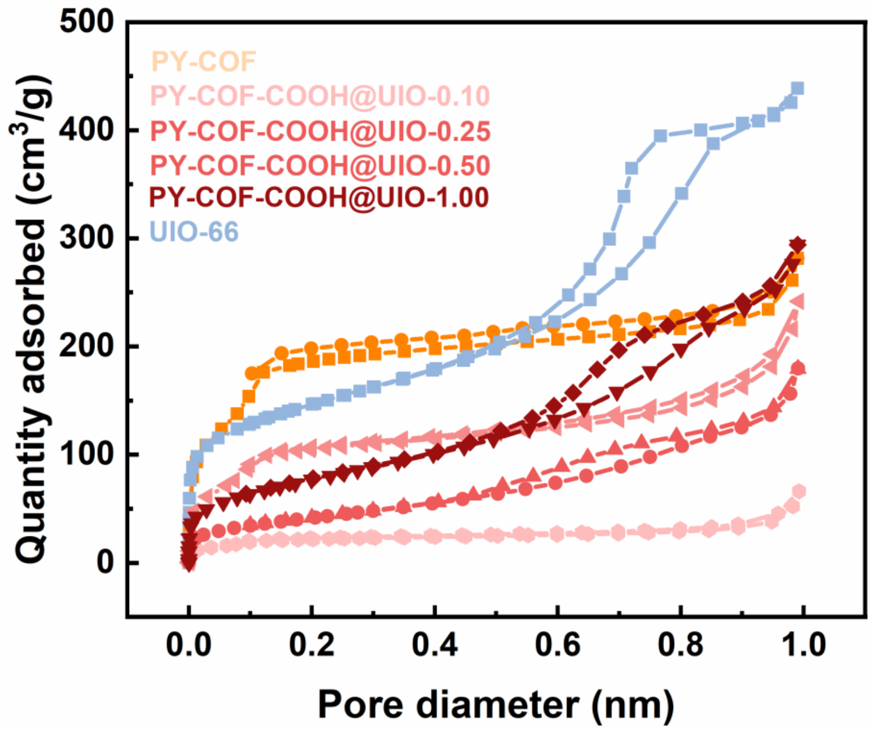

Fig. S50 The N_2 adsorption-desorption isotherm of PY-COF-COOH@UIO-X (X=0.10~1.00).

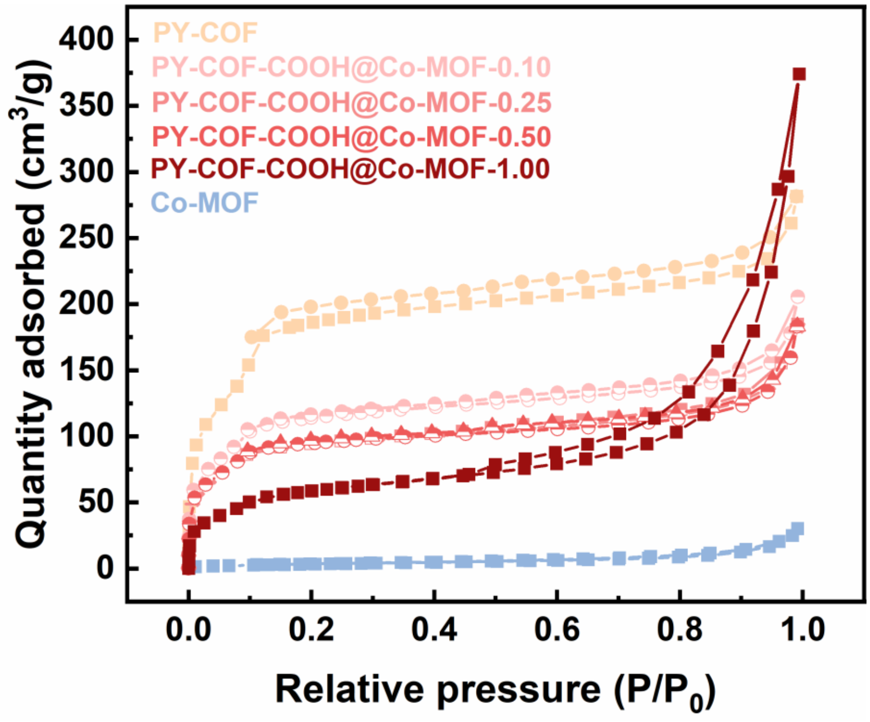

Fig. S51 The N_2 adsorption-desorption isotherm of PY-COF-COOH@Co-MOF-X (X=0.10~1.00)

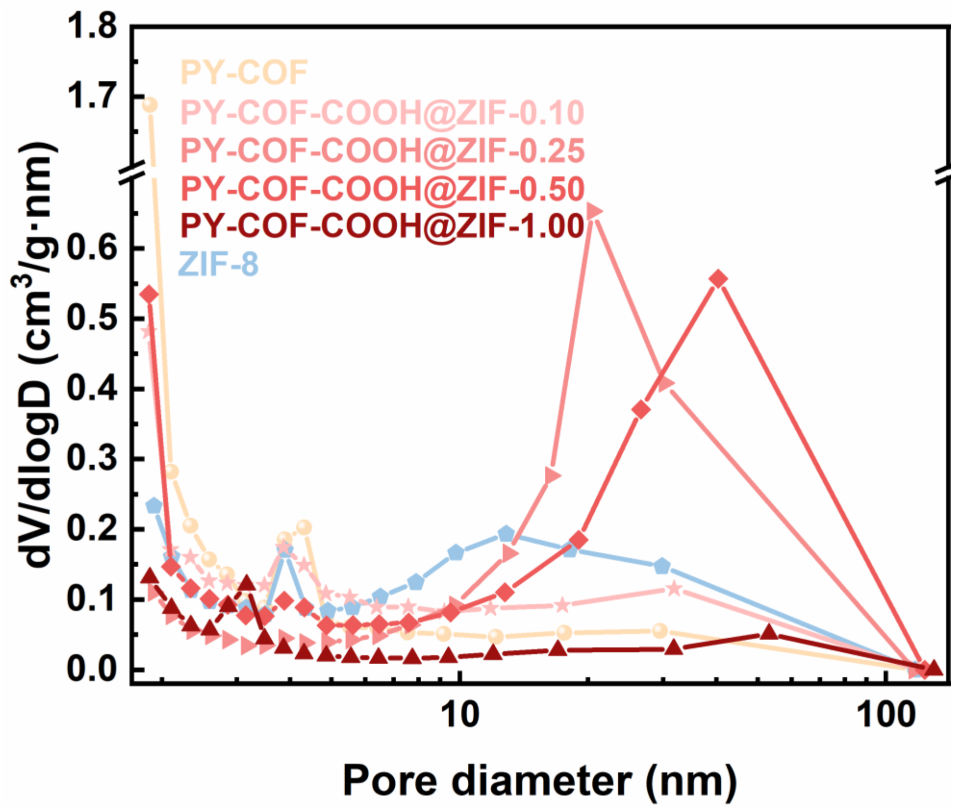

Fig. S52 Maximum accessible aperture of PY-COF-COOH@ZIF-8-X (X=0.10~1.00).

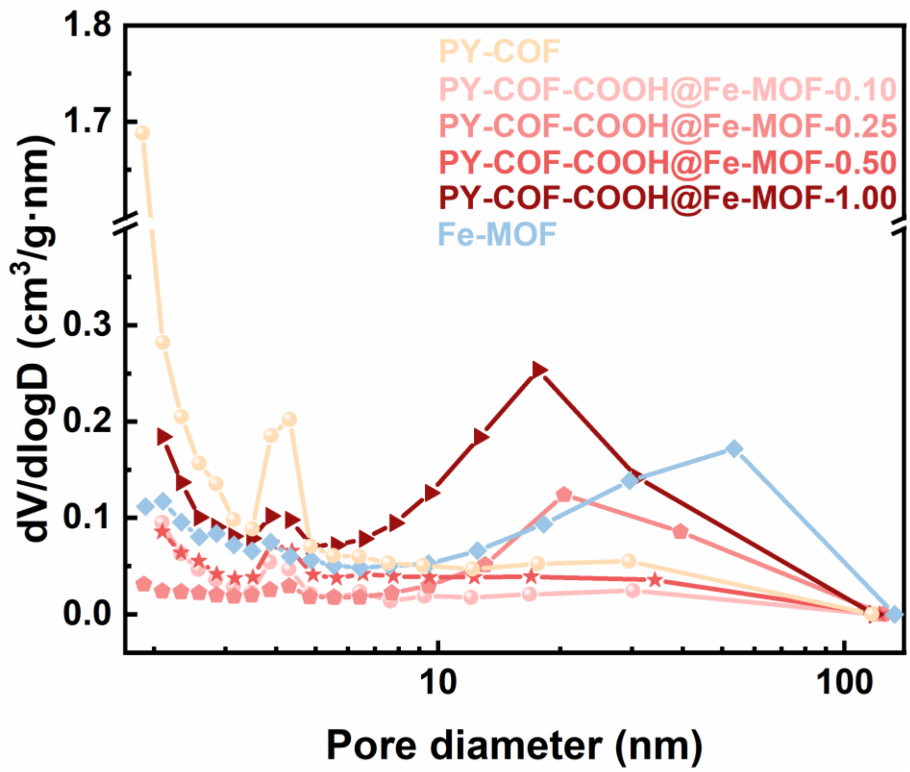

Fig. S53 Maximum accessible aperture of PY-COF-COOH@Fe-MOF-X (X=0.10~1.00).

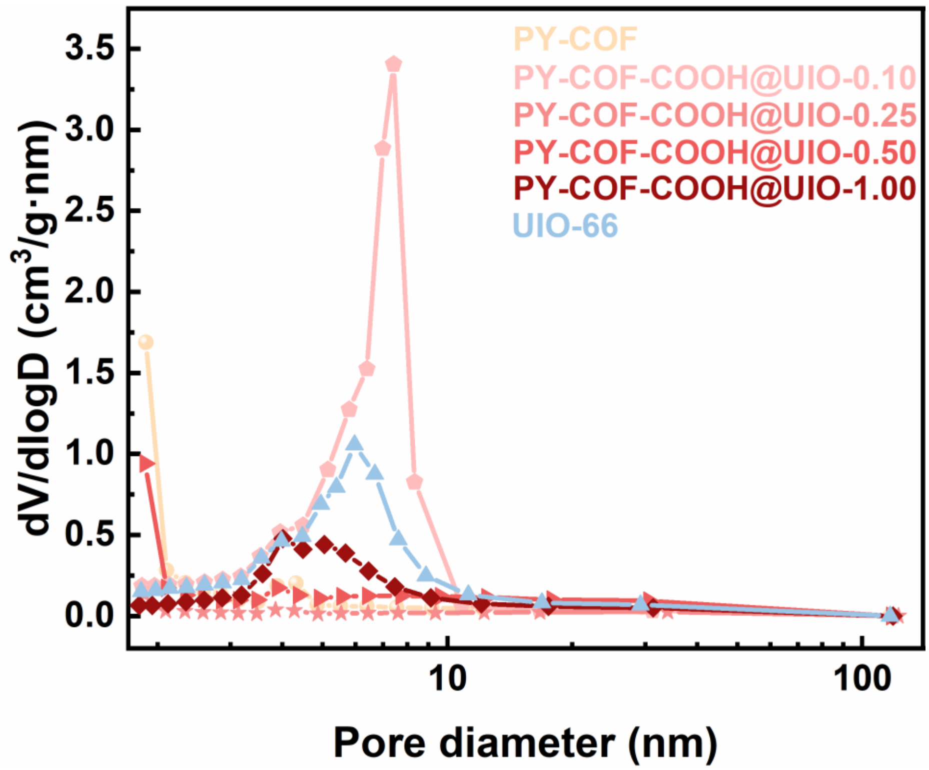

Fig. S54 Maximum accessible aperture of PY-COF-COOH@UIO-X (X=0.10~1.00).

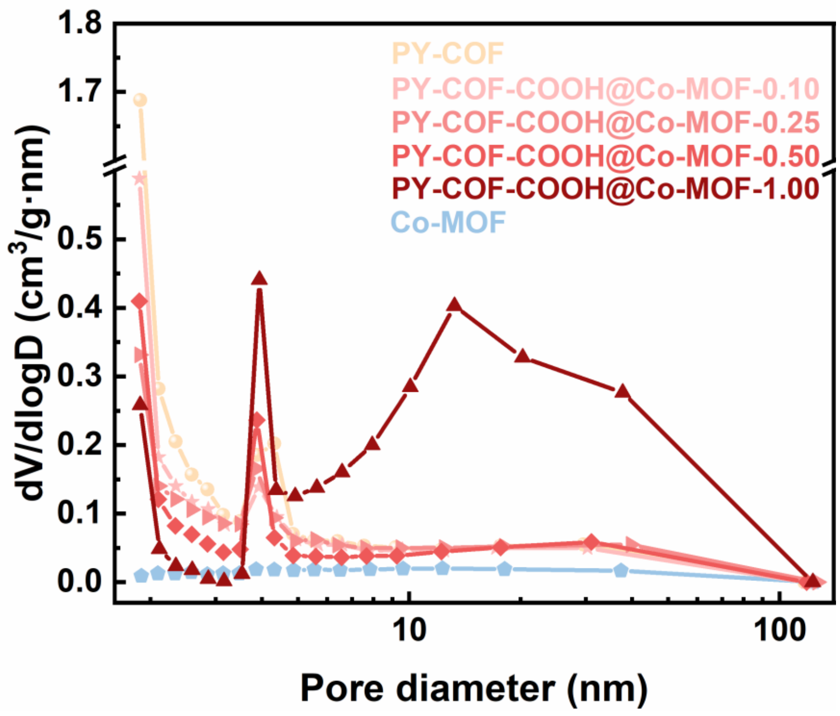

Fig. S55 Maximum accessible aperture of PY-COF-COOH@Co-MOF-X (X=0.10~1.00).

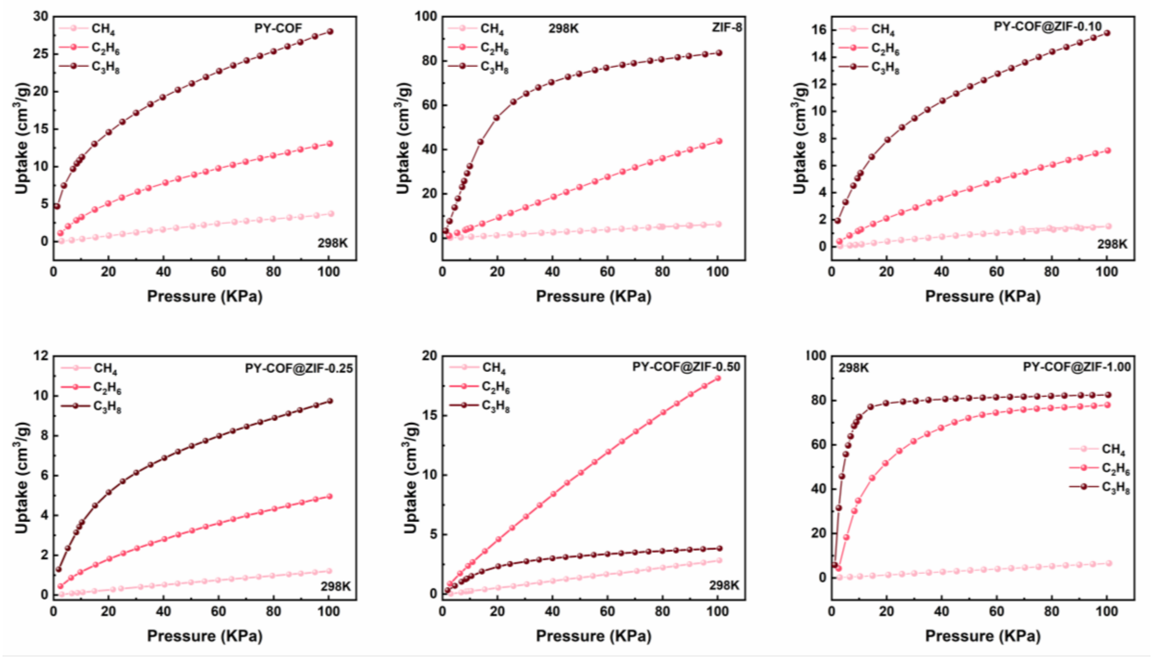

Fig. S56 $CH_4/C_2H_6/C_3H_8$ adsorption isotherm of PY-COF-COOH@ZIF-X (X=0.10~1.00) at 298K

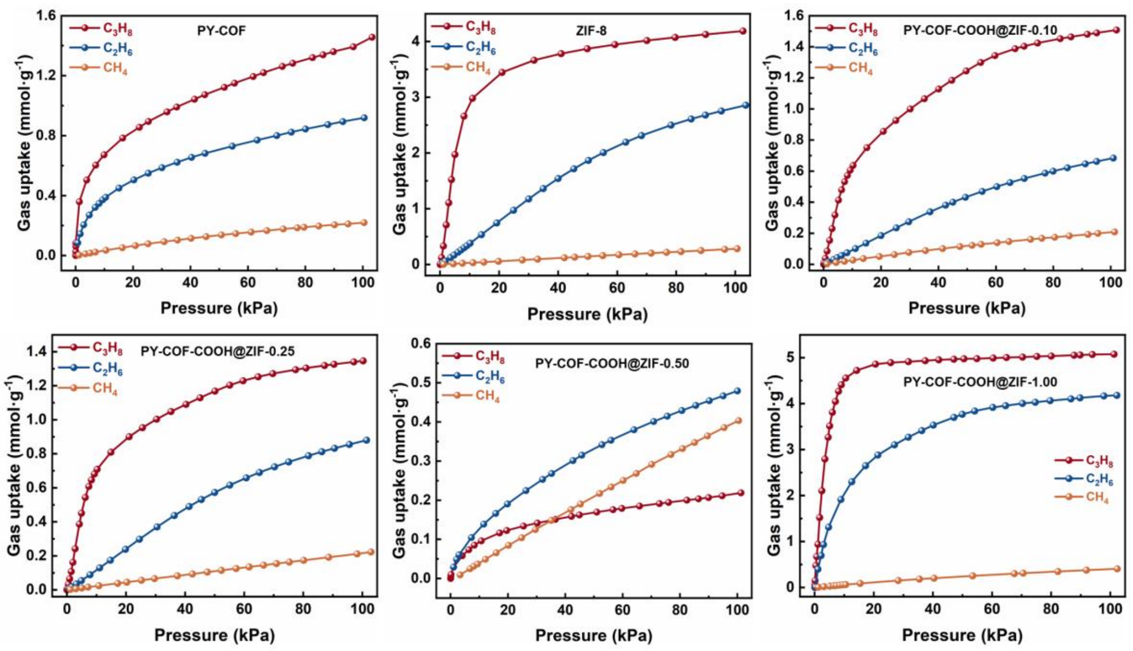

Fig. S57 $CH_4/C_2H_6/C_3H_8$ adsorption isotherm of PY-COF-COOH@ZIF-X (X=0.10~1.00) at 273K

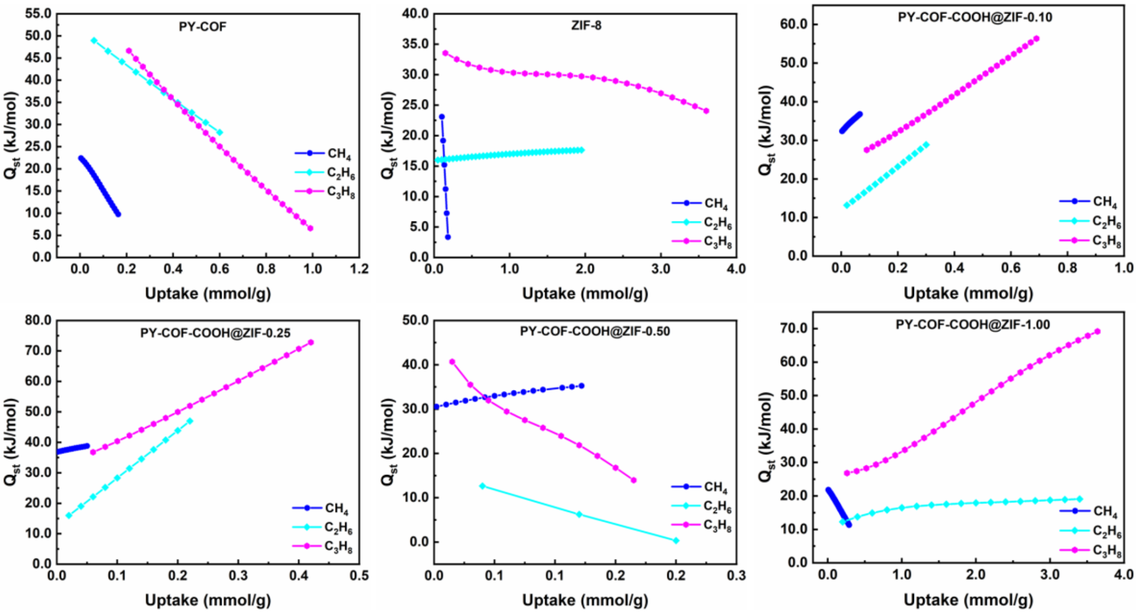

Fig. S58 The Q_{st} of CH_4/C_2H_6/C_3H_8 in PY-COF, ZIF-8 and PY-COF-COOH@ZIF-X (X=0.10~1.00).

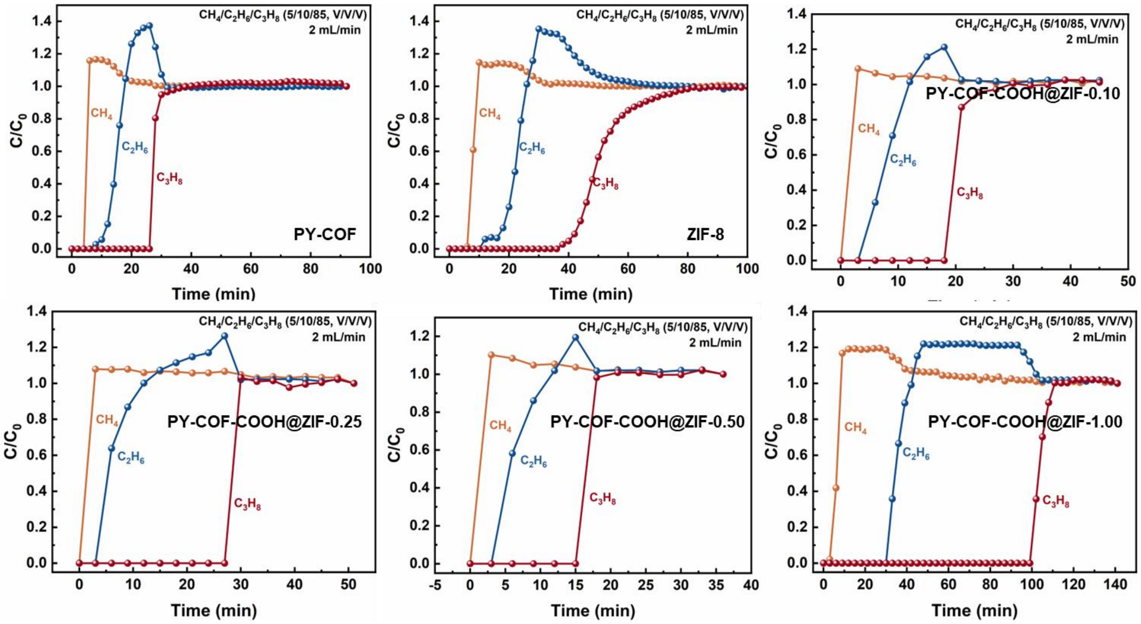

Fig. S59 The experiment breakthrough curves of $CH_4/C_2H_6/C_3H_8$ (5/10/85) gas mixtures with a flow of 2.0 mL/min through PY-COF, ZIF-8 and PY-COF-COOH@ZIF-X (X=0.10~1.00) packed column at 298 K and 100 kPa.

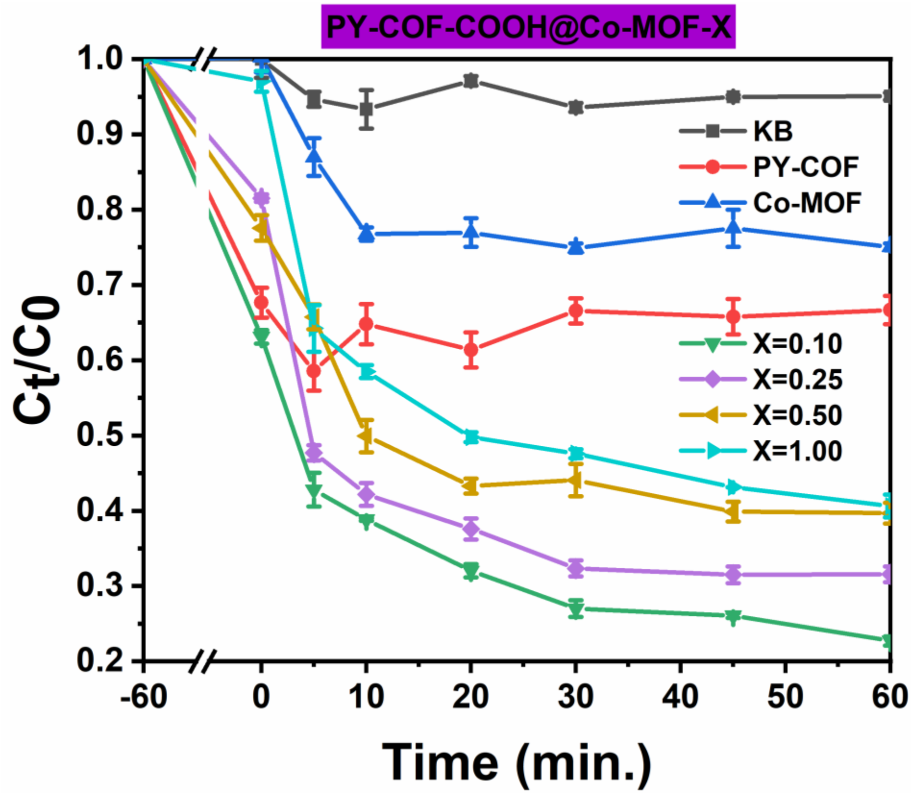

Fig. S60 The Fenton oxidation degradation curve of ENR. (Catalyst: 0.10 g/L, Concentration of ENR: 20 mg/L, Concentration of H_2O_2: 0.03 mol/L).

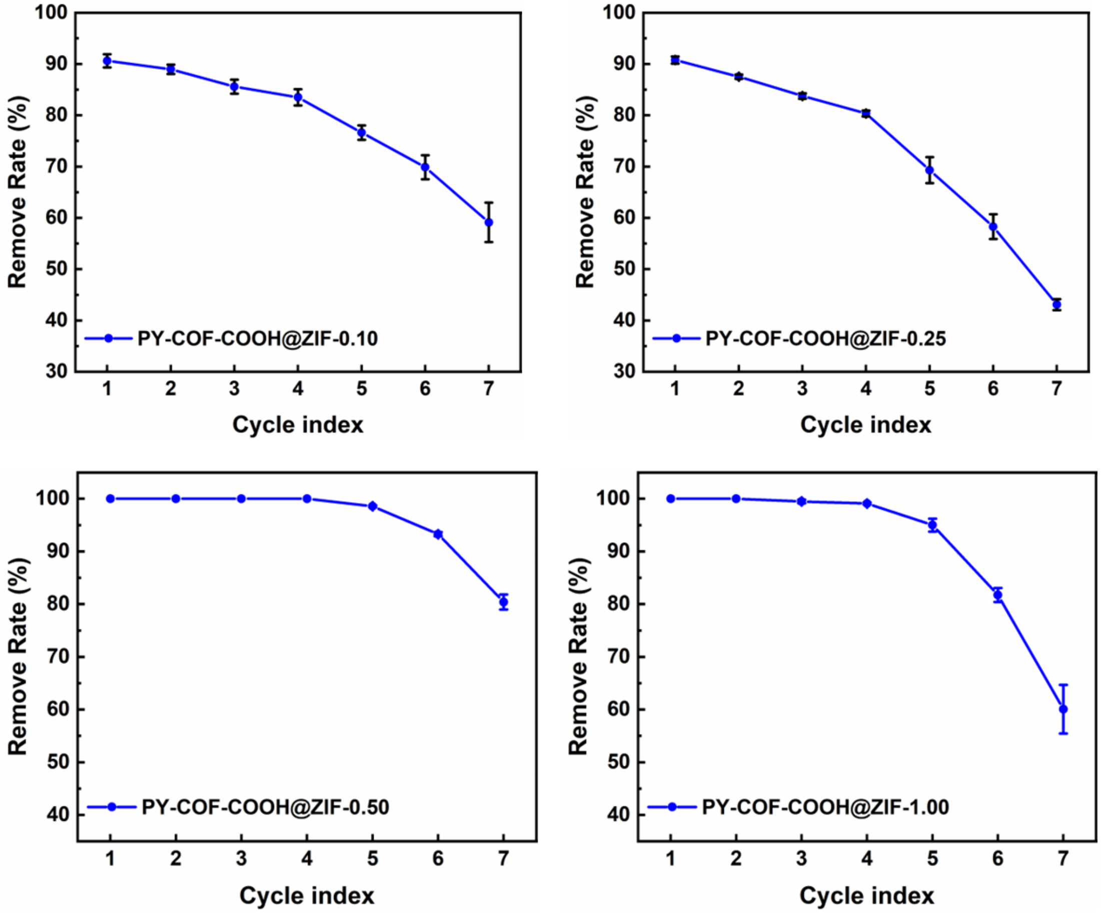

Fig. S61 The reusability of the PY-COF-COOH@Co-MOF-X (X=0.10~1.00)/Fenton system for ENR removal.

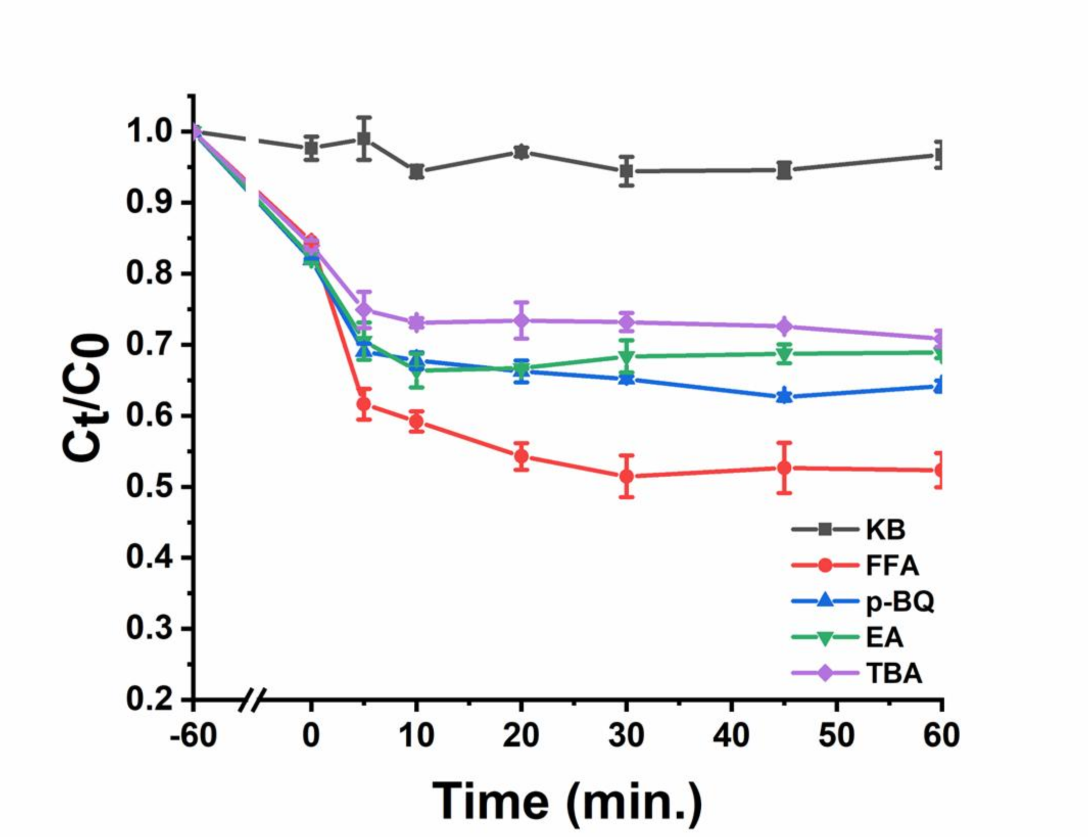

Fig. S62 The result of free radical inhibition experiments of PY-COF-COOH@Co-MOF-0.50.

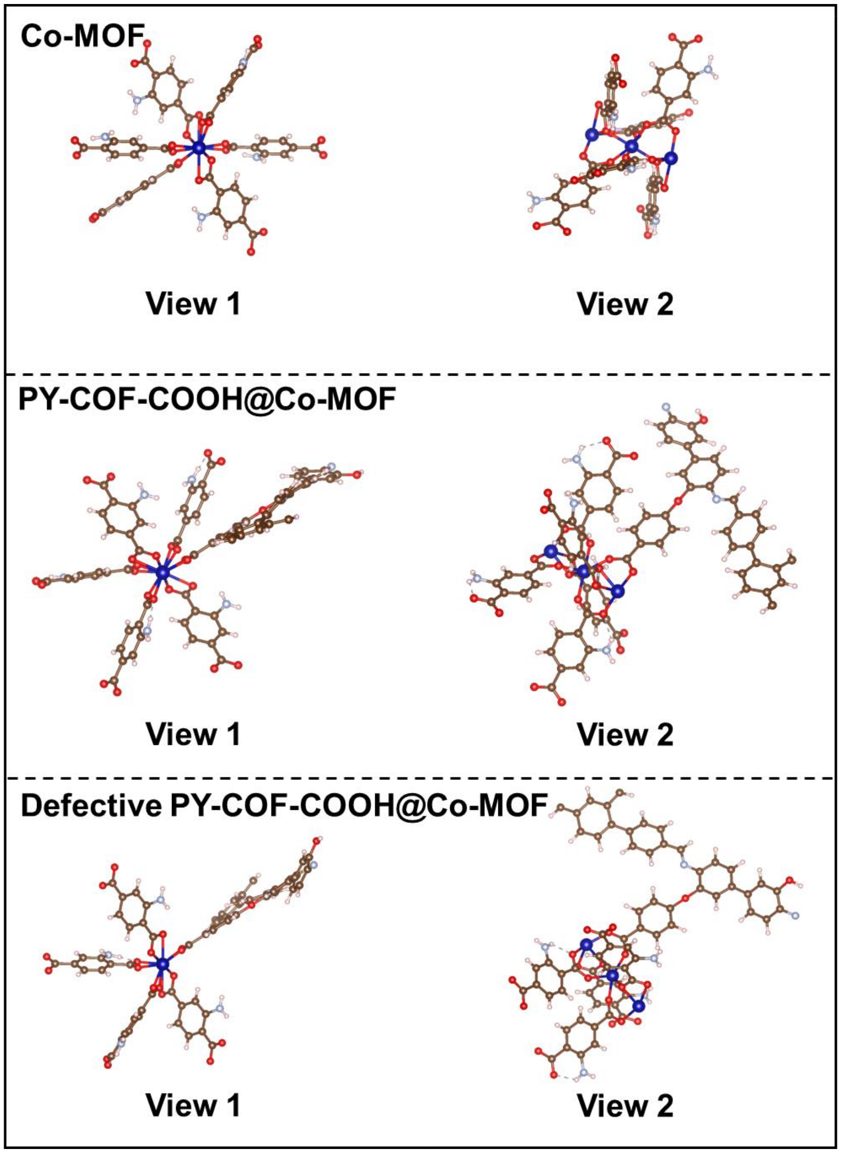

Fig. S63 The model diagram of Co-MOF, PY-COF-COOH@Co-MOF and defective PY-COF-COOH@Co-MOF.

Table S1. EXAFS fitting parameters at the Co K–edge for various samples

Sample	Path	CN	R (Å)	σ^2 ($\times 10^{-3}$ Å^2)	E_0 (eV)	R-factor
Co foil	Co-Co	12	2.49 (0.002)	6.18 (0.23)	7.63 (0.32)	0.001
Co-MOF	Co-O	6.06 (0.44)	2.07 (0.02)	10.00 (0.00)	-2.20	0.0194
PY-COF-COOH@Co-MOF-0.50	Co-O	4.58 (0.42)	1.99 (0.01)	10.57 (1.52)	-0.21	0.005
PY-COF-COOH@Co-MOF-1.00	Co-O	6.22 (0.39)	2.04 (0.01)	7.55 (1.08)	1.33	0.002

[a]*CN*, coordination number; [b]*R*, distance between absorber and backscatter atoms; [c]σ^2, Debye-Waller factor to account for both thermal and structural disorders; [d]ΔE_0, inner potential correction; *R* factor indicates the goodness of the fit. S_0^2 was fixed to 0.747, according to the experimental EXAFS fit of Co foil by fixing CN as the known crystallographic value. A reasonable range of EXAFS fitting parameters: $0.600 < S_0^2 < 1.000$; $CN > 0$; $\sigma^2 > 0$ Å^2; $|\Delta E_0| < 15$ eV; R factor < 0.02.

Table S2. Statistical results of the binding energies of different crystal planes.

MOFs	Crystal plane of PY-COF-COOH	Crystal plane of MOFs	Binding energy (eV)
Co-MOF-COOH	(110)	(020)	-4.53
		(312)	-4.14
		(400)	-3.78
	(220)	(020)	-2.51
		(312)	-2.96
		(400)	-3.23
ZIF-8	(110)	(110)	-1.81
		(211)	-2.22
		(200)	-2.31
	(220)	(110)	-1.44
		(211)	-2.52
		(200)	-1.87

Table S3. BET data of PY-COF-COOH@ZIF-X (X=0.10~1.00).

Sample	S_{BET} ($m^2 \cdot g^{-1}$)	Pore volume ($cm^3 \cdot g^{-1}$)	Average pore size (nm)	Most Frequent Pore Diameter (nm)
PY-COF	486.4626	0.4354	3.5801	1.8744
ZIF-8	1653.4412	0.8866	2.1449	1.9167
X = 0.10	555.8347	0.4695	3.3787	1.8629
X = 0.25	458.2532	0.5697	4.9128	1.9107
X = 0.50	821.5503	0.8303	4.0426	1.8650
X = 1.00	1713.9747	0.6868	1.6028	1.8716

Table S4 BET data PY-COF-COOH@Fe-MOF-X (X=0.10~1.00).

Sample	S_{BET} ($m^2 \cdot g^{-1}$)	Pore volume ($cm^3 \cdot g^{-1}$)	Average pore size (nm)	Most Frequent Pore Diameter (nm)
PY-COF	486.4626	0.4354	3.5801	1.8744
Fe-NH_2-BDC	264.8891	0.3458	5.2218	1.9067
X = 0.10	187.6830	0.1650	3.5166	1.8617
X = 0.25	149.4955	0.1819	4.8670	1.8631
X = 0.50	45.0146	0.1612	14.3242	1.8883
X =1.00	187.6830	0.1650	3.5166	1.8617

Table S5 BET data PY-COF-COOH@UIO-X (X=0.10~1.00).

Sample	S_{BET} ($m^2 \cdot g^{-1}$)	Pore volume ($cm^3 \cdot g^{-1}$)	Average pore size (nm)	Most Frequent Pore Diameter (nm)
PY-COF	486.4626	0.4354	3.5801	1.8744
UIO-66	521.6672	0.6784	5.2018	7.4096
X = 0.10	89.3091	0.0970	4.3445	1.8600
X = 0.25	308.2675	0.3673	4.7660	1.8665
X = 0.50	151.4218	0.2752	7.2698	4.0078
X = 1.00	280.4715	0.4539	6.4734	5.9615

Table S6 BET data PY-COF-COOH@UIO-X (X=0.10~1.00).

Sample	S_{BET} ($m^2 \cdot g^{-1}$)	Pore volume ($cm^3 \cdot g^{-1}$)	Average pore size (nm)	Most Frequent Pore Diameter (nm)
PY-COF	486.4626	0.4354	3.5801	1.8744
Co-NH_2-BDC	13.1513	0.0450	13.6869	2.0928
X = 0.10	328.6172	0.3098	3.7710	1.8646
X = 0.25	367.3752	0.2784	3.0312	1.8647
X = 0.50	377.3105	0.2797	2.9652	1.8649
X = 1.00	159.6907	0.5504	13.7867	1.8748

Table S7 The fitting parameters for Dual−Site Langmuir-Freundlich isotherm model of PY-COF, ZIF-8, PY-COF-COOH@ZIF-X (X=0.10, 0.25, 0.50 and 1.00) at 298 K

Samples	*Alkane*	$q_{A,sat}$	b_A	v_A	$q_{B,sat}$	b_B	v_B	R^2
PY-COF	CH_4	13.6785	0.0031	1.2139	1.1105	0.0290	1.6763	0.9999
	C_2H_6	34.5167	0.0050	0.7672	4.5829e^{-15}	9.4267e^{-14}	6.9106e^{-17}	0.9999
	C_3H_8	15.5576	0.1444	0.6015	55.701	0.0032	0.8944	0.9999
ZIF-8	CH_4	32.9656	0.0026	1.0844	9.3186e^{-14}	1.1989e^{-07}	1.6934e^{-08}	0.9999
	C_2H_6	76.5483	0.0068	1.5991	32.486	0.0105	0.9469	0.9999
	C_3H_8	84.4203	0.0688	1.5237	1.8891	0.8843	44.8484	0.9999
PY-COF-COOH@ZIF-0.10	CH_4	2.8243	0.0105	1.2067	3.0906e^{-16}	35.8787	15.1805	0.9996
	C_2H_6	39.7232	0.0016	0.8465	3.4267e^{-11}	1.0854e^{-09}	0.4780	0.9999
	C_3H_8	22.815	0.0217	0.7895	1.3617	0.0116	5.8798	0.9999
PY-COF-COOH@ZIF-0.25	CH_4	7.5612	0.0020	1.0352	6.9918e^{-17}	2573.27	5.7154e^{-05}	0.9999
	C_2H_6	1.1637	0.1414	0.9436	13.5052	0.0041	1.0312	0.9999
	C_3H_8	16.3965	0.0160	0.7044	4.2304e^{-16}	1.2728e^{-11}	0.0012	0.9999
PY-COF-COOH@ZIF-0.50	CH_4	20.5742	0.0019	1.1202	1.8306e^{-07}	1.9098e^{-21}	1.9816	0.9998
	C_2H_6	26.4551	0.0050	0.7994	23.0004	0.0073	1.6614	0.9999
	C_3H_8	0.0618	1.2191	2.3788e^{-12}	4.2129	0.0548	1.1753	0.9998
PY-COF-COOH@ZIF-1.00	CH_4	0.0073	0.3383	156.69	34.113	0.0027	1.1134	0.9999
	C_2H_6	40.2768	0.0376	2.1846	39.8626	0.1666	2.5361	0.9999
	C_3H_8	80.6212	0.2903	2.3059	6.0653e^{-15}	61.618	11.7926	0.9994

Table S8 Comparison of adsorption capacity (Q) and IAST selectivity at corresponding partial pressure (100 kPa) among the reported MOFs for $C_2H_6/C_3H_8/CH_4$ separation at 298 K.

Materials	IAST of C_2/C_1 10/85 (100kPa)	IAST of C_3/C_1 5/85 (100kPa)	Q_{C2} at 100 kPa (cm^3/g)	Q_{C3} at 100 kPa (cm^3/g)	Ref.
Co-3-AIN	180	2423	58.7	56.4	[15]
Ni-tpt-btb	11	88	93.6	158.7	[16]
FJI-W101b	18.7 (5/5, v/v)	109.6 (5/5, v/v)	57.2	136.66	[17]
Co-1	-	42.94 (5/5, v/v)	-	136.66	[18]
ZUL-C2	82	741	25.98	41.22	[19]
SNNU-126	21.3	235	100.4	99.8	[20]
FJI-C1	-	-	87.4	141.9	[21]
Cu-IPA	40	765	57.57	69.44	[22]
BSF-2	25	681	34.1	49.5	[23]
Co-pyz	23	89	93.4	96.7	[24]
UIO-67	73.7	8.1	8.7	32.0	[25]
Zn-BPDP	32.6	219.3	31.6	36.3	[26]
PY-COF-COOH @ZIF-1.00	55.49	273.86	34.82	55.72	This work

Table S9 Catalytic rate constants K_{obs} of PY-COF-COOH@Co-MOF-X (X=0.10, 0.25, 0.50 and 1.00) in different time periods

Catalysts and its concentration (g/L)		0~5 min	5~10 min	0~10 min	0~60 min
PY-COF	0.10	0.0289	-0.0203	0.0043	0.0002
	0.20	0.0138	-0.0063	0.0038	0.0004
Co-MOF	0.10	0.0283	0.0250	0.0267	0.0048
	0.20	0.0405	0.0176	0.0291	0.0071
X = 0.10	0.10	0.0777	0.0194	0.0486	0.0170
	0.20	0.1632	0.0818	0.1225	0.1382
X = 0.25	0.10	0.1072	0.0245	0.0659	0.0158
	0.20	0.1581	0.0687	0.1134	0.1278
X = 0.50	0.10	0.0331	0.0551	0.0441	0.0112
	0.20	0.1611	0.0703	0.1157	0.1190
X = 1.00	0.10	0.0826	0.0187	0.0507	0.0145
	0.20	0.2062	0.0754	0.1408	0.0865

Table S10 Comparison of Kobs and IAST selectivity at corresponding partial pressure (100 kPa) among the reported MOFs for $C_2H_6/C_3H_8/CH_4$ separation at 298 K.

Materials	AOP	Pollutant	K_{obs} (min^{-1})	Cycle index (Removal rate >80%)	Specific activity (r) $mg \cdot g^{-1} \cdot s^{-1}$ [a]	Ref.
$g\text{-}C_3N_4/BaFe_{12}O_{19}$	Photo-Fenton	ENR	0.499	4	0.1663	[27]
25 % C_3N_4/Fh	Photo-Fenton	ENR	0.1258	5	0.0349	[28]
$ZnO/CuCo_2O_4$	Photo-PMS	ENR	0.0029	4	0.0725	[29]
$CoFe_2O_4$@BC	PMS	ENR	0.22	5	0.3300	[30]
CN-BC-2	PMS	ENR	0.506	6	0.4217	[31]
Co-N/C@EP-600	PMS	OFX[b]	0.289	10	0.2408	[32]
$Bi_{12}FeO_{20}$	Photo-Fenton	CIP[c]	0.349	10	0.1163	[33]
PY-COF-COOH@Co-MOF-1.00	Fenton	ENR	0.2062	6	0.3437	This work

[a] Specific activity (r) = K_{obs}*C_0/$M_{catalyst}$ (C_0 represents the initial concentration of the pollutant, $M_{catalyst}$ represents the mass of the catalyst).

[b] OFX is the abbreviation of ofloxacin.

[c] CIP is the abbreviation of ciprofloxacin.

Reference

[1] Y. Li, L. Yang, H. He, L. Sun, H. Wang, X. Fang, Y. Zhao, D. Zheng, Y. Qi, Z. Li, W. Deng, In situ photodeposition of platinum clusters on a covalent organic framework for photocatalytic hydrogen production, Nat Commun 13 (2022) 1355.

[2] N.P. Bizier, J.W. Wackerly, E.D. Braunstein, M. Zhang, S.T. Nodder, S.M. Carlin, J.L. Katz, An alternative role for acetylenes: Activation of fluorobenzenes toward nucleophilic aromatic substitution, Journal of Organic Chemistry 78 (2013) 5987-5998.

[3] Y. Fu, Y. Yao, A.C. Forse, J. Li, K. Mochizuki, J.R. Long, J.A. Reimer, G. De Paëpe, X. Kong, Solvent-derived defects suppress adsorption in MOF-74, Nat Commun 14 (2023) 2386.

[4 S. Bauer, C. Serre, T. Devic, P. Horcajada, J. Marrot, G. Férey, N. Stock, High-throughput assisted rationalization of the formation of metal organic frameworks in the iron(III) aminoterephthalate solvothermal system, Inorg Chem 47 (2008) 7568–7576.

[5] S. Lee, J.H. Lee, J. Kim, User-friendly graphical user interface software for ideal adsorbed solution theory calculations, Korean Journal of Chemical Engineering 35 (2018) 214-221.

[6] J. Hutter, M. Iannuzzi, F. Schiffmann, J. Vandevondele, Cp2k: Atomistic simulations of condensed matter systems, Wiley Interdiscip Rev Comput Mol Sci 4 (2014) 15-25.

[7] G. Lippert, J. Hutter, M. Parrinello, A hybrid Gaussian and plane wave density functional scheme, Mol Phys 92 (1997) 477-488.

[8] J. Vandevondele, M. Krack, F. Mohamed, M. Parrinello, T. Chassaing, J. Hutter, Quickstep: Fast and accurate density functional calculations using a mixed Gaussian and plane waves approach, Comput Phys Commun 167 (2005) 103-128.

[9] S. Goedecker, M. Teter, Separable dual-space Gaussian pseudopotentials, Phys Rev B Condens Matter Mater Phys 54 (1996). https://doi.org/10.1103/PhysRevB.54.1703.

[10 J.P. Perdew, K. Burke, M. Ernzerhof, Generalized gradient approximation made simple, Phys Rev Lett 77 (1996). https://doi.org/10.1103/PhysRevLett.77.3865.

[11] S. Grimme, J. Antony, S. Ehrlich, H. Krieg, A consistent and accurate ab initio parametrization of density functional dispersion correction (DFT-D) for the 94 elements H-Pu, Journal of Chemical Physics 132 (2010) 154104.

[12] T. Lu, F. Chen, Multiwfn: A multifunctional wavefunction analyzer, J Comput Chem 33 (2012) 580-592.

[13] W. Humphrey, A. Dalke, K. Schulten, VMD: Visual molecular dynamics, J Mol Graph 14 (1996) 33-38.

[14] M. Bajdich, M. García-Mota, A. Vojvodic, J.K. Nørskov, A.T. Bell, Theoretical investigation of the activity of cobalt oxides for the electrochemical oxidation of water, J Am Chem Soc 135 (2013) 13521-13530.

[15] P. Liu, J. Cao, J. Li, H. Li, J. Wang, J. Cai, L. Li, K. Chen, Electrostatic Potential Optimization by Precise Tailoring of Amino Site Arrays in Isostructural Metal-Organic Frameworks for High-Efficiency CH4 Purification, Advanced Functional Materials 36 (2026) e18494.

[16] S. Zhang, N. Qin, H. Huang, Y. Gu, Highly Connected Stable Metal–Organic Frameworks With Polyhedral Cage-Like Cavities for Natural Gas Upgrading, Small 22 (2026) e13848.

[17] C. Chen, M. Li, S. Zou, W. Wei, M. Hong, M. Wu, Reversible Trace-Water-Buffering Frameworks for Efficient Humid Methane Purification, Angewandte Chemie International Edition 64 (2025) e202510669.

[18] S. Liu, L.-N. Zheng, S.-W. Dong, Y.-Z. Sun, Q.-W. Xue, N. Xue, B. Liu, Y. Du, J. Zhao, T. Ding, Novel honeycomb 3D Co/In-MOF with rigid ligand are used for efficient C_1-C_3 light hydrocarbons adsorption separation, fluorescence sensing and selective dye adsorption, Separation and Purification Technology 360 (2025) 130898.

[19] J. Zhou, T. Ke, F. Steinke, N. Stock, Z. Zhang, Z. Bao, X. He, Q. Ren, Q. Yang, Tunable Confined Aliphatic Pore Environment in Robust Metal–Organic Frameworks for Efficient Separation of Gases with a Similar Structure, Journal of the American Chemical Society 144 (2022) 14322–14329.

[20] Y. Liu, J. Wang, S. Fan, C. Xing, Z. Zhong, F. Yuan, W. Yuan, Y. Wang, Q. Zhai, Maximizing the Density of π-Electron in Metal‐Organic Frameworks for Benchmark Paraffin Separation, Advanced Functional Materials 35 (2025) 2504614.

[21] Y. Huang, Z. Lin, H. Fu, F. Wang, M. Shen, X. Wang, R. Cao, Porous Anionic Indium–Organic Framework with Enhanced Gas and Vapor Adsorption and Separation Ability, ChemSusChem 7 (2014) 2647–2653.

[22] D. Lin, S. Tu, L. Yu, Y. Yuan, Y. Wu, X. Zhou, Z. Li, Q. Xia, Highly Efficient Separation of CH_4/C_2H_6/C_3H_8 from Natural Gas on a Novel Copper-Based Metal–Organic Framework, Industrial & Engineering Chemistry Research 62 (2023) 5252–5261.

[23] Y. Zhang, L. Yang, L. Wang, X. Cui, H. Xing, Pillar iodination in functional boron cage hybrid supramolecular frameworks for high performance separation of light hydrocarbons, Journal of Materials Chemistry A 7 (2019) 27560–27566.

[24] L. Zhao, P. Liu, C. Deng, T. Wang, S. Wang, Y.-J. Tian, J.-S. Zou, X.-C. Wu, Y. Zhang, Y.-L. Peng, Z. Zhang, M.J. Zaworotko, Robust ultra-microporous metal-organic frameworks for highly efficient natural gas purification, Nano Research 16 (2023) 12338–12344.

[25] Y. Zhang, H. Xiao, X. Zhou, X. Wang, Z. Li, Selective Adsorption Performances of UiO-67 for Separation of Light Hydrocarbons C1, C2, and C3, Industrial & Engineering Chemistry Research 56 (2017) 8689–8696.

[26] S. Zou, J. Ye, C. Chen, D. Song, J. Yang, M. Wu, Shape‐Matched Nonpolar Pore Surfaces Enhance Alkane Recognition Toward Natural Gas Upgrading, Angewandte Chemie International Edition 137 (2025) e202517133.

[27] G.C. Liu, X.Y. Liu, X.H. Yi, F. Wang, H.Y. Chu, X.H. Xu, Y.M. Zhou, P. Wang, J.F. Wang, C.C. Wang, Fixed-bed catalytic antibiotics detoxification through singlet oxygen-mediated nonradical oxidation: Mechanisms and long-term performance, Water Research 289 (2026) 124791.

[28] J. Deng, J. Chen, Y. Zeng, H. Yang, F. Li, B. Song, Y. Yang, Z. Wang, C. Zhou, W. Wang, Mechanistic insights into ultrafast degradation of electron-rich emerging pollutants by waste cyanobacteria resource utilization, Chemical Engineering Journal 499 (2024) 155918.

[29] X. Wang, J. Wei, H. Zhang, P. Zhou, G. Yao, Y. Liu, B. Lai, Y. Song, $CoFe_2O_4$@BC as a heterogeneous catalyst to sustainably activate peroxymonosulfate for boosted degradation of enrofloxacin: Properties, efficiency and mechanism, Separation and Purification Technology 345 (2024) 127349.

[30] J. Li, X. Cheng, H. Zhang, J. Gou, X. Zhang, D. Wu, D.D. Dionysiou, Insights into performance and mechanism of $ZnO/CuCo_2O_4$ composite as heterogeneous photoactivator of peroxymonosulfate for enrofloxacin degradation, Journal of Hazardous Materials 448 (2023) 130946.

[31] S. Wei, J. Zhang, L. Zhang, Y. Wang, H. Sun, X. Hua, Z. Guo, D. Dong, Efficient generation of singlet oxygen for photocatalytic degradation of antibiotics: Synergistic effects of Fe spin state reduction and energy transfer, Applied Catalysis B: Environmental 358 (2024) 124406.

[32] P. Zhou, Y. Wang, X. Yan, Y. Gan, C. Xia, Y. Xu, M. Xie, Nitrogen-defect-modified g-$C_3N_4/BaFe_{12}O_{19}$ S-scheme heterojunction photocatalyst with enhanced advanced oxidation technology synergistic photothermal degradation ability of antibiotic: Insights into performance, electron transfer pathways and toxicity, Applied Catalysis B: Environmental 343 (2024) 123485.

[33] W. Qiu, J. Liu, H. Shang, Y. Zhang, S. Dou, J. Xu, Y. Zhang, Y. Lou, Y. Zhu, C. Pan, Surface-Bound Superoxide Radical-Mediated Photo-Fenton Mineralization of Ciprofloxacin on Fe-Sillenite Nanosheets, Advanced Science (2026) e22479.
